# Getting up to Speed: A Resident-Led Inpatient Curriculum for New Internal Medicine Interns

**DOI:** 10.15766/mep_2374-8265.10866

**Published:** 2019-12-27

**Authors:** Julia B. Caton, Erin H. Penn, Michelle K. Nemer, Joel T. Katz, Maria A. Yialamas

**Affiliations:** 1 Clinical Assistant Professor, Department of Medicine, Stanford University School of Medicine; 2 Clinical Fellow, Brigham and Women's Hospital; 3 Clinical Fellow, Department of Medicine, Harvard Medical School; 4 Assistant Professor of Medicine, MetroHealth; 5 Assistant Professor of Medicine, Division of General Medical Sciences, Case Western Reserve University School of Medicine; 6 Program Director, Internal Medicine Residency Program, Brigham and Women's Hospital; 7 Associate Professor, Department of Medicine, Harvard Medical School; 8 Associate Program Director, Internal Medicine Residency Program, Brigham and Women's Hospital; 9 Assistant Professor, Department of Medicine, Harvard Medical School

**Keywords:** Internal Medicine, Case-Based Learning, Small-Group Discussion, Peer Teaching, Curriculum, Communication Skills

## Abstract

**Introduction:**

The transition from medical school to internship is an important milestone in medical training and often is a challenge for trainees. This resident-designed and -led inpatient curriculum for internal medicine interns was created to address common clinical scenarios and how best to manage them.

**Methods:**

During the Intern Summer Curriculum, interns participated in weekly small-group sessions facilitated by senior residents. Each case-based session was structured around a clinical topic. Working in pairs with an expert faculty member as a reviewer, volunteer junior and senior residents reviewed and edited each session. During the 2 years we conducted surveys of learners and instructors in the curriculum, there were 200 intern learners and 68 resident instructors.

**Results:**

The Intern Summer Curriculum was evaluated highly by all participants. Of the intern and resident survey responses, 92% (*N* = 77) of interns felt that the curriculum should be continued for future interns, and 100% (*N* = 50) of residents felt that residents should continue to teach in this program. Interns felt that the curricular content helped them better understand topics they commonly encountered and improved their ability to perform day-to-day tasks. Resident instructors felt that teaching in this program was a valuable learning experience and helped strengthen teaching skills.

**Discussion:**

This resident-run inpatient curriculum for internal medicine interns can serve as a valuable learning experience for the intern learners, as well as for the resident instructors, and aid in bringing all interns up to speed at the beginning of intern year.

## Educational Objectives

By the end of this activity, learners will be able to:

1. Describe the presentation, diagnosis, and management of common inpatient clinical scenarios covered in the curriculum.
2. Interpret clinical data in patients with the conditions covered in the curriculum.
3. Practice team and family communication skills.

## Introduction

The transition between medical school and internship is often filled with anxiety, confusion, and concern for medical errors or suboptimal care among interns, residents, program directors, and teaching faculty on inpatient services.^1–3^ There is growing literature on the utility of transition curricula, such as capstone courses offered to fourth-year medical students, to facilitate this progression.^4,5^ However, due to the increasingly variable fourth-year medical school curriculum, interns continue to arrive in residency with different backgrounds and competencies.^6^ For example, in the months preceding internship, some interns may enroll in clinical rotations or do global health work, whereas others may travel or pursue clinical or lab-based research opportunities. These observations and discussion points were made by our senior medical residents during our biweekly house staff meeting with program leadership. They felt that it would be advantageous to bring all interns to the same competency quickly over the summer and that our residency should provide a robust curriculum for high-impact items.

This curriculum was designed to fill this identified gap and facilitate incoming internal medicine intern competency in addressing common inpatient clinical scenarios. Our goal was to provide a series of case-based modules covering the diagnosis and management of clinical conditions that routinely arise on inpatient internal medicine rotations to help accelerate and smooth the transition from medical student to resident. A case-based method was chosen to create an interactive and engaging learning environment and ensure that interns were given the opportunity to practice new skills as part of the learning process.

Reports of graduate medical education curricula are limited to a few intern boot camps in surgical residencies, obstetrics and gynecology, pediatrics, and pediatric cardiology fellowships,^7–13^ as well as reports of a simulation-based boot camp, ambulatory boot camp, and orientation workshops focused on safe handoffs and Accreditation Council for Graduate Medical Education General Competencies for internal medicine interns.^14–17^ This curriculum is unique in that, to our knowledge, there are no published reports of a clinical reasoning–oriented, inpatient-focused transition curricula designed for newly deployed internal medicine interns, including in *MedEdPORTAL.*

## Methods

This curriculum was developed for a target audience of internal medicine interns during their first 3 months of internship. A focus group of senior residents and program leadership developed goals for a PGY 1 transition curriculum for newly deployed interns. The group decided that the focus of the curriculum would be on recognition of commonly encountered core inpatient internal medicine topics, appropriate and efficient management, and team and patient communication skills. The teaching was done in small-group, cased-based sessions. In addition, residents developed and taught the cases. At our institution, the conference was managed by a chief resident and two senior resident leaders in conjunction with a committee of 15–20 junior and senior resident volunteers who developed the curriculum and reviewed and edited content. Each session was reviewed by an expert faculty member at our institution. The core set of topics included in the curriculum were selected by the senior resident leaders under the supervision of a chief resident. Each year, the list of topics included in the curriculum was revised based on intern and resident feedback.

### Implementation

The curriculum consisted of eight 1-hour sessions, which were delivered weekly over the first 3 months of intern year during the noon conference time slot. The topics included in the curriculum were choosing appropriate antibiotics, diagnosis and management of chest pain, discharging patients, evaluation and management of electrolyte abnormalities, management of gastrointestinal bleeding and pancreatitis, inpatient management of diabetic patients, topics in pain and palliative care, and identification and management of shock. For each 1-hour session, the intern class was split into groups of 10, each with two resident teachers. Resident teachers were selected on a volunteer basis and did not receive any specific training outside of the facilitator guide provided for each session. To ensure that all interns had the opportunity to attend, ward senior residents not involved in the teaching sessions were asked to cover the interns’ pagers during the Intern Summer Curriculum time slot.

For each session in the curriculum, we created a learner handout and a facilitator guide (Appendices C–J). The learner handout presented the clinical cases reviewed in the session along with associated discussion questions. The facilitator guide laid out the structure of the workshop and content to be covered and provided suggested answers with explanations for all case-based activities. The resident facilitators received the session materials 1 week prior to each session so that they would have the opportunity to review the content and structure of the session beforehand. During each hour-long session, the two resident facilitators went through a series of cases using the facilitator guide while the interns actively discussed the cases and took notes on their own session handouts. The sessions were conducted in small groups, and the participants in each group were the same from week to week. This allowed for community building within each small group, facilitating a comfortable learning environment where all learners had the opportunity to participate.

### Evaluation

To better understand intern and resident impressions of and experience with the Intern Summer Curriculum, we conducted an anonymous online survey of all interns and residents who participated in the academic year (AY) 2016 and AY 2017 Intern Summer Curriculum sessions. The survey questions assessed the overall impressions and perceived utility of the program for both the intern participants and the resident instructors. The intern survey asked six questions: One was a yes-or-no question, and the other five employed a 5-point Likert scale (1 = *very unfavorable* or *strongly disagree*, 5 = *very favorable* or *strongly agree*; see Appendix A). The resident survey asked five questions: One was a yes-or-no question, and the other four employed a 5-point Likert scale (1 = *strongly disagree*, 5 = *strongly agree*; see Appendix B). All 100 interns (including interns from other services who rotated on medicine during the time the curriculum was running, e.g., medicine-pediatrics, anesthesia, psychiatry) and 34 residents who participated as instructors in the AY 2016 Intern Summer Curriculum were sent an email with a link to the online survey during the spring of 2017. All 100 interns (again including interns from other services who were rotating on medicine during the curriculum) and 34 residents who participated in the AY 2017 Intern Summer Curriculum were sent an email with a link to the online survey during the fall of 2017. All items on the surveys were phrased so that an agree rating was positive. Consequently, the responses recorded on a 5-point Likert scale were coded as follows: 1 = *strongly disagree* or *very unfavorable*, 2 = *disagree* or *unfavorable*, 3 = *neutral*, 4 = *agree* or *favorable*, 5 = *strongly agree* or *very favorable.* For each question, the mean response on the 5-point scale was calculated along with the associated standard deviation.

This project was reviewed by the Partners Healthcare Institutional Review Board and deemed to be exempt (Protocol no. 2017P000793) on April 21, 2017.

## Results

This curriculum has been used at our institution for the past 14 years. All internal medicine interns, as well as interns from other specialties rotating on internal medicine services, participate in the curriculum each year. This amounts to approximately 100 learners per year. We have been able to successfully recruit resident curriculum leaders, resident curriculum editors, and resident instructors each year. Some resident instructors have volunteered to teach during multiple sessions, whereas others teach only once during the curriculum. This is based on resident preference and availability.

For our curricular evaluation survey delivered during AY 2016 and AY 2017, the overall response rate was 50% (134 out of 268); 84 out of 200 (42%) interns and 50 out of 68 (74%) residents responded. Of the interns surveyed, 92% (*N* = 77) of intern respondents felt that the Intern Summer Curriculum should be continued, with 8% (*N* = 7) responding “not sure/indifferent.” No respondents felt that the program should be discontinued. The overall impression of the Intern Summer Curriculum was positive (4.4, *SD* = 0.7). Interns felt that the Intern Summer Curriculum helped them better understand topics they commonly encountered as interns (4.2, *SD* = 0.7) and improved their ability to perform day-to-day intern tasks (3.7, *SD* = 0.8). They believed that the skills and concepts learned in the Intern Summer Curriculum helped them better care for their patients (3.9, *SD* = 0.6) and that the topics addressed were appropriate for their level of training (4.3, *SD* = 0.7; see the Figure). Of the resident teachers surveyed, 100% (*N* = 50) felt that residents should continue to teach in this program. The resident respondents felt that teaching as part of the Intern Summer Curriculum and editing a section of the Intern Summer Curriculum were valuable learning experiences (4.4, *SD* = 0.6; 4.3, *SD* = 0.6). They believed that teaching as part of the Intern Summer Curriculum motivated them to teach more in their role as a resident and helped them hone their teaching skills (3.9, *SD* = 0.8; 3.9, *SD* = 0.8; see the Figure).

**Figure.**
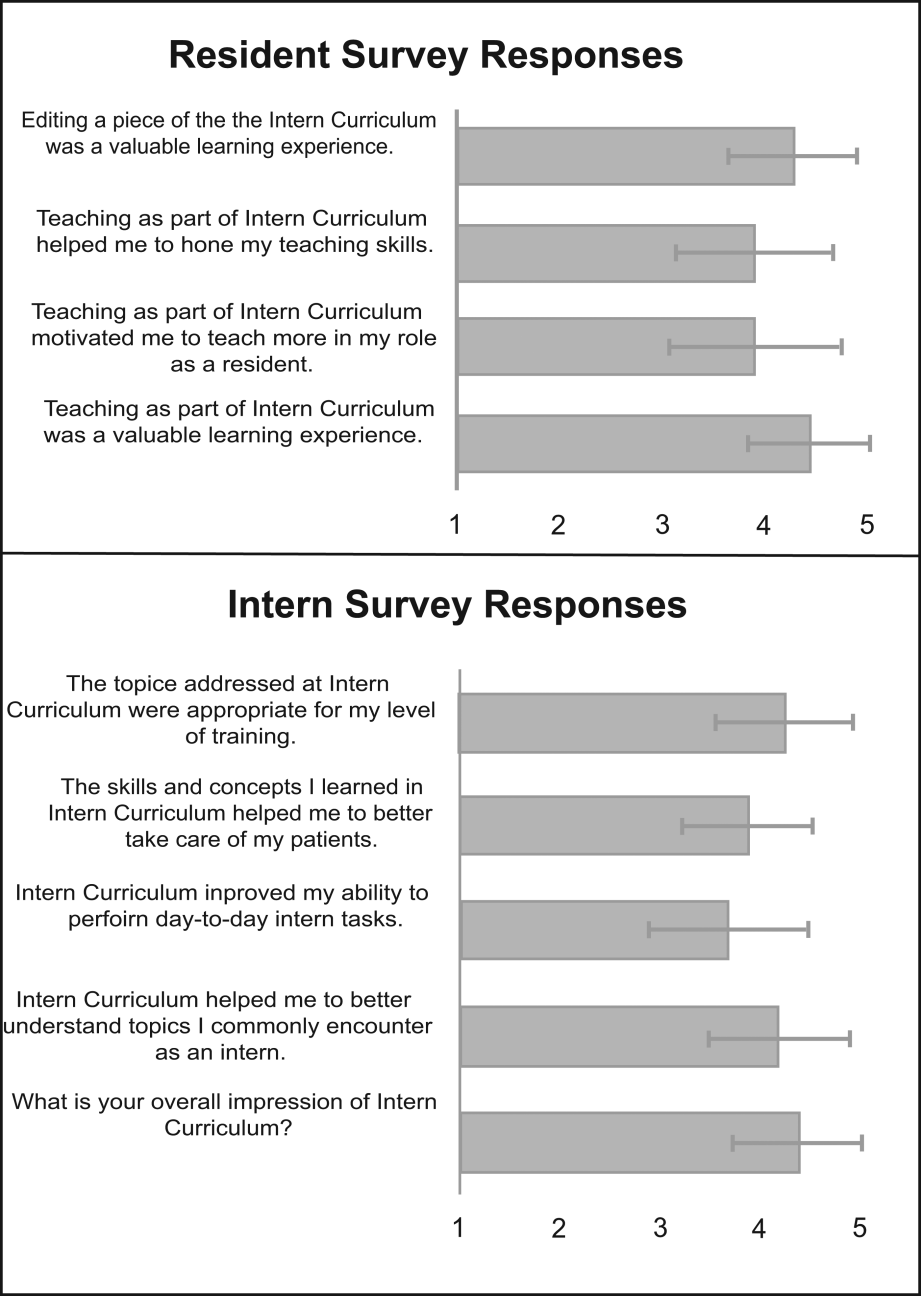
Internal medicine interns and PGY 2 and PGY 3 residents who participated in the Intern Summer Curriculum were surveyed during the 2016–2017 and 2017–2018 academic years about their experiences. All questions were answered on a 5-point Likert scale (1 = *strongly disagree* or *very unfavorable*, 5 = *strongly agree* or *very favorable*). The figure depicts the average numeric response, with error bars showing the standard deviation (*N* = 84 for intern surveys, *N* = 50 for resident responses to questions 1–3, *N* = 23 for resident responses to question 4).

## Discussion

Our Intern Summer Curriculum, which took place early in PGY 1 and was led by senior residents, served as a valuable and enjoyable learning experience for both the intern students and the resident teachers. Our curriculum was unique in that it focused on the recognition, diagnosis, and management of key inpatient clinical scenarios and was taught in a near-peer format. We demonstrated that this program benefited not only interns, by increasing their comfort with common clinical scenarios, but also residents, who found that it strengthened and fostered their skills as clinician educators. Furthermore, this near-peer teaching format also had the potential to build community and enhance camaraderie between interns and residents within the residency program, allowing them to come together in small groups outside of their clinical work.

This transition curriculum, geared toward interns as opposed to fourth-year medical students, had the added benefit of helping learners think through how to approach the topics covered within the confines of a particular specialty and hospital system and the specific practice norms and expectations therein. The resident teachers in this program infused the clinical material in each session with pearls related to specific local practices. For example, in the antibiotics case, resident teachers reviewed our hospital's antibiogram and associated empiric antibiotic choices. In this way, this curriculum may have added benefit above what could have been addressed during the fourth year of medical school.

Furthermore, we found that this resident-run transition curriculum for interns was relatively easy to implement and sustain. In our program, residents readily volunteered to modify the cases each year and teach in the program. In addition to transmission of specific content, this curriculum provided opportunities to set cultural expectations (e.g., when and how to access help) that contributed to a culture of safety and promoted cross-year connections that fostered mentoring, community, and well-being.

Another key lesson we learned through implementing this curriculum was that intern feedback was invaluable in editing the list of topics covered in the curriculum from year to year. For example, over the past several years, a case on electrolyte abnormalities was removed from the curriculum, as interns felt that this material was adequately covered elsewhere in their education. However, cases on discharge planning and palliative care were recently added to the curriculum based on intern feedback.

One potential limitation to the implementation of this curriculum is that it requires multiple resident volunteers to serve as curriculum reviewers and teachers. For each week of the curriculum, we had two resident reviewers who committed substantial time to editing the case several weeks in advance, as well as 10 resident teachers to ensure two instructors per small group. Depending on resident interest and schedules, this may not be feasible at all institutions. The curriculum also requires that interns have protected educational time to attend the sessions. We found that asking residents to cover intern pagers to allow them to attend the curriculum was essential for attendance. Without this protected time, attendance dwindled. Another challenge we encountered was that splitting the intern class into small groups required reservation of multiple conference rooms within the hospital at the same time during the busy lunch hour. Additionally, although our evaluation of the curriculum thus far has focused on learner satisfaction, perceived utility, and perceived learning, future evaluation of the program should include measurements of content learned and impact on patient care.

This Intern Summer Curriculum has implications for how we deliver intern educational sessions going forward. At least at the level of learner satisfaction, the case-based, near-peer format has been well received and could be employed in other educational sessions throughout the year. Our next steps for future iterations of the curriculum will be to continue to find ways to deliver the content to all interns every week. For example, right now, interns who are on night float rotations cannot participate because they are not in the hospital during the day when the sessions are scheduled. We would also like to collect further data related to learning and behavioral outcomes associated with exposure to this curriculum.

## Appendices

A. Intern Survey.docx
B. Resident Survey.docx
C. Acid-Base Disturbances.docx
D. Antibiotics.docx
E. Chest Pain.docx
F. Safe Discharges.docx
G. Gastrointestinal Bleeding and Pancreatitis.docx
H. Inpatient Diabetes Management.docx
I. Pain Management and Palliative Care.docx
J. Shock and Vasopressors.docx All appendices are peer reviewed as integral parts of the Original Publication.
