# Supplementary material for: Getting up to Speed: A Resident-Led Inpatient Curriculum for New Internal Medicine Interns
Source: MedEdPORTAL. 2019 Dec 27;15:10866. doi: 10.15766/mep_2374-8265.10866 (PMC7012307; doi:10.15766/mep_2374-8265.10866)
Supplement: Supplementary file 1 — A. Intern Survey.docx B. Resident Survey.docx C. Acid-Base Disturbances.docx D. Antibiotics.docx E. Chest Pain.docx F. Safe Discharges.docx G. Gastrointestinal Bleeding and Pancreatitis.docx H. Inpatient Diabetes Management.docx I. Pain Management and Palliative Care.docx J. Shock and Vasopressors.docx [file mep-15-10866-s001.zip › A. Intern Survey.docx]

**Post-Curriculum Evaluation Administered to Interns**

1. Which program are you in?
   1. Categorical
   2. Prelim
   3. Primary Care
   4. Anesthesia
   5. Psychiatry
   6. Other _____
2. Did you participate in at least one Intern Curriculum session this year?
   1. Yes
   2. No
3. What is your overall impression of the Intern Curriculum
   1. Very favorable
   2. Somewhat favorable
   3. Neutral
   4. Somewhat unfavorable
   5. Very unfavorable
4. Intern Curriculum helped me to better understand topics I commonly encounter as an intern
   1. Strongly agree
   2. Agree
   3. Neutral
   4. Disagree
   5. Strongly disagree
5. Intern Curriculum improved my ability to perform day to day intern tasks
   1. Strongly agree
   2. Agree
   3. Neutral
   4. Disagree
   5. Strongly disagree
6. The skills and concepts I learned in Intern Curriculum helped me to better take care of my patients
   1. Strongly agree
   2. Agree
   3. Neutral
   4. Disagree
   5. Strongly disagree
7. The topics addressed at Intern Curriculum were appropriate for my level of training
   1. Strongly agree
   2. Agree
   3. Neutral
   4. Disagree
   5. Strongly disagree
8. Should Intern Curriculum be continued for future interns?
   1. Yes
   2. No
   3. Not sure/indifferent
