# Supplementary material for: Getting up to Speed: A Resident-Led Inpatient Curriculum for New Internal Medicine Interns
Source: MedEdPORTAL. 2019 Dec 27;15:10866. doi: 10.15766/mep_2374-8265.10866 (PMC7012307; doi:10.15766/mep_2374-8265.10866)
Supplement: Supplementary file 1 — A. Intern Survey.docx B. Resident Survey.docx C. Acid-Base Disturbances.docx D. Antibiotics.docx E. Chest Pain.docx F. Safe Discharges.docx G. Gastrointestinal Bleeding and Pancreatitis.docx H. Inpatient Diabetes Management.docx I. Pain Management and Palliative Care.docx J. Shock and Vasopressors.docx [file mep-15-10866-s001.zip › C. Acid-Base Disturbances.docx]

Acid-Base Disturbances

Intern Guide

Objectives

At the conclusion of this activity, participants will be able to:

1. Determine when it is useful to perform an ABG
2. Describe a general approach to understanding acid-base disturbances
3. Interpret clinical scenarios where an acid-base disturbance is present

ACID-BASE DISTURBANCES - GENERAL APPROACH

1. What is the **pH?** Is there an acidemia (pH < 7.35) or alkalemia (pH > 7.45)?
2. What is the **primary process?** Respiratory vs Metabolic
3. Is the **compensation appropriate?** If not, there is a second disorder.
4. What is the **anion gap?** (corrected for hypoalbuminemia)
   1. If there is an **anion gap metabolic acidosis** 🡪 **calculate delta-delta** to assess for mixed acid-base d/o
   2. If there is **non-anion gap metabolic acidosis 🡪 calculate urine gap** to assess for renal response
5. Identify a **cause**

**REFERENCE MATERIALS**

**ABG format: pH / pCO2 / PO2 (**helpful to include HCO3 and inhaled O2 if data available)

**Normal Values**

pH 7.40 (range 7.35-7.45)

pCO2 40 (range 35-45)

HCO3 25 (range 22-28)

Anion gap 12 (range 5-17)

**Formulas you may need** *(Recommend reviewing only those in bold):*

| **Compensation Formulas** | |
| --- | --- |
| Metabolic acidosis | **pCO_2_ = 1.5 [HCO_3_] + 8 (+/- 2) (Winter’s Formula)**  pCO_2_ ↓ 1.2 for each ↓ HCO_3_by 1  pCO_2_ ≈ last two digits of pH |
| Metabolic alkalosis | pCO2 = 0.7 x HCO3 + 21  pCO_2_ ↑ 0.7 for each ↑ in HCO_3_ by 1 |
| Respiratory acidosis | Acute: HCO_3_ ↑ 0.1 for every ↑ by 1 of pCO_2_  (Each 1mmHg ↑ PCO2 → 0.007 ↓ pH)  Chronic: HCO_3_ ↑ 0.35 for ↑ by 1 of pCO_2_  (Each 1mmHg ↑ PCO2 → 0.003 ↓ pH) |
| Respiratory alkalosis | Acute: HCO_3_ ↓ 0.2 for every ↓ by 1 of pCO_2_  Chronic: HCO_3_ ↓ 0.5 for every ↓ by 1 of pCO_2_ |

**Anion Gap = [Na^+^] – [Cl^-^] - [HCO_3_^-^]**

**AG correction for albumin:**

- Albumin is a large component of uncalculated anions i.e. the normal AG
- **For every 1 g/dl decline in serum albumin, *expected* AG is approximately ~ 2.5 mEq/L lower**
- 2 ways to manage – either add a correction factor to your calculated AG or adjust what is your expected AG
  - AG_Corrected_ = 2.5(Albumin_Normal_ – Albumin_Serum_) + AG_Calculated_

-- OR --

- - AG_Expected_ = 2.5(Albumin_Serum_); then compare AG_Expected_ to AG_Calculated_

Osmolar gap = Measured Serum Osm - Calculated Serum Osm

- (Normal < 10)
- Calculated Serum Osmolarity **=** 2 [Na] + glucose/18 + BUN/2.8 + EtOH/4.3

**Delta/Delta = ΔAG/ΔHCO3 =** (AG_calculated_ – AG_Normal_)/( [HCO_3_] _Normal_ -[HCO_3_] _Measured_)

**If Δ AG/ Δ HCO3 < 1, additional non-anion gap acidosis**

**If Δ AG/ Δ HCO3 > 2, additional metabolic alkalosis**

If Δ AG/ Δ HCO3 between 1 and 2, indeterminant

NB: some people conceive of the delta-delta as a subtraction: ΔAG - ΔHCO3. This is conceptually the same idea but the math is different

Urine Anion Gap: Na^+^_urine_ + K^+^_Urine_ – Cl^-^_Urin_

**Brief Differential Diagnosis for Common Acid-Base Disorders**

| Respiratory Alkalosis  (Hyperventilation) | Respiratory Acidosis  (Hypoventilation) | Metabolic Alkalosis | Metabolic Acidosis (Non-Gap) | Metabolic Acidosis (AG) |
| --- | --- | --- | --- | --- |
| CNS Activation  Hypoxia  Anxiety  Drugs (ASA,  Theophylline)  Lung Stimulation Asthma  Pneumonia, Pulmonary edema  PE  Liver failure  Sepsis | CNS depression  Opiates  O_2_ in CO_2_ retainer  Neuromuscular  Guillain–Barré  Myasthenia gravis  hypokalemia  Hypophosphatemia  Fatigue  Chest Wall  Kyphosis  Myasthenia crisis  Guillain-Barre  Airway Obstruction Asthma  COPD | GI Losses  Vomiting  NG suction  Renal H+ loss  Diuretics  Bartter’s  Gittelman's  Hyperaldosteronism  Exogenous  Milk alkali syndrome  Volume depletion  Contraction alkalosis | Extra-renal:  Lower GI output  Large volumes of Cl- rich fluids (NS)  Renal causes (RTA’s)  Type I  Type II Type IV  CRI | **Four buckets:**  1. Lactate  2. Ketoacids  3. Ingestions (Osmolar gap)  4. Renal failure  (Dx of exclusion) |

# Anion Gap Mnemonics

| **MUDPILERS** | **GOLDMARK** |
| --- | --- |
| **M**ethanol  **U**remia  **D**KA (or EtOH ketoacidosis)  **P**araldehyde  **I**NH, **I**ron  **L**actate  **E**thanol, **E**th glycol  **R**habdomyolysis  **S**alicylates | **G**lycols (ethylene and propylene)  **O**xyproline  **L**-lactate (not enough oxygen to tissues)  **D**-lactate (albuterol, malignancy, SIBO)  **M**ethanol  **A**spirin  **R**enal failure (from uremia or other organic acid buildup)  **K**etoacidosis (diabetic or EtOH) |

# Lactic acidosis classes

Type A: Hypoperfusion

Type B: No evidence of overt hypoperfusion

- B1: Systemic disease (renal or hepatic failure, diabetes, malignancy)
- B2: Drugs/toxins (albuterol, biguanides, iron, isoniazid, zidovudine, salicylates)
- B3: Inborn errors of metabolism

# Case #1

A 40-year-old man presents with deep, rapid respirations. He has history of a recent infection and hypovolemia due to poor PO intake. His past medical history includes poorly controlled diabetes and hypertension. He takes insulin, lisinopril, and simvastatin. His serum chemistries and ABG are:


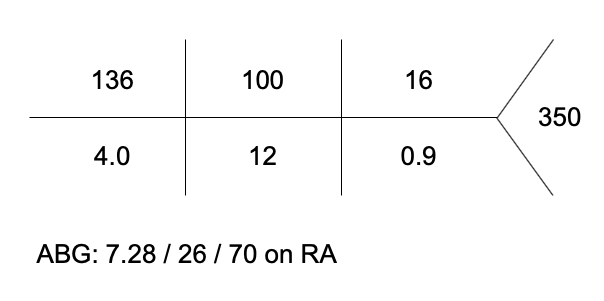


1. **What is the pH?**
2. **What is the primary process?**
3. **Is the compensation appropriate?**
4. **What is the anion gap?** **Is there a mixed acid-base disorder?**

1. **Identify a cause.**

C**ase #2**

A 40-year-old man presents to urgent care after being “sick as a dog” for one week. He has no medical problems and takes no medications. His main symptoms are severe and persistent vomiting. His physical exam is notable only for the fact that he looks tired. Labs are:


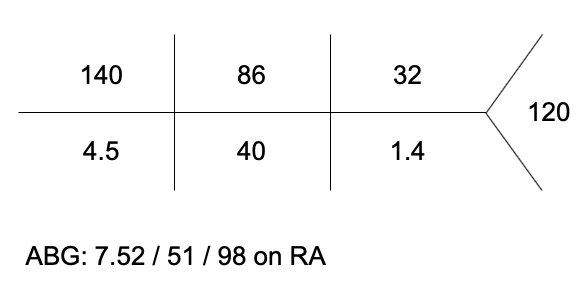


1. **What is the pH?**
2. **What is the primary process?**
3. **Is the compensation appropriate?**
4. **What is the anion gap?** **Is there a mixed acid-base disorder?**

1. **Identify a cause.**

**Case #3**

A 75-year-old male patient at the VA presents with chest pain. His past medical history includes diabetes, COPD, hypertension, CAD, peripheral vascular disease, and BPH. He takes atenolol, lisinopril, insulin, terazosin, atorvastatin, and albuterol/ipratropium bromide inhalers. Labs are obtained in the ED which show:


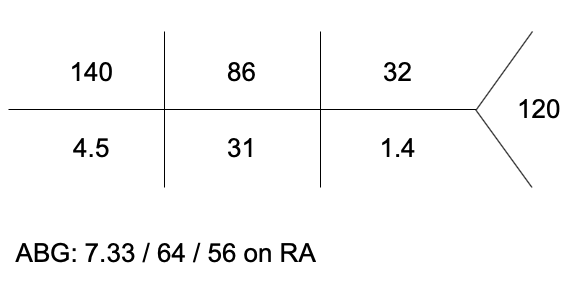


1. **What is the pH?**
2. **What is the primary process?**
3. **Is the compensation appropriate?**
4. **What is the anion gap?** **Is there a mixed acid-base disorder?**

1. **Identify a cause.**

# Case #4

A 65-year-old man is found down in a pool of vomit by his nephew, who reports that his uncle has been “locked away in his house for almost a week” since the death of his beloved cat, Earl. The patient is brought to the ED with sinus tachycardia to 128 and a blood pressure of 85/52. His past medical history includes hypertension, osteoarthritis, and alcohol dependence. Medications are amlodipine and ibuprofen.

His labs:


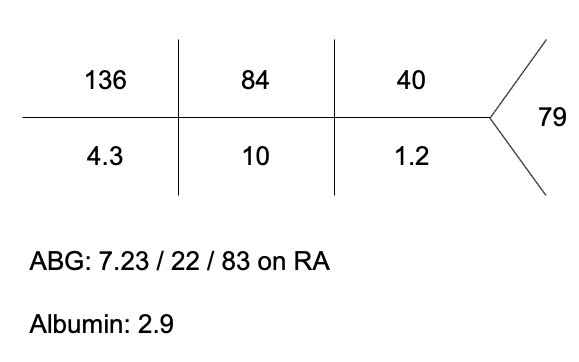


1. **What is the pH?**
2. **What is the primary process?**
3. **Is the compensation appropriate?**
4. **What is the anion gap?** **Is there a mixed acid-base disorder?**

1. **Identify a cause.**

# Case #5

A 37-year-old HIV+ man presents with 3 days of cough productive of thick yellow sputum, shortness of breath, pleuritic chest pain, fever and chills. He has been taking opioids for pain relief. He is using all his accessory muscles to breathe and looks fatigued. His chest x-ray has a dense infiltrate in his right upper lobe.

His labs:


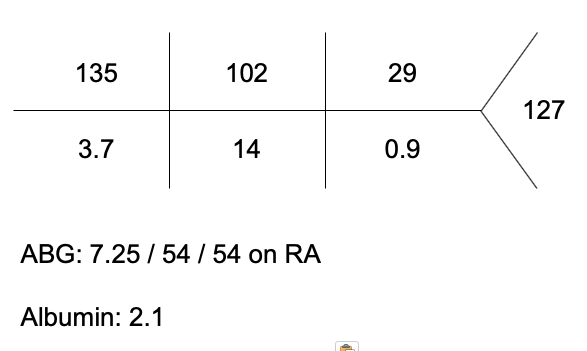


1. **What is the pH?**
2. **What is the primary process?**
3. **Is the compensation appropriate?**
4. **What is the anion gap?** **Is there a mixed acid-base disorder?**

1. **Identify a cause.**

Acid-Base Disturbances

Instructor Guide

Objectives

At the conclusion of this activity, participants will be able to:

1. Determine when it is useful to perform an ABG
2. Describe a general approach to understanding acid-base disturbances
3. Interpret clinical scenarios where an acid-base disturbance is present

*Note: Italicized text does not appear in the intern guide.*

***When to do an ABG?***

*(We recommend reviewing this while people are still trickling in to the session)*

In general, if you think about doing an ABG, you probably should go ahead and do one.

ABGs can yield lots of information and be helpful when:

1) Making diagnoses (e.g. acid/base disturbances, respiratory disorders)

2) Determining the severity of known illnesses (e.g. acidemia in shock, renal failure)

3) Determining if acid/base disturbances are causing problems in and of themselves (e.g. pressor resistance in severe acidemia)

Specific Scenarios:

- *Suspected acidosis*
  - *i.e. low bicarb, anion gap, tachypnea as a compensatory response*
- *Increasing patient somnolence/suspected CO2 retention*
  - *CO_2_ retention is a common cause of somnolence*
  - *This highlights why it is important to include in sign-outs any abnormal baseline mental status, i.e. A&Ox4, confused and occasionally combative, pleasantly demented, etc.*
- *Hypoxemia*
  - *Remember that pulse oximetry may be inaccurate in cases of peripheral vasoconstriction, shock, patients wearing nail polish, methemoglobinemia etc. An actual arterial pO2 will help determine patient’s actual level of hypoxemia.*
- *If you need to determine electrolytes quickly (i.e. a potassium unexpectedly comes back at 7 in a cardiomyopathy patient)*

***INSTRUCTOR PEARLS ON ACID BASE PHYSIOLOGY***

*(We recommend reviewing as much or as little of this part as you see fit and based on interest of the interns in your group)*

***Biochemistry Basics***

## *Acids donate H+ and bases accept H+*

## *Buffering is a solution’s ability to withstand change in pH when an acid or alkali are added*

## *The body tries to keep serum pH around 7.40 using a bicarbonate buffer system (and, to a lesser extent, anionic proteins and PO_4_ buffer)*

### *H^+^ + HCO_3_- ↔ H2CO3 ↔H2O + CO2*

***Systemic arterial pH is maintained between 7.35 and 7.45 by the following mechanisms:***

*Extracellular and Intracellular buffering*

*-K and H+ exchange, plasma proteins, organic and inorganic phosphates (bone)*

*Respiratory regulatory mechanisms*

*-PaCO2 regulated by neural respiratory system (not subjected to regulation by rate of CO2 production)*

*Renal regulatory mechanisms*

*-regulate HCO3 by reabsorption of filtered HCO3^-^, formation of titratable acids, and urinary excretion of NH4^+^ /daily acid load.*

ACID-BASE DISTURBANCES - GENERAL APPROACH

*What information do I need to solve an acid-base problem?* ***ABG (review shorthand 7.4/40/99), Chem7***

1. **What is the pH?** Is there an acidemia (pH < 7.35) or alkalemia (pH > 7.45)?
2. **What is the primary process?** Respiratory versus Metabolic
   1. *Respiratory primary: acidosis (low pH) has high pCO2, alkalosis (high pH) has low pCO2 (pH and pCO2 move in opposite directions)*
   2. *Metabolic primary: acidosis (low pH) has low HCO3, alkalosis (high pH) has high HCO3 (pH and HCO3 move in same direction)*
3. **Is the compensation appropriate?** If not, there is a second disorder.
4. **What is the anion gap? (corrected for hypoalbuminemia)**
   1. *Narrows your differential diagnosis to gap versus non-gap acidosis etiologies. Notably, whenever there is an AG > 20, there is almost undoubtedly a metabolic acidosis, it may just be “hidden” by a normal bicarb because of an additional acid-base disorder*
   2. *Remember to correct anion gap for hypoalbuminemia. A normal anion gap is 2.5 x the albumin (this is where we derive a normal AG of 10, assuming an albumin of 4).*
5. **If there is an anion gap metabolic acidosis** 🡪 **calculate delta delta** (**ΔAG/ΔHCO3)** to assess for mixed acid-base d/o
   1. *Instructors: can be a tricky topic. Some people use delta/delta (the ratio approach), some use delta-delta (the subtraction approach). Included are explanations for both. Explain whatever you’d like.*
   2. *Concept of the “delta delta” - In the case of a pure anion gap metabolic acidosis, when one molecule of metabolic acid (HA) is added to blood, the one H+ ion that is released reacts with one molecule of HCO3-, and* *the change in both the anion gap and bicarbonate should be approximately equal and their ratio should be equal to 1 (or subtracting one from the other should yield a very small number).*
   3. *Delta/delta (ratio approach):*
      1. *If there is an additional non-anion-gap acidosis happening, then there will be greater change in the bicarbonate than in the anion gap (more acid than what is captured by our crude anion gap). Consequently, the delta AG (numerator) will be smaller than the delta HCO3 (denominator), and the delta/delta will be <1.*
      2. *If there is an additional alkalosis happening, then there will be smaller change in the bicarbonate than in the anion gap (because the bicarbonate will decrease due to consumption with acid, but then it will also increase because of the concurrent alkalosis). Consequently, the delta AG (numerator) will be larger than the delta HCO3, and the delta/delta will be >1 (really needs to be greater than 2 to be significant).*
   4. *Delta-delta (subtraction approach):*
      1. *If there is an additional non-gap acidosis happening, then there will be greater change in the bicarbonate than in the anion gap (see above). Consequently, the delta AG minus the delta HCO3 will be more negative; if <-5 it is likely that there is an additional non-gap acidosis.*
      2. *If there is an additional alkalosis happening, then there will be smaller change in the bicarbonate than in the anion gap (see above). Consequently, the delta AG minus the delta HCO3 will be a more positive integer; if over >5, it is likely that there is an additional alkalosis.*
6. **If there is non-anion gap metabolic acidosis 🡪 calculate urine gap to assess for renal response** *(Instructors -- may want to defer this subtopic given time constraints and information-overwhelm)*
   1. *Urine gap = (urineNa + urineK ) – urineCl*
   2. *If appropriate renal response to metabolic acidosis, kidney should secrete extra H+ as NH4+, which will be matched with increased Cl- excretion*
   3. *Therefore, in a normal renal response, urine gap should be NEGATIVE – more measured anions (Cl) than measured cations (because we don’t measure the NH4)*
   4. *If kidney is unable to respond appropriately, there will be low NH4 excretion, and the urine gap will be positive.*
7. **Identify a cause.**

# Case #1

A 40-year-old man presents with deep, rapid respirations. He has history of a recent infection and hypovolemia due to poor PO intake. His past medical history includes poorly controlled diabetes and hypertension. He takes insulin, lisinopril, and simvastatin. His serum chemistries and ABG are:


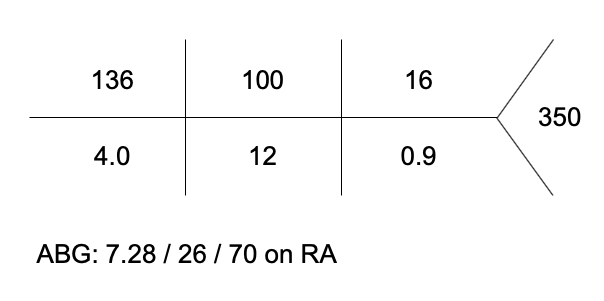


1. **What is the pH?** *7.28, so there is acidemia*
2. **What is the primary process?** *The pCO2 is low, and serum bicarbonate is low, so this is a metabolic acidosis.*
3. **Is the compensation appropriate?** *By Winter’s formula 1.5(12)+ 8 = 26, which means there is appropriate respiratory compensation.*
4. **What is the anion gap?** **Is there a mixed acid-base disorder?** *136-100-12 = 24. This is an AG metabolic acidosis. Now, to look for a hidden disorder, one needs to look at the delta-delta. The delta-delta is (24-12)/(24-12) = 1.0. This means that serum bicarbonate is altered by what we would expect and there is no “hidden” disorder.*
5. **Identify a cause.** *Refer to the differential of an AG metabolic acidosis. This patient is hyperglycemic and a test for serum ketones was positive, so he has DKA.*

*NB – Given hyperglycemia, you may want to calculate a corrected sodium for this patient. However, you use the UNcorrected sodium for anion-gap calculations. More below if people ask:*

*Osmotic Hyponatremia occurs when excess glucose in the intravascular space draws water from the intracellular space, thereby diluting the sodium concentration. TBW remains unchanged, though, and sodium will return to "normal" once the hyperglycemia is corrected.*

*To calculate the corrected serum sodium, add 1.6mEq to the measured serum sodium for every 100 mg/dl of glucose above 100 mg/dl.*

*[Na]corr = [Na]meas +{(glucose – 100 x 1.6)/100}*

*Sodium in this case corrects to about 140. However, the uncorrected serum sodium should be used in calculating the anion gap, so your assessment of the acid-base disorder does not change.*

C**ase #2**

A 40-year-old man presents to urgent care after being “sick as a dog” for one week. He has no medical problems and takes no medications. His main symptoms are severe and persistent vomiting. His physical exam is notable only for the fact that he looks tired. Labs are:


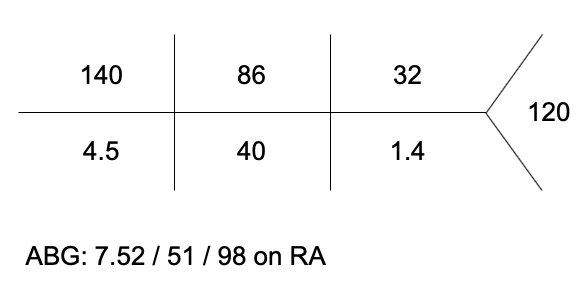


1. **What is the pH?** *7.52, so there is alkalemia*
2. **What is the primary process?** *The pCO2 is high and serum bicarbonate is high, so this is a metabolic alkalosis.*
3. **Is the compensation appropriate?** *The bicarbonate is 40-24 = 16 points higher than it should be. The pCO_2_ should increase by 0.7*16 = 11 or should be 51. There is appropriate compensation.*
4. **What is the anion gap?** **Is there a mixed acid-base disorder?** *There is generally no need to calculate the AG in metabolic alkalemia if the compensation appears appropriate. Here the AG is 140-86-40 = 14. This is not more than 20, making it less likely for a hidden acidosis to exist.* *In alkalemia, loss of protons from plasma proteins (particularly albumin) increases their negative charge. Alkalemia also stimulates lactic acid production, which will increase the anion gap. The increase in AG is usually mild, so even though it is >12, we do not think much of it in the setting of appropriate compensation.*
5. **Identify a cause.** *Refer to the differentials. Again, can think of causes as renal and extra-renal. In this case, the vomiting is why is he has an alkalosis. When the cause is less obvious, can use the urine chloride and the volume status to differentiate causes. Again, this discussion is deferred for time.*

**Case #3**

A 75-year-old male patient at the VA presents with chest pain. His past medical history includes diabetes, COPD, hypertension, CAD, peripheral vascular disease, and BPH. He takes atenolol, lisinopril, insulin, terazosin, atorvastatin, and albuterol/ipratropium bromide inhalers. Labs are obtained in the ED which show:


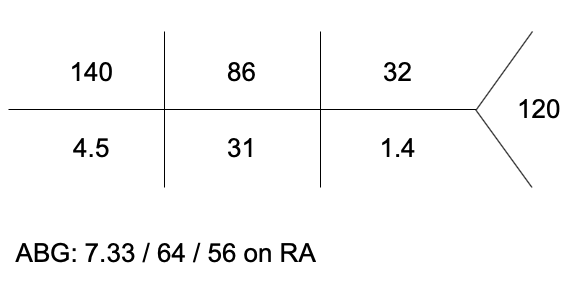


1. **What is the pH?** *7.33, so there is acidemia*
2. **What is the primary process?** *The pCO_2_ is high and serum bicarbonate is high, so there is a primary respiratory acidosis.*
3. **Is the compensation appropriate?** *This patient likely has had COPD for a long time and has a chronic respiratory acidosis. The pCO2 is 64-40 = 24 points higher than it normally would be. Appropriate compensation would be 24*0.3 = 7. The compensated bicarbonate should be 24+7 = 31. This is roughly what the ABG bicarbonate shows, so there is appropriate metabolic compensation.*
4. **What is the anion gap?** **Is there a mixed acid-base disorder?** *140-86-40 = 14. This is not more than 20, making it less likely for a hidden acidosis.*
5. **Identify a cause.** *Refer to the differentials. In this case, the chronic respiratory acidosis is due to his COPD.*

# Case #4

A 65-year-old man is found down in a pool of vomit by his nephew, who reports that his uncle has been “locked away in his house for almost a week” since the death of his beloved cat, Earl. The patient is brought to the ED with sinus tachycardia to 128 and a blood pressure of 85/52. His past medical history includes hypertension, osteoarthritis, and alcohol dependence. Medications are amlodipine and ibuprofen.

His labs:


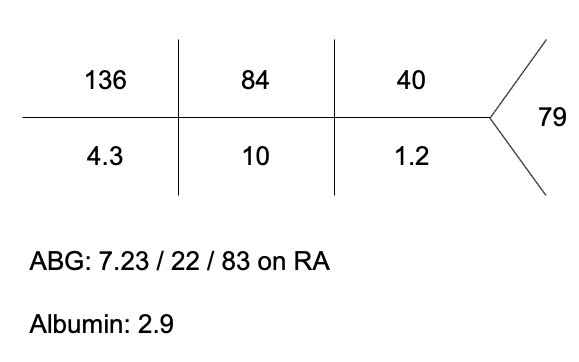


1. **What is the pH?** *The pH is 7.23, so he is acidemic.*
2. **What is the primary process?** *His pCO2 is low and serum bicarbonate is decreased so there is a metabolic acidosis.*
3. **Is the compensation appropriate?** *By Winter’s formula, 1.5(10) + 8 = 23, which means there is appropriate respiratory compensation.*
4. **What is the anion gap?** **Is there a mixed acid-base disorder?** *Remember the anion gap needs to be corrected for albumin. For every drop in albumin by 1 point, 2.5 needs to be added to the AG. So, the AG needs to be corrected by 2.5(4-2.9) = 2.75. The AG is 136-84-10 = 42. Corrected it is 44.75 or about 45.*

*Now, to look for a hidden disorder, one needs to look at the delta-delta. The delta-delta is (45-12)/ (24-10) = 2.3. This means that serum bicarbonate is higher than you would expect if it were purely compensating for the increase in AG. Thus, there must be another process INCREASING the amount of bicarbonate, i.e. a concurrent metabolic alkalosis.*

1. **Identify a cause.** *Refer to the differentials. This a mixed AG metabolic acidosis and metabolic alkalosis, with AG metabolic acidosis being the predominant primary process (given the pH). We can check serum osmolality, lactate, and ketones to suggest a cause. Serum osmolality is useful to see if there is an osmolar gap, which can help narrow the differential for an AG metabolic acidosis. If there is an osmolar gap, there is concern for methanol or ethylene glycol intoxication.*

*The metabolic alkalosis is the lesser of the two processes (since pH is academic) and is likely due to vomiting* *or contraction from being hypovolemic.*

# Case #5

A 37-year-old HIV+ man presents with 3 days of cough productive of thick yellow sputum, shortness of breath, pleuritic chest pain, fever, and chills. He has been taking opioids for pain relief. He is using all his accessory muscles to breathe and looks fatigued. His chest x-ray has a dense infiltrate in his right upper lobe.

His labs:


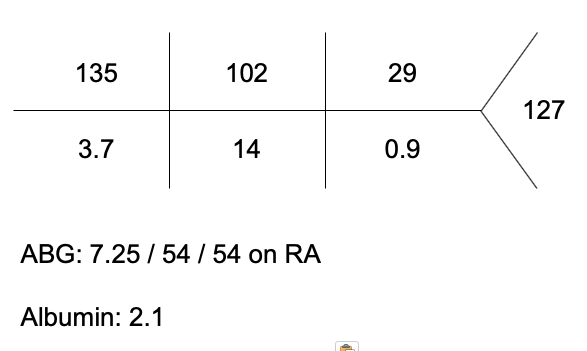


1. **What is the pH?** *The pH is 7.25, so he is acidemic.*
2. **What is the primary process?** *His serum bicarbonate is decreased so there is a metabolic acidosis,* *but his CO2 is also increased, so there is also a respiratory acidosis – see below.*
3. **Is the compensation appropriate?** *By Winter’s formula, 1.5(14) + 8 = 29. The pCO2 instead of being lower than normal to compensate for a metabolic acidosis is higher than normal and certainly much higher than the expected 29, indicating a concurrent respiratory acidosis.*
4. **What is the anion gap?** **Is there a mixed acid-base disorder?** *The AG is 135-102-14 = 19. The correction for albumin is 2.5(4-2.1) = 4.75. So, the AG is about 24. The delta-delta is (24-12)/ (24-14) which is about 1, so there is no additional disorder.*
5. **Identify a cause.** *Refer to the differentials. For his metabolic acidosis, this is most likely the result of a lactic acidosis, but a more thorough work-up is needed. For his respiratory acidosis, this seems to be the results of fatigue* and possibly respiratory depression due to narcotic use.

**REFERENCE MATERIALS**

**ABG format: pH / pCO2 / PO2 (**helpful to include HCO3 and inhaled O2 if data available)

**Normal Values**

pH 7.40 (range 7.35-7.45)

pCO2 40 (range 35-45)

HCO3 25 (range 22-28)

Anion gap 12 (range 5-17)

**Formulas you may need** *(Recommend reviewing only those in bold):*

| **Compensation Formulas** | |
| --- | --- |
| Metabolic acidosis | **pCO_2_ = 1.5 [HCO_3_] + 8 (+/- 2) (Winter’s Formula)**  pCO_2_ ↓ 1.2 for each ↓ HCO_3_by 1  pCO_2_ ≈ last two digits of pH |
| Metabolic alkalosis | pCO2 = 0.7 x HCO3 + 21  pCO_2_ ↑ 0.7 for each ↑ in HCO_3_ by 1 |
| Respiratory acidosis | Acute: HCO_3_ ↑ 0.1 for every ↑ by 1 of pCO_2_  (Each 1mmHg ↑ PCO2 → 0.007 ↓ pH)  Chronic: HCO_3_ ↑ 0.35 for ↑ by 1 of pCO_2_  (Each 1mmHg ↑ PCO2 → 0.003 ↓ pH) |
| Respiratory alkalosis | Acute: HCO_3_ ↓ 0.2 for every ↓ by 1 of pCO_2_  Chronic: HCO_3_ ↓ 0.5 for every ↓ by 1 of pCO_2_ |

**Anion Gap = [Na^+^] – [Cl^-^] - [HCO_3_^-^]**

**AG correction for albumin:**

- Albumin is a large component of uncalculated anions i.e. the normal AG
- **For every 1 g/dl decline in serum albumin, *expected* AG is approximately ~ 2.5 mEq/L lower**
- 2 ways to manage – either add a correction factor to your calculated AG or adjust what is your expected AG
  - AG_Corrected_ = 2.5(Albumin_Normal_ – Albumin_Serum_) + AG_Calculated_

-- OR --

- - AG_Expected_ = 2.5(Albumin_Serum_); then compare AG_Expected_ to AG_Calculated_

Osmolar gap = Measured Serum Osm - Calculated Serum Osm

- (Normal < 10)
- Calculated Serum Osmolarity **=** 2 [Na] + glucose/18 + BUN/2.8 + EtOH/4.3

**Delta/Delta = ΔAG/ΔHCO3 =** (AG_calculated_ – AG_Normal_)/( [HCO_3_]_Normal_ -[HCO_3_]_Measured_)

**If Δ AG/ Δ HCO3 < 1, additional non-anion gap acidosis**

**If Δ AG/ Δ HCO3 > 2, additional metabolic alkalosis**

If Δ AG/ Δ HCO3 between 1 and 2, indeterminate

NB: some people conceive of the delta-delta as a subtraction: ΔAG - ΔHCO3. This is conceptually the same idea, but the math is different

Urine Anion Gap: Na^+^_urine_ + K^+^_Urine_ – Cl^-^_Urin_

**Brief Differential Diagnosis for Common Acid-Base Disorders**

| Respiratory Alkalosis  (Hyperventilation) | Respiratory Acidosis  (Hypoventilation) | Metabolic Alkalosis | Metabolic Acidosis (Non-Gap) | Metabolic Acidosis (AG) |
| --- | --- | --- | --- | --- |
| CNS Activation  Hypoxia  Anxiety  Drugs (ASA,  Theophylline)  Lung Stimulation Asthma  Pneumonia, Pulmonary edema  PE  Liver failure  Sepsis | CNS depression  Opiates  O_2_ in CO_2_ retainer  Neuromuscular  Guillain–Barré  Myasthenia gravis  hypokalemia  Hypophosphatemia  Fatigue  Chest Wall  Kyphosis  Myasthenia crisis  Guillain-Barre  Airway Obstruction Asthma  COPD | GI Losses  Vomiting  NG suction  Renal H+ loss  Diuretics  Bartter’s  Gittelman's  Hyperaldosteronism  Exogenous  Milk alkali syndrome  Volume depletion  Contraction alkalosis | Extra-renal:  Lower GI output  Large volumes of Cl- rich fluids (NS)  Renal causes (RTA’s)  Type I  Type II Type IV  CRI | **Four buckets:**  1. Lactate  2. Ketoacids  3. Ingestions (Osmolar gap)  4. Renal failure  (Dx of exclusion) |

# Anion Gap Mnemonics

| **MUDPILERS** | **GOLDMARK** |
| --- | --- |
| **M**ethanol  **U**remia  **D**KA (or EtOH ketoacidosis)  **P**araldehyde  **I**NH, **I**ron  **L**actate  **E**thanol, **E**th glycol  **R**habdomyolysis  **S**alicylates | **G**lycols (ethylene and propylene)  **O**xyproline  **L**-lactate (not enough oxygen to tissues)  **D**-lactate (albuterol, malignancy, SIBO)  **M**ethanol  **A**spirin  **R**enal failure (from uremia or other organic acid buildup)  **K**etoacidosis (diabetic or EtOH) |

# Lactic acidosis classes

Type A: Hypoperfusion

Type B: No evidence of overt hypoperfusion

- B1: Systemic disease (renal or hepatic failure, diabetes, malignancy)
- B2: Drugs/toxins (albuterol, biguanides, iron, isoniazid, zidovudine, salicylates)
- B3: Inborn errors of metabolism
