# Supplementary material for: Getting up to Speed: A Resident-Led Inpatient Curriculum for New Internal Medicine Interns
Source: MedEdPORTAL. 2019 Dec 27;15:10866. doi: 10.15766/mep_2374-8265.10866 (PMC7012307; doi:10.15766/mep_2374-8265.10866)
Supplement: Supplementary file 1 — A. Intern Survey.docx B. Resident Survey.docx C. Acid-Base Disturbances.docx D. Antibiotics.docx E. Chest Pain.docx F. Safe Discharges.docx G. Gastrointestinal Bleeding and Pancreatitis.docx H. Inpatient Diabetes Management.docx I. Pain Management and Palliative Care.docx J. Shock and Vasopressors.docx [file mep-15-10866-s001.zip › D. Antibiotics.docx]

**Antibiotics**

Intern Guide

**Objectives:**

At the conclusion of this activity, participants will be able to:

1. Identify the clinical features of commonly encountered infectious diseases in hospitalized patients
2. Determine which antibiotic to start empirically in commonly encountered infectious diseases in hospitalized patients
3. Interpret diagnostic tests used in diagnosing infections

**Case 1 (Neutropenic fever)**

Mr. Finn Weeks is a 36M with newly diagnosed AML who is now day 6 of his 7+3 induction chemotherapy regimen. He has had an excellent response to his chemotherapy (which he receives via a double-lumen tunneled Hickman catheter in his right internal jugular vein). You get paged because of a new fever to 101.2 °F. The rest of his vitals are a heart rate of 105, blood pressure 122/78, respiratory rate 18, SpO2 99% on room air. Earlier in the day, your oncology attending was commenting on the fall of his WBC from 30,000 K/uL pre-induction to 2,000 K/uL today. His circulating blasts are undetectable, his PMNs are down to 30%, and his bands are 5%. He has an allergy to penicillin, which caused a rash when he was 5 years old.

**What does this patient have?**

You decide to treat the patient for fever and neutropenia. However, prior to starting antibiotics, you decide to examine the patient first.

**What are you looking for on your exam?**

You don’t find any obvious sources of infection on your exam. You order two sets of blood cultures and a UA/urine culture. (If a cough were present, you would also send a sputum culture and get a chest X-ray.)

**What pathogens concern you the most?**

**Which antibiotic would you like to start in this patient? Does his penicillin allergy affect your choice of antibiotic?**

The next morning, you find your patient has developed a diffuse, macular, erythematous, pruritic rash. You are concerned that cefepime is the culprit, so you want to discontinue this.

**To what antibiotic would you switch?**

The patient continues to have occasional fevers for the next 48 hours. He continues to be without localizing symptoms that would help target antibiotic therapy. The cultures of his blood have no growth to date.

**When would addition of empiric vancomycin for Gram positive coverage be appropriate?**

**When would it be appropriate to discontinue empiric vancomycin coverage?**

Two sets of the patient’s blood cultures start to grow *E. coli* and he is continued on ceftazidime. However, the patient’s rash does not improve after the transition to ceftazidime.

**What is the most likely source? Do you need to worry about source control?**

It is now 4 days after the patient developed a fever and he is currently on piperacillin/tazobactam (Zosyn). The antimicrobial susceptibilities on the original *E. coli* blood culture isolates return with the following:

BLOOD CULTURE

4+ ESCHERICHIA COLI.

| **Antibiotic** | **Result** | **Sensitive (S), Resistant (R), or Intermediate (I)** |
| --- | --- | --- |
| Ampicillin | >= 32 | R |
| Cefazolin | >= 16 | R |
| Cefepime | <= 1 | R |
| Cefotetan | <= 4 | S |
| Ceftazidime | 16 | R |
| Ceftriaxone | <= 1 | R |
| Ciprofloxacin | >= 4 | R |
| Gentamicin | <= 1 | S |
| Imipenem | <= 1 | S |
| Levofloxacin | >= 8 | R |
| Nitrofurantoin | <= 16 | S |
| Trimethoprim/Sulfamethoxazole | <= 20 | S |

**How would you describe this organism? What would you like to do to this patient’s antibiotics?**

**What other antibiotics or other anti-ID agents would you have used if the culture had not returned positive and the patient continued to spike fevers?**

With appropriate antibiotic treatment, the patient improves from his infection. His neutropenia resolves on hospital day 24 and he is discharged. He completes several cycles of consolidation chemotherapy and is in clinical remission.

**How long should the patient be treated for their infection?**

**Case 2 (Pneumonia):**

Ms. Hacks Alotte is a 60F nonsmoker with HTN and HL who presents directly to the ED from clinic, with hypoxemia to 90%, several days of sputum production, and fever to 38 °C (100.4 °F). She lives in a nursing home, where several co-residents have had similar symptoms over the past week.

**What symptoms would you expect for typical vs atypical pneumonia?**

**What would you look for on exam?**

**What diagnostic tests would you send?**

**What would guide your decision on whether to admit or treat as an outpatient?**

**How would procalcitonin affect your decision-making?**

**What pathogens are the most likely culprit?**

**What is your antimicrobial strategy and treatment course?**

**Case 3 (C. diff):**

Mr. Ernie Deruns is a 67M who presented with ACS and underwent emergent CABG with course complicated by VAP treated with vancomycin/levofloxacin. It is now hospital day 23, and you are presenting to the MICU attending when you note his WBC has risen from 7 K/uL to 18 K/uL over the past 24 hours. Vital signs are T 98.5 °F, HR 85, BP 115/50.

# What are potential etiologies for a new leukocytosis?

What diagnostic tests would you send?

What, if any, antibiotics would you start now?

Later that day, the patient develops copious loose stool. The next day (hospital day 24), you review the patient’s microbiology data. All bacterial cultures have no growth to date. A stool C. diff toxin assay is positive. His vital signs are now T 100.3 °F, HR 115, BP 106/40. Stool output over the last 24 hours was 2300 cc. His abdomen is soft, but you notice that he grimaces to deep palpation in the LLQ. There is no rebound tenderness or guarding.

How do you make the diagnosis of C. diff Infection (CDI)?

How would you classify his C. difficile infection (CDI)? What is the next step in management?

Over the next couple of days, the patient’s stool output drops to about 500 cc daily. His vancomycin and levofloxacin are discontinued on hospital day 25. It is now hospital day 26. His vital signs are now T 101.0 °F, HR 120, BP 105/50. WBC is now 24 K/uL. He appears to grimace to light palpation of his abdomen.

What is the appropriate next diagnostic step? Are there any additional therapies that should be initiated?

Later, the nurse asks you to come to the room. You note that the patient now has T 101.6 °F, HR 120, BP 80/40. His abdominal CT scan shows sludge in his gallbladder, colonic wall thickening involving the entire colon, peri-colonic fat stranding around the ascending and descending colon. There is also marked dilation of the sigmoid colon.

How does this change your management?

General Surgery and Infectious Disease feel that a colectomy is indicated if the patient does not improve in the next 24 hours. The patient is no longer having bowel movements, and in fact appears constipated.

What next?

The patient improves over the next 3 days. His temperature drops down to 99.3 °F, HR 105, BP 105/60.

For how long should he be treated for C. diff?

Would you also add probiotics?

**What role does prophylaxis play?**

**References**

1. Kelkar PS, Li JT-C. Cephalosporin Allergy. *N Engl J Med*. 2001;345(11):804-809. doi:10.1056/NEJMra993637

2. Blumenthal KG, Lu N, Zhang Y, Li Y, Walensky RP, Choi HK. Risk of meticillin resistant Staphylococcus aureus and Clostridium difficile in patients with a documented penicillin allergy: Population based matched cohort study. *BMJ*. 2018;361:k2400. doi:10.1136/bmj.k2400

3. Appa AA, Jain R, Rakita RM, Hakimian S, Pottinger PS. Characterizing Cefepime Neurotoxicity: A Systematic Review. *Open Forum Infect Dis*. 2017;4(4):ofx170. doi:10.1093/ofid/ofx170

4. Yahav D, Franceschini E, Koppel F, et al. Seven versus fourteen Days of Antibiotic Therapy for uncomplicated Gram-negative Bacteremia: A Non-inferiority Randomized Controlled Trial. *Clin Infect Dis Off Publ Infect Dis Soc Am*. December 2018. doi:10.1093/cid/ciy1054

5. Chastre J, Wolff M, Fagon J-Y, et al. Comparison of 8 vs 15 days of antibiotic therapy for ventilator-associated pneumonia in adults: A randomized trial. *JAMA*. 2003;290(19):2588-2598. doi:10.1001/jama.290.19.2588

6. Surawicz CM, Brandt LJ, Binion DG, et al. Guidelines for diagnosis, treatment, and prevention of Clostridium difficile infections. *Am J Gastroenterol*. 2013;108(4):478-498; quiz 499. doi:10.1038/ajg.2013.4

7. Hempel S, Newberry SJ, Maher AR, et al. Probiotics for the prevention and treatment of antibiotic-associated diarrhea: A systematic review and meta-analysis. *JAMA*. 2012;307(18):1959-1969. doi:10.1001/jama.2012.3507

8. Van Hise NW, Bryant AM, Hennessey EK, Crannage AJ, Khoury JA, Manian FA. Efficacy of Oral Vancomycin in Preventing Recurrent Clostridium difficile Infection in Patients Treated With Systemic Antimicrobial Agents. *Clin Infect Dis Off Publ Infect Dis Soc Am*. 2016;63(5):651-653. doi:10.1093/cid/ciw401

**Antibiotics**

Instructor Guide

**Objectives:**

At the conclusion of this activity, participants will be able to:

1. Identify the clinical features of commonly encountered infectious diseases in hospitalized patients
2. Determine which antibiotic to start empirically in commonly encountered infectious diseases in hospitalized patients
3. Interpret diagnostic tests used in diagnosing infections

:

**Case 1 (Fever and Neutropenia)**

Mr. Finn Weeks is a 36M with newly diagnosed AML who is now day 6 of his 7+3 induction chemotherapy regimen. He has had an excellent response to his chemotherapy (which he receives via a double-lumen tunneled Hickman catheter in his right internal jugular vein). You get paged because of a new fever to 101.2 °F. The rest of his vitals are a heart rate of 105, blood pressure 122/78, respiratory rate 18, SpO2 99% on room air. Earlier in the day, your oncology attending was commenting on the fall of his WBC from 30,000 K/uL pre-induction to 2,000 K/uL today. His circulating blasts are undetectable, his PMNs are down to 30%, and his bands are 5%. He has an allergy to penicillin, which caused a rash when he was 5 years old.

**What does this patient have?**

- *Definition of fever:*
  - *Single temperature > 101.0°F (>38.3°C)*
  - *Temperature >100.4°F (>38.0°C) for >1h*
- *Definition of neutropenia:*
  - *Absolute Neutrophil Count (ANC) = WBC x (Polys + Bands)*
  - *ANC <500 K/uL (severe) (<1000 K/uL=moderate, <1500K/uL=mild)*
  - *ANC <1000 K/uL with a predicted decrease to < 500 K/uL in the next 48hr*
- *The patient’s ANC is currently 700 K/uL and projected to continue to decline.*

You decide to treat the patient for fever and neutropenia. However, prior to starting antibiotics, you decide to examine the patient first.

**What are you looking for on your exam?**

- *Localizing symptoms, something to target your therapy:*
- *Tunneled line – erythema, tenderness, purulent drainage (note that the absence of these findings does NOT rule out line infection)*
- *Sinuses*
- *Skin – ulcers, cellulitis, peri-anal region (no rectal exam given risk for bacterial translocation)*
- *Oropharynx/dental (mucositis)*
- *Lungs*
- *Abdomen – if abdominal tenderness, would consider CT scan and anaerobic coverage (concern for typhlitis)*

You don’t find any obvious sources of infection on your exam. You order two sets of blood cultures and a UA/urine culture. (If a cough were present, you would also send a sputum culture and get a chest X-ray.)

*If patient has indwelling line, specify “peripheral” and “port/line” when ordered blood cultures orders to keep track of the blood culture results.*

**What pathogens concern you the most?**

- *Gram positive infections (particularly coagulase-negative Staph) are the most common isolated organisms but rarely cause rapid clinical deterioration*
- *Though less common,* ***Gram negative infections****, including Pseudomonas, are the most concerning and must be covered emergently when neutropenic fever develops*
- *If this patient were to die of an overwhelming infection in the next 48 hours, the cause would most likely be a virulent Gram-negative infection*

**Which antibiotic would you like to start in this patient? Does his penicillin allergy affect your choice of antibiotic?**

- *Examine the hospital’s Microbiology Lab Antibiogram for the susceptibility pattern of Pseudomonas found at our institution.*
- *Ideally, you would start an anti-pseudomonal agent, such as ceftazidime or cefepime or piperacillin-tazobactam*
- *If the patient had an IgE-mediated hypersensitivity (anaphylaxis, angioedema) to penicillin, would avoid cephalosporin.*
- *However, if penicillin reaction is rash, there is only a 5-7% cross-reactivity with cephalosporins*^1^*.*
- *In this patient, it is reasonable to start a cephalosporin, being mindful that an allergic reaction is possible. Also consider ordering beta-lactam desensitization pathway, as escalation puts patients at increased risk of MRSA and C difficile*^2^*.*
- *Vancomycin is not indicated in this patient at this time. Treating with vancomycin early can be considered in patients with suspected serious catheter-related infection, evidence of Gram-positive infection, or hemodynamic instability.*

The next morning, you find your patient has developed a diffuse, macular, erythematous, pruritic rash. You are concerned that cefepime is the culprit, so you want to discontinue this.

**To what antibiotic would you switch?**

*Ceftazidime*

- *First Line:*
  - *Cefepime 2g8h*
  - *Ceftazidime 2g q8h - Cross-reactivity between ceftazidime and cefepime is ~1%, so this is a good choice for a presumed cefepime rash*
    - *Also, patients with CNS disturbances after starting cefepime may benefit from switching to ceftazidime due to risk of neurotoxicity.*
    - *Signs of Cefepime neurotoxicity: diminished consciousness, disorientation, agitation, myoclonus*^3^*.*
- *Second Line:*
  - *Piperacillin/tazobactam (Zosyn) 4.5g IV q6h*
- *Third Line:*
  - *Imipenem 500mg IV q6hrs or Meropenem 1 gm q8h – requires ID approval*
  - *Aztreonam 2gm IV q8h + Levofloxacin 500 mg daily – can be considered in patients with cephalosporin allergies. However, there is a high rate of Pseudomonal resistance to these antibiotics at our institution.*
    - *If the patient has recently been on levofloxacin, do not use for neurtropenic fever.*

The patient continues to have occasional fevers for the next 48 hours. He continues to be without localizing symptoms that would help target antibiotic therapy. The cultures of his blood have no growth to date.

**When would addition of empiric vancomycin for Gram positive coverage be appropriate?**

*Empiric vancomycin should be used in febrile neutropenia if:*

- *There is blood culture evidence of Gram-positive infection, but the final identification and sensitivities of the organism are yet unknown.*
- *There is clinical suspicion for catheter-related infections or cellulitis*
- *The patient is hypotensive or thought to be septic.*
- *The patient has a skin or soft tissue infection.*
- *MRSA colonization is NO LONGER considered an indication for empiric vancomycin.*

**When would it be appropriate to discontinue empiric vancomycin coverage?**

*If vancomycin is started empirically and cultures do not show evidence of Gram-positive infection after 48 hours, you could consider discontinuing if patient is otherwise clinically well.*

Two sets of the patient’s blood cultures start to grow *E. coli* and he is continued on ceftazidime.

**What is the most likely source? Do you need to worry about source control?**

*The most likely source is enteric gut translocation from GI mucosal breakdown and impaired healing in the setting of neutropenia, leading to bacteremia. (This is the same reason that neutropenic patients develop mucositis.) The fact that cultures took this long to result is reassuring, as you would expect positive cultures sooner if there was a more virulent source. Given lack of other localizing symptoms or clinical worsening, most likely no need for source control beyond awaiting count recovery, which will promote mucosal healing.*

It is now 4 days after the patient developed a fever and he is currently on Ceftazidime. The antimicrobial susceptibilities on the original *E. coli* blood culture isolates return with the following:

BLOOD CULTURE

4+ ESCHERICHIA COLI.

| **Antibiotic** | **Result** | **Sensitive (S), Resistant (R), or Intermediate (I)** |
| --- | --- | --- |
| Ampicillin | >= 32 | R |
| Cefazolin | >= 16 | R |
| Cefepime | <= 1 | R |
| Cefotetan | <= 4 | S |
| Ceftazidime | 16 | R |
| Ceftriaxone | <= 1 | R |
| Ciprofloxacin | >= 4 | R |
| Gentamicin | <= 1 | S |
| Imipenem | <= 1 | S |
| Levofloxacin | >= 8 | R |
| Nitrofurantoin | <= 16 | S |
| Trimethoprim/Sulfamethoxazole | <= 20 | S |

**How would you describe this organism? What is the next agent that you would switch to for appropriate coverage?**

- *ESBL (Extended Spectrum Beta Lactamase), which is defined as a bacterial species that has resistance to most beta lactams including penicillins, cephalosporins, and aztreonam. Treatment of choice for these patients is to switch to a carbapenem. This bug is also fluoroquinolone-resistant.*
- *Note that the sensitivity table shows that the organism is “sensitive” to cefotetan. This is misleading as ESBL organisms are sensitive to earlier generation cephalosporins in vitro, but not in vivo.*
- *Imipenem 500mg IV q6hrs or Meropenem 1 gm q8h*

**What other antibiotics or other anti-ID agents would you have used if the culture had not returned positive and the patient continued to spike fevers?**

- *Micafungin – Should be added at 100 mg IV daily if patient continues to spike 4-7 days after initiation of antibiotics without known source. Would also start if patient defervesced on antibiotics but then re-spiked, regardless of timeline,. Check a beta-D-glucan and galactomannan when starting and every week thereafter.*
- *If any concern for invasive fungal pneumonia (aspergillus), CONSULT INFECTIOUS DISEASE TEAM and consider initiation of voriconazole as this is the preferred agent.*

With appropriate antibiotic treatment, the patient improves from his infection. His neutropenia resolves on hospital day 24 and he is discharged. He completes several cycles of consolidation chemotherapy and is in clinical remission.

**How long should the patient be treated for their infection?**

- *The patient in this case should continue to be on imipenem for at least 14 days due to his proven bacteremia and neutropenia. He should also remain on imipenem until he is no longer neutropenic.*
- Of note, duration of antibiotics for gram negative bacteremia (without neutropenia) is controversial and shorter antibiotic courses (7 days as opposed to 14 days) have been shown to be effective^4^.
- *In patients where no source is identified, antibiotics should be continued until at least resolution of the fever and bone marrow recovery. This depends largely on attending preference, but typically antibiotics are continued for 48 hours after defervescence and count recovery.*

**Case 2 (Pneumonia):**

Mrs. Hacks Alotte is a 60F nonsmoker with HTN and HL who presents directly to the ED from clinic, with hypoxemia to 90%, several days of sputum production, and fever to 38 °C (100.4 °F). She lives in a nursing home, where several co-residents have had similar symptoms over the past week.

**What symptoms would you expect for typical vs atypical pneumonia?**

*Typical: acute fever, cough, purulent sputum, dyspnea, pleurisy*

*Atypical: insidious dry cough, extrapulmonary symptoms (nausea/vomiting, diarrhea, headaches, myalgias), URI symptoms*

**What would you look for on exam?**

*Lung exam: crackles, egophony, tactile fremitus*

**What diagnostic tests would you send?**

- *Sputum Gram stain/cx: inconsistently revealing; good sample described as <10 sq. cell/HPF. Purulent sputum defined by >25 PMNs/HPF*
- *Blood cx before antibiotics: positive in 10% of inpatients*
- *CXR (PA and lateral): typical=focal consolidation; atypical=interstitial pattern*
- *CBC with diff: leukocytosis +/- bandemia*
- *Procalcitonin*
- *Urine S pneumonia (Se 50-80%, SP>90%), Urine Legionella (only detects L pneumophila L1 serotype, which represents 60-70% of clinical disease)*
- *Other additional studies: Mycoplasma swab for PCR, viral swabs for PCR, HIV, induced sputum for PCP (PJP)/AFB if indicated*

**What would guide your decision on whether to admit or treat as an outpatient?**

*There are various scoring systems available to guide triage. ALL developed for CAP:*

- ***Pneumonia Severity Index (PSI):*** *Estimates mortality for adult patients with community-acquired pneumonia (not developed to predict need for hospitalization)*
  - *age, sex, nursing home resident, neoplastic disease, liver disease, CHF, cerebrovascular disease, renal disease, AMS, RR>29, SBP<90, T<35 or >39.9, pulse>124, pH<7.35, BUN>29mg/dL, Na<130mmol/L, Glc>249mg/dL, Hct<30%, PaO2<60mmHg, Pleural effusion on CXR*
- ***CURB-65****: Estimates mortality of community-acquired pneumonia to help determine inpatient vs. outpatient treatment.*
  - *Confusion, BUN>19mg/dL, RR>=30, SBP<90 or DBP<=60, age>=65*
- ***SMART-COP****: score>=3 points has Se 60-90% and Sp 45-75% for need for ICU care*
  - *SBP<90, Multilobar infiltrates, Alb<3.5, RR>=30, Tachycardia (HR>125), Confusion, O2 sat <90%, arterial pH<7.35*
- ***SCAP****: 1 or more major criteria or 2 or more minor criteria = severe community-acquired pneumonia; patient should be monitored closely (e.g., in an intensive care setting).*
  - *major criteria: pH,7.3, SBP<90; minor criteria: confusion, BUN>30mg/dL, RR>30, multilobar pneumonia on CXR, PaO2<54mmHg or PaO2/FiO2<250, Age>=80*
- *Assess potential barriers to treatment such as vomiting, alcohol/drug use, psychosocial conditions, or cognitive impairments.*

**How would procalcitonin affect your decision-making?**

*Procalcitonin is thought of slightly differently in different settings.*

***Suspected Respiratory Tract Infections:***

- *PCT can be safely used at a cutoff of <0.25 μg/L to withhold initial antibiotic administration in patients with suspected respiratory infections that are* ***stable and low-risk***
- *E.g., minimal oxygen requirement, no hemodynamic compromise or acute organ dysfunction, no severe comorbidities*

***Suggested Interpretation of PCT Values:***

- *<0.1 ng/ml (or decrease from peak value by ≥90%) – Very low likelihood of bacterial infection causing respiratory symptoms (or ongoing infection requiring antibiotics). Withholding (or cessation of antibiotics) strongly encouraged.*
- *0.1-0.24 ng/ml (or decrease from peak value by ≥80%) – Low likelihood of bacterial infection causing respiratory symptoms (or ongoing infection requiring antibiotics). Withholding or cessation of antibiotics encouraged.*
- *≥0.25 ng/ml (and failure to decrease from peak value by ≥80%) – Increased likelihood of bacterial infection*

*********False positives can occur from noninfectious conditions that cause systemic inflammation, such as severe trauma, circulatory shock, major surgery, burns, inhalation injury, and pancreatitis, as well as from certain immunomodulatory agents.*

**What pathogens are the most likely culprit? (No organism ID in 40-60% of cases)**

- ***CAP****: bacterial= S pneumo, Mycoplasma, Chlamydia, H influenza, M catarrhalis (COPD), Legionella (smokers, elderly, immunosuppression), Klebsiella/GNRs (alcoholics, aspiration), S aureus (post viral); viral= influenza A/B*
- ***HAP****: GNRs – Klebsiella, Pseudomonas, E coli, Enterobacter, Serratia, Acinetobacter, S aureus/MRSA*
- ***Immunosuppression****: above + PCP (PJP), fungi, Nocardia, Mycobacteria, CMV, HSV*
- ***Aspiration****: chemical pneumonitis due to aspiration of gastric contents (often sterile); bacterial pneumonia ≥24-72 hours later, due to aspiration of gastric contents 🡪 outpatient=strep, S aureus, anaerobes; inpatient or immunosuppressed =GNR, S aureus*

**What is your antimicrobial strategy and treatment course?**

- ***CAP*** *(5d)-* ***low risk for antibiotic resistance***
  - *[ceftriaxone 1-2g IV Q24 PLUS (azithromycin 500mg PO Q24h or doxycycline 100mg PO BID)]*
    - *OR*
  - *[levofloxacin 750mg PO Q24h monotherapy]*
- ***CAP*** *(5d)-* ***high risk for antibiotic resistance*** *(septic shock, mechanical ventilation, IV antibiotics in last 90 days, cystic fibrosis, bronchiectasis, known colonization with Pseudomonas or MDR pathogen)*
  - *Cefepime 2gm IV Q8hr OR Piperacillin/tazobactam 4.5gm IV Q6h*
    - *PLUS*
  - *Levofloxacin 750mg PO/IV OR azithromycin 500mg PO/IV*
  - *If septic shock, known MRSA colonization, necrotizing pneumonia or new need for mechanical ventilation, add vancomycin 30-45 mg/kg/day IV divided !8h-Q12hr OR linezolid 600mg IV/PO Q12h*
- ***Aspiration*** *(5d)*
  - *ceftriaxone 1-2g IV Q24h*
    - *OR*
  - *levofloxacin 750mg PO Q24h;*
  - *if abscess, add metronidazole 500mg PO Q8h;*
  - *if rapid improvement, consider pneumonitis (see below)*
- ***Aspiration pneumonitis*** *(no antibiotics vs 5d)*
  - *Principles of management – caused by aspiration of sterile gastric contents, often no antibiotics treatment needed within first 48 hrs in healthy young person. Consider treatment per “aspiration” above if:*
    - *Patient at risk for colonization of gastric contents (SBO or on PPI)*
    - *>48hrs of symptoms*
- ***Hospital-Acquired Pneumonia [HAP]*** *(7d)*
  - ***Gram-negative:*** *Cefepime 2gm IV Q8h OR Piperacillin/tazobactam 4.5gm IV Q6h OR Meropenem 1gm IV Q8h*
  - ***Add MRSA agent if septic shock, known MRSA colonization, or new need for mechanical ventilation:*** *Vancomycin 30-45 mg/kg/day IV divided Q8-12hr OR linezolid 600mg IV/PO Q12h*
  - ***If septic shock:*** *add second gram-negative agent, Levofloxacin 750mg IV or gentamicin or tobramycin 7 mg/kg*
- ***Ventilator-Associated Pneumonia [VAP]*** *(7d)*
  - *Cefepime 2gm IV Q8hr OR Piperacillin/tazobactam 4.5gm IV Q6h OR Meropenem 1gm IV Q8h*
    - *PLUS*
  - ***MRSA agent:*** *Vancomycin 30-45 mg/kg/day IV divided Q8-12hr OR Linezolid 600mg IV/PO Q12h*
  - ***If septic shock, add second gram-negative agent:*** *Levofloxacin 750mg IV or gentamicin or tobramycin 7 mg/kg*

**Case 3 (C. diff):**

Mr. Ernie Deruns is a 67M who presented with ACS and underwent emergent CABG with course complicated by VAP treated with vancomycin/levofloxacin. It is now hospital day 23, and you are presenting to the MICU attending when you note his WBC has risen from 7 K/uL to 18 K/uL over the past 24 hours. Vital signs are T 98.5 °F, HR 85, BP 115/50.

# What are potential etiologies for a new leukocytosis?

New rise in WBC raises concern for a new infection, from any source.

- Worsening of his VAP
- Urinary Tract Infection
- Central Line Infection
- C. diff

What diagnostic tests would you send?

- Blood cultures (peripheral x 2)
- Urinalysis/Urine culture
- Endotracheal aspirate culture
- +/- Stool C. diff
- Consider procalcitonin

What, if any, antibiotics would you start now?

If the patient is hemodynamically stable with only a leukocytosis, defer antibiotics until your workup localizes a source for infection.

Later that day, the patient develops copious loose stool. The next day (hospital day 24), you review the patient’s microbiology data. All bacterial cultures have no growth to date. A stool C. diff assay is positive. His vital signs are now T 100.3 °F, HR 115, BP 106/40. Stool output over the last 24 hours was 2300 cc. His abdomen is soft, but you notice that he grimaces to deep palpation in the LLQ. There is no rebound tenderness or guarding.

- In a case like this, starting antibiotics to treat C. diff before the test returned would have been appropriate with his high output stool and rapidly escalating white count

How do you make the diagnosis of C. diff Infection (CDI)?

The diagnosis of CDI is based upon a combination of clinical and laboratory findings:

1. Presence of diarrhea, defined as a passage of 3 or more unformed stools in 24 or fewer consecutive hours (except in cases of severe CDI with ileus)

2. Positive stool testing for C. difficile (antigen/toxin assay), or colonoscopic and/or histopathologic findings demonstrating pseudomembranous colitis

Positive C. difficile 1) Positive toxin A/B assay (enzyme immunoassay (EIA))

assays include: 2) Positive PCR for toxigenic C. difficile (performed upon request if toxin A/B assay negative but glutamate dehydrogenase (GDH) antigen positive and high clinical suspicion of active disease) *

* A positive PCR test does not distinguish between asymptomatic colonization and clinical disease. C. difficile organisms are present as asymptomatic colonizers in up to 10% of hospitalized patients. Treatment is only required for patients with signs of active disease (e.g., diarrhea, fever, leukocytosis, or abdominal pain).

How would you classify his C. difficile infection (CDI)? What is the next step in management?

- Severe Uncomplicated C. difficile Infection
- Vancomycin PO 250mg Q6H
- Consider adding Metronidazole 500 IV Q8H if immunocompromised (can stop once patient is clinically stable x48h)
- Treat with IV fluids, monitor UOP
- Consider ID and surgery consults
- Contact Plus precautions
- Discontinue all other antibiotics as soon as possible (in this case, after 8 days for VAP)^5^
- Stop proton pump inhibitors (PPIs) and H2 antagonists if there is not a strong indication for their use. These medications may increase the risk of C. difficile infection.
- Stop all anti-peristaltic and laxative medications.

Over the next couple of days, the patient’s stool output drops to about 500 cc daily. His IV Vancomycin and Levofloxacin are discontinued on hospital day 25. It is now hospital day 26. His vital signs are now T 101.0 °F, HR 120, BP 105/50. WBC is now 24 K/uL. He appears to grimace to light palpation of his abdomen.

What is the appropriate next diagnostic step? Are there any additional therapies that should be initiated?

- Abdominal CT to look for colitis, toxic megacolon, etc.
- Add Metronidazole 500 mg IV Q8h.
- Why IV and not PO? PO metronidazole is generally better, since the antibiotic is delivered directly to the site of infection. However, in patients with moderate or severe C. diff, there is enough concern for possible colitis-induced ileus, which would result in inadequate drug delivery to the colon. Intravenous metronidazole, furthermore, is excreted in the bile and exuded through the inflamed colon achieving high levels in the intestinal lumen. The same is not true of vancomycin, which must be given orally to impact C. diff. Vancomycin IV does not treat C. diff.

Later, the nurse asks you to come to the room. You note that the patient now has T 101.6 °F, HR 120, BP 80/40. His abdominal CT scan shows sludge in his gallbladder, colonic wall thickening involving the entire colon, peri-colonic fat stranding around the ascending and descending colon. There is also marked dilation of the sigmoid colon.

How does this change your management?

- The patient is now classified as SEVERE COMPLICATED/FULMINANT C. diff because of his temperature and hemodynamic instability.
  - Severe C. diff is considered if WBC > 35K, hemodynamic instability or elevated lactate despite fluid resuscitation, abdominal CT showing severe colitis or megacolon, new ileus or absence of bowel sounds
- Increase Vancomycin to 500mg PO Q6H plus Metronidazole IV Q8H
- Aggressive fluid resuscitation, place catheter to monitor UOP
- Consult General Surgery, GI, and Infectious Disease
  - Surgical indications: worsening clinical picture, peritonitis, perforation with free air, and megacolon
- If Ileus, Consider Vancomycin PR 500mg/100ml Q6H if not contraindicated (neutropenia)
  - Consult general surgery regarding safety of rectal catheter insertion

FMT: Certain patients may be candidates for fecal microbiota transplantation (FMT).

- General Surgery and Infectious Disease feel that a colectomy is indicated if the patient does not improve in the next 24 hours. The patient is no longer having any bowel movements.

What next?

- Vancomycin 500 gram PR q6h for C. diff with ileus, continue IV metronidazole 500mg q8h
- Serial abdominal exams to monitor for peritoneal signs.

The patient improves over the next 3 days. His temperature drops down to 99.3 °F, HR 105, BP 105/60.

For how long should he be treated for C. diff?

- Metronidazole can be stopped once patient clinically stable for 48h
- Consider decreasing PO vancomycin to 125 PO Q6H once clinically stable or 48-72H
- Continue treatment for 10- 14 days (or 5-7 days after completion of other antibiotics)
- If patient has had two or more reoccurrences of C. difficile infection, a vancomycin taper may be instituted and will usually be decided by the ID team
- Fidaxomicin is a new drug with bactericidal activity, kills spores and inhibits production of new spores without destroying normal gut flora. IDSA still with no guidelines for its use but in small studies has shown to be superior to PO vancomycin in C. diff in patients with high risk of relapse. Consult ID or GI for further recs.
- Bezlotoxumab is a monoclonal antibody that binds to C. difficile toxin B and has been show in RCT to lower the rate of recurrence of CDI when given concurrently with metronidazole, vancomycin, or fidaxomicin. It is FDA approved for secondary prevention of CDI in patient at high risk for recurrence. However due to concerns for efficacy and cost-effectiveness it is reserved at our institution for patients who cannot undergo FMT or who have undergone FMT and had a subsequent recurrence of CDI.

Would you also add probiotics?

- *Although the use of probiotics for primary prevention of CDI has not been considered a practice supported by strong evidence in published clinical practice guidelines, there are increasing data to suggest that prophylactic probiotics may protect against CDI in patients at high risk for CDI.*^6,7^
- *Consider ordering Lactobacillus 2 tabs PO TID in hospitalized patients > 50 years old receiving systemic antibiotics, especially with a prior history of CDI.*
- *Do not give live prophylactic probiotics to patients who are severely immunocompromised and/or have an impaired intestinal barrier, because of the risk for translocation and infection.*

**What role does prophylaxis play?**

- *Current national guidelines have found insufficient evidence to either support or refute the practice of using prophylactic PO vancomycin or metronidazole in patients with a history of CDI who require antibiotics for another infectious condition. If opting for this practice, PO vancomycin is the preferred agent (125mg BID). Metronidazole is not recommended for prophylaxis*^8^

**References**

1. Kelkar PS, Li JT-C. Cephalosporin Allergy. *N Engl J Med*. 2001;345(11):804-809. doi:10.1056/NEJMra993637

2. Blumenthal KG, Lu N, Zhang Y, Li Y, Walensky RP, Choi HK. Risk of meticillin resistant Staphylococcus aureus and Clostridium difficile in patients with a documented penicillin allergy: Population based matched cohort study. *BMJ*. 2018;361:k2400. doi:10.1136/bmj.k2400

3. Appa AA, Jain R, Rakita RM, Hakimian S, Pottinger PS. Characterizing Cefepime Neurotoxicity: A Systematic Review. *Open Forum Infect Dis*. 2017;4(4):ofx170. doi:10.1093/ofid/ofx170

4. Yahav D, Franceschini E, Koppel F, et al. Seven versus fourteen Days of Antibiotic Therapy for uncomplicated Gram-negative Bacteremia: A Non-inferiority Randomized Controlled Trial. *Clin Infect Dis Off Publ Infect Dis Soc Am*. December 2018. doi:10.1093/cid/ciy1054

5. Chastre J, Wolff M, Fagon J-Y, et al. Comparison of 8 vs 15 days of antibiotic therapy for ventilator-associated pneumonia in adults: A randomized trial. *JAMA*. 2003;290(19):2588-2598. doi:10.1001/jama.290.19.2588

6. Surawicz CM, Brandt LJ, Binion DG, et al. Guidelines for diagnosis, treatment, and prevention of Clostridium difficile infections. *Am J Gastroenterol*. 2013;108(4):478-498; quiz 499. doi:10.1038/ajg.2013.4

7. Hempel S, Newberry SJ, Maher AR, et al. Probiotics for the prevention and treatment of antibiotic-associated diarrhea: A systematic review and meta-analysis. *JAMA*. 2012;307(18):1959-1969. doi:10.1001/jama.2012.3507

8. Van Hise NW, Bryant AM, Hennessey EK, Crannage AJ, Khoury JA, Manian FA. Efficacy of Oral Vancomycin in Preventing Recurrent Clostridium difficile Infection in Patients Treated With Systemic Antimicrobial Agents. *Clin Infect Dis Off Publ Infect Dis Soc Am*. 2016;63(5):651-653. doi:10.1093/cid/ciw401
