# Supplementary material for: Getting up to Speed: A Resident-Led Inpatient Curriculum for New Internal Medicine Interns
Source: MedEdPORTAL. 2019 Dec 27;15:10866. doi: 10.15766/mep_2374-8265.10866 (PMC7012307; doi:10.15766/mep_2374-8265.10866)
Supplement: Supplementary file 1 — A. Intern Survey.docx B. Resident Survey.docx C. Acid-Base Disturbances.docx D. Antibiotics.docx E. Chest Pain.docx F. Safe Discharges.docx G. Gastrointestinal Bleeding and Pancreatitis.docx H. Inpatient Diabetes Management.docx I. Pain Management and Palliative Care.docx J. Shock and Vasopressors.docx [file mep-15-10866-s001.zip › E. Chest Pain.docx]

Chest Pain

Intern Guide

**Objectives**

At the conclusion of this activity, participants will be able to:

1. Outline a plan for how to respond to a phone call or page about a patient having chest pain
2. Develop a differential diagnosis for chest pain
3. Manage a patient with suspected acute coronary syndrome
4. Identify when and whom to call for help when a patient is having chest pain

**Part 1: TRIAGE**

You are on Cardiology Night Float, and things are finally starting to quiet down around 1AM when you receive the following page*:*

“STAT: x53902 SMITH/Cardiology Ward c/o 8/10 CP, SOB, please come evaluate.”

**How urgently should this page be dealt with? What other information do you want to obtain from the nurse over the phone?**

**PART 2: INFORMATION GATHERING**

As you hurry to the cardiology ward, you look over at the sign-out from your co-intern:

No abx // heparin ppx // Furosemide 80mg BID

75 y/o F hx DM, CAD s/p DES LAD, LCx (2009), CHF, AAA s/p repair, p/w chest pain at rest, resolved on admission, trop neg x 2, ECG baseline, NPO for stress MIBI in AM.

On chart review, you find additional information: LVEF of 45% per last TTE 3 months prior to current admission, +diastolic dysfunction, normal RV function, no significant valvular disease. ECG on admission notable for NSR, RBBB (stable across several EKGs prior to admission). Last cardiac catherization in 2009 notable for 90% LAD s/p DES, 75% LCx s/p DES, diffuse non-flow limiting atherosclerosis throughout.


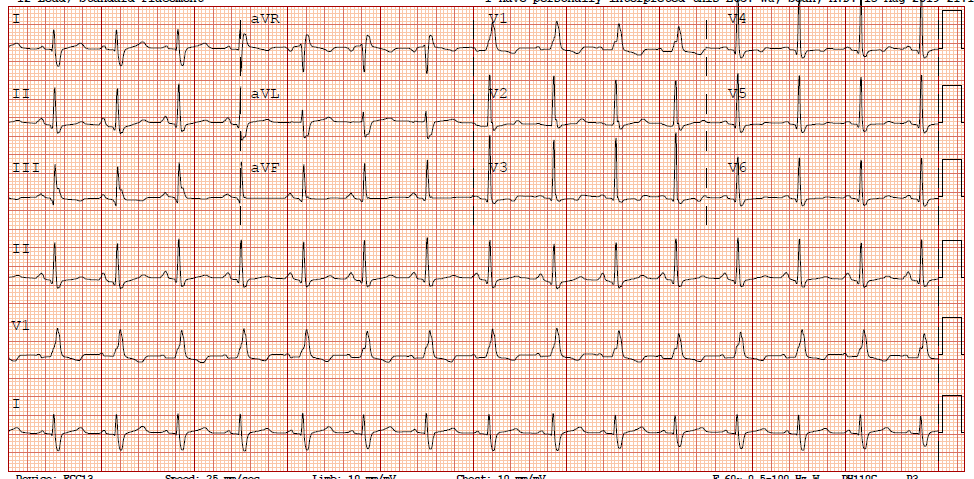


Image owned by authors.

**PART 3: BUILDING A DIFFERENTIAL DIAGNOSIS**

**As you make your way to Ms. Smith’s room, you begin building a differential in your mind. What diagnoses do you never want to miss? What is your differential for acute chest pain?**

**You arrive to Ms. Smith’s bedside and conduct a focused history and physical. What are some key questions that can help you narrow your differential?**

**What are you looking for on exam?**

Once you arrive, Ms. Smith appears uncomfortable and mildly diaphoretic. She complains of persistent substernal “heaviness” that started 15 minutes ago while sitting in a chair watching TV. The “discomfort” feels like the heaviness and shortness of breath she experiences with climbing one flight of stairs, however, this pain hasn’t gone away yet, and normally hers lasts just a few minutes and is then gone. It is 5/10 in severity and is making her SOB and mildly nauseous. No radiation of the pain, no change with deep inspiration or with positioning. Exam is notable for JVP 7cm, clear lungs, regular rate, no murmurs, warm extremities without peripheral edema. You press on her chest and she says, “Ouch! That’s annoying. But that’s not the same as the pressure I’m feeling.”

**PART 3B: NARROWING YOUR DIFFERENTIAL**

**You glance at the 12-Lead ECG and compare it to the admission ECG. What are you looking for?**

First Pass:

Second Pass:

**
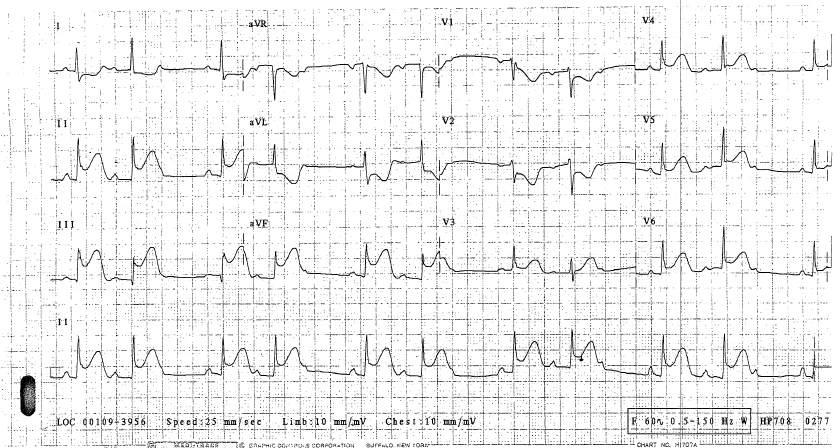
**

Image owned by authors.

**What would prompt you to ask for right-sided leads? Posterior leads?**

Posterior Lead EKG


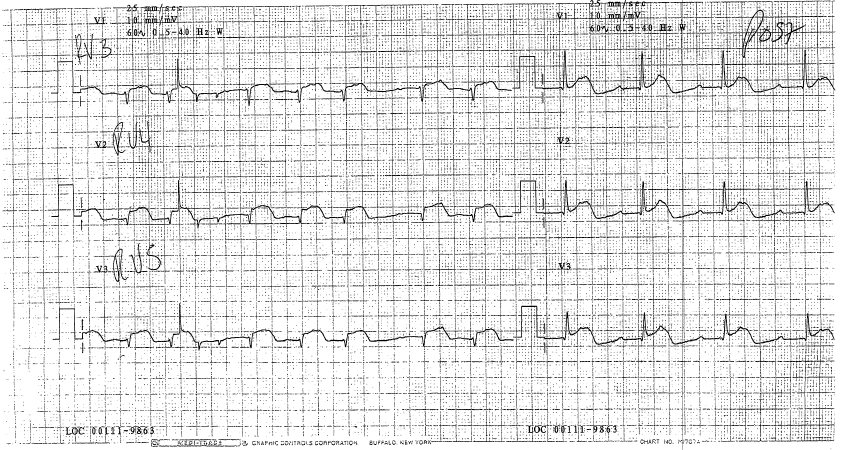


Image owned by authors

Ms. Smith’s ECG reveals a regular rhythm w/ baseline RBBB with new ST depressions in II, III, aVF, no Q waves or evidence of ST elevations. Given these changes, you suspect acute coronary syndrome. You ask the nurse to page your resident.

**PART 4: INITIATING THERAPEUTIC MANAGEMENT**

**Given your suspicion for ACS, what are your next steps in terms of diagnostics? Therapeutics?**

Diagnostics:

Non-medication therapeutics:

Medication therapeutics:

Ms. Smith is now on telemetry with continuous O2 monitoring. Repeat vital signs are T98.6, HR 98, BP 134/78, RR 20, O2 Saturation 94% RA. The IV nurse has placed 2 additional 20-gauge peripheral IV’s and drawn STAT labs. Ms. Smith receives 325mg dose of ASA, 80mg atorvastatin, metoprolol 6.25 q6H, and is started on the nurse-driven heparin protocol with goal PTT 50-70.

Your STAT labs result and are notable for troponin-T 60. After speaking to the cardiology fellow and attending, you and your resident start clopidogrel (loading dose of 300mg per attending followed by 75mg daily), make her NPO after midnight, and order cardiac catherization for the morning.

**Ms. Smith continues to have chest pain. How will you treat her pain?**

After 2 doses of SL nitroglycerin, chest pain is decreased to 3/10 in severity but persistent with BP’s stable at 127/60, HR 70s. You decide to place her on a nitroglycerin drip and repeat an ECG after which she remains chest pain free and her inferior ST depressions resolve. You write a Significant Event note in in the chart.

**If Ms. Smith continued to have ongoing or worsening chest pain despite anti-anginal therapy, what would be the next best step?**

**What will you monitor overnight?**

**References**

1. O’Driscoll BR, Howard LS, Earis J, Mak V. British Thoracic Society Guideline for oxygen use in adults in healthcare and emergency settings. *BMJ Open Respir Res*. 2017;4(1):e000170. doi:10.1136/bmjresp-2016-000170

2. Cohen M, Demers C, Gurfinkel EP, et al. A comparison of low-molecular-weight heparin with unfractionated heparin for unstable coronary artery disease. Efficacy and Safety of Subcutaneous Enoxaparin in Non-Q-Wave Coronary Events Study Group. *N Engl J Med*. 1997;337(7):447-452. doi:10.1056/NEJM199708143370702

3. Antman EM. TIMI 11B. Enoxaparin versus unfractionated heparin for unstable angina or non-Q-wave myocardial infarction: a double-blind, placebo-controlled, parallel-group, multicenter trial. Rationale, study design, and methods. Thrombolysis in Myocardial Infarction (TIMI) 11B Trial Investigators. *Am Heart J*. 1998;135(6 Pt 3 Su):S353-360. doi:10.1016/s0002-8703(98)70265-0

4. Borja J, García O, Donado E, Izquierdo I. Clopidogrel and metoprolol in myocardial infarction. *Lancet Lond Engl*. 2006;367(9513):811-812. doi:10.1016/S0140-6736(06)68327-X

5. Levine GN, Bates ER, Bittl JA, et al. 2016 ACC/AHA Guideline Focused Update on Duration of Dual Antiplatelet Therapy in Patients With Coronary Artery Disease: A Report of the American College of Cardiology/American Heart Association Task Force on Clinical Practice Guidelines: An Update of the 2011 ACCF/AHA/SCAI Guideline for Percutaneous Coronary Intervention, 2011 ACCF/AHA Guideline for Coronary Artery Bypass Graft Surgery, 2012 ACC/AHA/ACP/AATS/PCNA/SCAI/STS Guideline for the Diagnosis and Management of Patients With Stable Ischemic Heart Disease, 2013 ACCF/AHA Guideline for the Management of ST-Elevation Myocardial Infarction, 2014 AHA/ACC Guideline for the Management of Patients With Non-ST-Elevation Acute Coronary Syndromes, and 2014 ACC/AHA Guideline on Perioperative Cardiovascular Evaluation and Management of Patients Undergoing Noncardiac Surgery. *Circulation*. 2016;134(10):e123-155. doi:10.1161/CIR.0000000000000404

6. Meine TJ, Roe MT, Chen AY, et al. Association of intravenous morphine use and outcomes in acute coronary syndromes: Results from the CRUSADE Quality Improvement Initiative. *Am Heart J*. 2005;149(6):1043-1049. doi:10.1016/j.ahj.2005.02.010

7. Parodi Guido, Bellandi Benedetta, Xanthopoulou Ioanna, et al. Morphine Is Associated With a Delayed Activity of Oral Antiplatelet Agents in Patients With ST-Elevation Acute Myocardial Infarction Undergoing Primary Percutaneous Coronary Intervention. *Circ Cardiovasc Interv*. 2015;8(1):e001593. doi:10.1161/CIRCINTERVENTIONS.114.001593

**Chest Pain**

Instructor Guide

# Objectives:

At the conclusion of this activity, participants will be able to:

1. Outline a plan for how to respond to a phone call or page about a patient having chest pain
2. Develop a differential diagnosis for chest pain
3. Manage a patient with suspected acute coronary syndrome
4. Identify when and whom to call for help when a patient is having chest pain

***NB: Italicized text appears only in the Instructor Guide and NOT in the intern guide.***

**Part 1: TRIAGE**

You are on Cardiology Night Float, and things are finally starting to quiet down around 1AM when you receive the following page*:*

“STAT: x53902 SMITH c/o 8/10 CP, SOB, please come evaluate.”

**How urgently should this page be dealt with? What other information do you want to obtain from the nurse over the phone?**

*Triage: This requires urgent evaluation. You’ll call the nurse back while starting to walk over to the room. Information you want includes: brief description of symptoms, full set of vital signs (red flags: tachy/brady, hypoxia, hypotension, extreme hypertension). Vitals should be collected now (don’t rely on set of vitals taken an hour, or even fifteen minutes, ago).*

*Diagnostics: Obtain a full 12-lead EKG. You can ask the nurse to get this while you’re heading to the room.*

*IV access: Ask RN to page IV nurse if inadequate IV access (e.g. fewer than 2 PIVs)*

*Nurse: “Ms. Smith’s most recent VS: T98.7, HR 105, BP 146/80, RR 20, O2 Saturation 94% RA. She is complaining of “chest heaviness” associated with shortness of breath for about 10 minutes now and looks to be in mild distress. I will place her on supplemental O2 and obtain an EKG*.”

**PART 2: INFORMATION GATHERING**

As you hurry to the cardiology ward, you look over at the sign-out from your co-intern:

No abx // heparin ppx // Furosemide 80mg BID

75 y/o F hx DM, CAD s/p DES LAD, LCx (2009), CHF, AAA s/p repair, p/w chest pain at rest, resolved on admission, trop neg x 2, ECG baseline, NPO for stress MIBI in AM.

*Important Sign-Out Details:*

- *IF CHF: LVEF & date of last TTE, etiology of CHF (ischemic, dilated, etc.), +diastolic dysfunction if present, +RV dysfunction or pulmonary hypertension if present, volume status/active diuresis & if c/f CHF decompensation this admission*
- *IF any devices in place (e.g. permanent pacemaker, ICD, etc.)*
- *IF any severe valvular disease (e.g. critical AS)*
- *Contingencies: IF chest pain, then _____; IF hypoxic, ______*
- *History of anginal, atypical or non-anginal CP (e.g. SOB is anginal equivalent for this patient, p/w SOB/nausea at time of last NSTEMI)*
- *Baseline cardiac exam if anything notable (murmurs, S3/S4)*
- *Baseline ECG (this can be reviewed in the chart, but is good to have handy if atypical): LBBB? Baseline ST or T wave changes? Dynamic TWIs on admission which resolved?*

On chart review, you find additional information: LVEF of 45% per last TTE 3 months prior to current admission, +diastolic dysfunction, normal RV function, no significant valvular disease. ECG on admission notable for NSR, RBBB (stable across several EKGs prior to admission (see EKG below). Last cardiac catherization in 2009 notable for 90% LAD s/p DES, 75% LCx s/p DES, diffuse non-flow limiting atherosclerosis throughout.


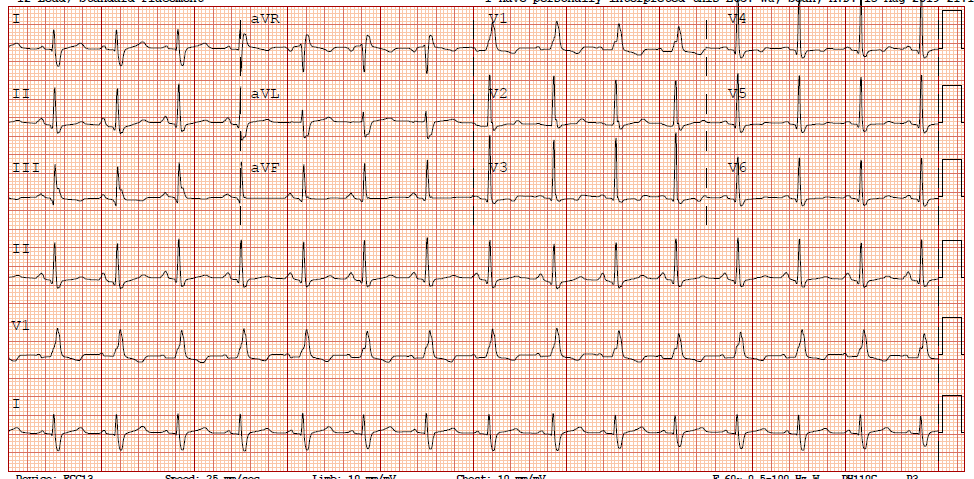


Image owned by authors

**PART 3: BUILDING A DIFFERENTIAL DIAGNOSIS**

**As you make your way to Ms. Smith’s room, you begin building a differential in your mind. What diagnoses do you never want to miss? What is your differential for acute chest pain?**

*Don’t want to miss:*

- *Cardiac: ACS/myocardial infarction, tamponade*
- *Pulmonary: PE, pneumothorax*
- *Vascular: Aortic dissection (especially if HTN)*
- *GI: Esophageal rupture (especially if recent TEE)*

*Broad Differential Diagnosis:*

- *Cardiac: STEMI, ACS, in-stent thrombosis (if recent PCI), stable angina, arrhythmia, coronary vasospasm (drug-induced, including cocaine/amphetamines/chemo, or non-drug induced), demand ischemia, HTN emergency, pericarditis/myocarditis, vasculitis, pheochromocytoma*
- *Pulmonary: PE, pneumothorax, pleuritis, PNA, pulmonary edema (including flash), bronchospasm*
- *Vascular: Acute aortic syndromes*
- *GI: GERD, peptic ulcers, esophageal spasm, esophagitis, pancreatitis, biliary colic*
- *Skin/Chest Wall: Herpes zoster, costochondritis, MSK NOS*
- *Psych: Anxiety, panic attacks*

**You arrive to Ms. Smith’s bedside and conduct a focused history and physical. What are some key questions that can help you narrow your differential?**

- *Now vs prior episodes: comparison to prior chest pain/chronic stable angina*
- *Timing & onset (association with/without activity)*
- *Location & radiation (jaw, arms)*
- *Associated symptoms (nausea, vomiting, SOB, diaphoresis, lightheadedness, syncope)*
- *Quality of pain (diffuse v well localized)*
- *Relief/exacerbating factors (e.g. postprandial, worse with lying down/sitting forward, worse with inspiration, worse with palpation)*

**What are you looking for on exam?**

*General appearance: Altered mental status, respiratory distress, diaphoresis, pale*

*Neck: JVP (elevated in tamponade, HF (including R-sided failure))*

*CV: New murmurs (MR, AR, AS), pericardial rub (pericarditis), irregular rate (arrhythmia)*

*Lungs: Crackles (pulmonary edema), absence of breath sounds (PTX)*

*Ext: Cool (cardiogenic shock), peripheral edema (volume overload), unilateral edema (DVT)*

*Skin/MSK: Reproducible chest wall tenderness/rashes*/*lesions*

*-IF clinical picture concerning for tamponade, should get pulsus as well (pro tip: you can ask an RN to get a Doppler machine, and then use it to help listen for the pulsus if it’s difficult to hear with your stethoscope alone).*

Once you arrive, Ms. Smith appears uncomfortable and mildly diaphoretic. She complains of persistent substernal “heaviness” that started 15 minutes ago while sitting in a chair watching TV. The “discomfort” feels like the heaviness and shortness of breath she experiences with climbing one flight of stairs, however, this pain hasn’t gone away yet, and normally hers lasts just a few minutes and is then gone. It is 5/10 in severity and is making her SOB and mildly nauseous. No radiation of the pain, no change with deep inspiration or with positioning. Exam is notable for JVP 7cm, clear lungs, regular rate, no murmurs, warm extremities without peripheral edema. You press on her chest and she says, “Ouch! That’s annoying. But that’s not the same as the pressure I’m feeling.”

**PART 3B: NARROWING YOUR DIFFERENTIAL**

**You glance at the 12-Lead ECG and compare it to the admission ECG. What are you looking for?**

First Pass *– New ST segment elevations, new LBBB, hyperacute T waves, or new Q waves*

- *If so, page Cardiology fellow STAT for STEMI, discuss activation of Cath lab*

Second Pass *– Go back over it systematically, with focus on the following elements:*

- *PR or ST segment depressions (note: ST depressions/subendocardial ischemia does not localize. If localized/focal, suggestive of reciprocal STEMI & should seek out these changes)*
  - *ST depressions suggestive of ischemia when horizontal or down sloping >= 0.5 mm at the J point in >= 2 contiguous leads*
- *T wave inversions or pseudo-normalization*
  - *T wave inversions suggestive of ischemia are dynamic, >= 1 mm deep & in >= 2 contiguous leads w/ primarily upright QRS/dominant R waves*


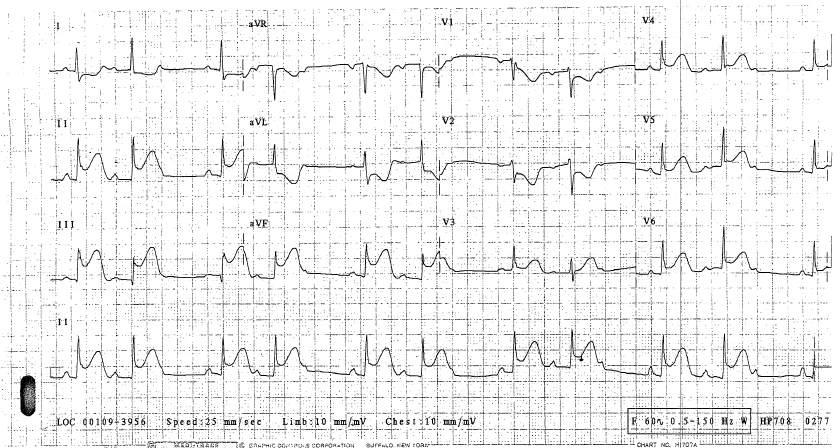


Image owned by authors

- *Loss of R-wave progression*
- *Low voltage*
- *Axis deviation/intervals/conduction blocks/arrhythmias*

*Remember the progression of EKG changes with a STEMI: hyperacute Ts 🡪 ST elevations 🡪 tombstoning 🡪 Q waves with TW inversions 🡪 Q waves with normalized Ts.*

**What would prompt you to ask for right-sided leads? Posterior leads?**

*Right Sided: Evidence of inferior wall ischemia (ST or T wave changes in II, III, or aVF) should prompt you to get V4R, V5R, and V6R to look for RV infarction. Changes in the inferior leads indicate occlusion in the PDA, and since this originates from the RCA in approximately 80% of patients, this may be concerning for right-sided involvement.* ***Think of these leads as dictating a therapeutic decision – if you see ST elevations in the inferior leads, you’ll need to treat a STEMI, but presence of right-sided involvement dictates whether you can use nitrates. If right-sided MI, no nitrates as patient is highly preload-dependent!***

Posterior Lead EKG


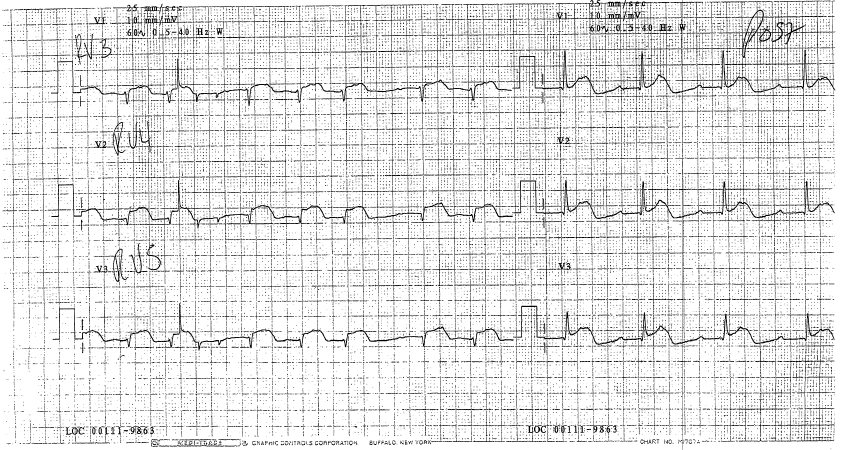


Image owned by authors

*Posterior: Changes in V1-V3 (ST depression, new broad R-waves, new upright T waves) should prompt you to get V7, V8, and V9 to look for posterior infarction; these may be reciprocal changes that you’re seeing.* ***Think of these leads as dictating a diagnostic decision – if you have ST depressions in the anteroseptal leads only, then you have an NSTEMI; if these are indicative of reciprocal ST elevations on a posterior EKG, then you have a STEMI & need to go to Cath lab.***

Ms. Smith’s ECG reveals a regular rhythm w/ baseline RBBB with new ST depressions in II, III, aVF, no Q waves or evidence of ST elevations. Given these changes, you suspect acute coronary syndrome. You ask the nurse to page your resident.

**PART 4: INITIATING THERAPEUTIC MANAGEMENT**

**Given your suspicion for ACS, what are your next steps in terms of diagnostics? Therapeutics?**

Diagnostics*:*

- *Place the patient on telemetry, pulse oximeter, cycle BP q 2-3 minutes*
- *STAT labs: Troponin (would also get CK/CK-MB if recent MI or PCI, and can be helpful to simply draw at the same time to trend), BMP/Mg, PT/INR & PTT, CBC; would get a lactate as well if any concerns re: hypoperfusion/poor forward flow/shock*
  - *When you order troponin, the order should populate as two blood draws, three hours apart*
- *Repeat EKG in ~5 – 10 min, or more frequently based on symptoms and hemodynamics*
- *Consider CXR if concern for pulmonary edema*

*A quick note on high-sensitivity troponin: results are roughly 1000x the values of old assay, though not a direct linear relationship; varies based on the exact values (better calibrated above 4^th^ gen = 0.10 ng/mL == high sensitivity = 100 ng/L). Sex-specific 99^th^ percentile: women = 10 ng/mL, men = 15 ng/L.*

Non-medication therapeutics:

- *Place on supplemental O2 if O2 sat <90% on RA. There is no clear benefit, and possible harm, with supplemental oxygen administration in patients with oxygen saturation greater than 90%*^1^
- *Ask RN to page the IV nurse if IV access not adequate (x2 PIVs)*

Medication therapeutics:

*1) Antiplatelet agents: Give ASA 325mg x 1 (chewed better than swallowed), then 81mg daily*

- *Consider additional agents based on conversations with fellow and attending as choice of agent can be nuanced, and if a patient ends up needing CABG, a washout period of 5-7 days from P2Y12 inhibitors is often required.*
  - *P2Y12 inhibitors: usually clopidogrel, prasugrel, or ticagrelor. Cangrelor is IV and after discontinuation, platelet function normalizes within 1 hour, but used in very select cases due to high expense; this may change with time.*
  - *Glycoprotein IIb/IIIa inhibitors, usually eptifibatide, but abciximab and tirofiban are also in class. Typically initiated in Cath lab for high-risk patients (complex PCI, high clot burden, complications) – not by us.*

*2) Anticoagulation: While enoxaparin was superior in two trials (ESSENCE and TIMI 11B), we often use heparin drip with PTT goal 50-70 (as opposed to goal 60 – 80 for VTE) because it is easily stopped for procedures.*^2,3^

*3) Statin: Atorvastatin 80mg daily (high-intensity statin) for all patients. Studies suggest benefit in early (~12h) initiation, particularly in reducing post-PCI MI, & no harm to starting early.*

*4) Beta blockade: Decreases cardiac demand, reduces mortality through decreased reinfarction and decreased arrhythmia.*

- *Usually PO fractionated metoprolol with goal HR 55-60 (often use SBP < 100 or HR < 55 as holding parameter); can consider IV if no risk factors for shock, or if refractory angina or HTN with ongoing ischemia prior to PCI. COMMIT demonstrated no benefit with IV dosing, and increased rates of cardiogenic shock (but did not exclude patients with evidence of heart failure).*^4^
- *Contraindicated if heart failure, increased risk of shock, low output, or usual BB contraindications*
- *If contraindication to beta blocker, can use nondihydropyridine calcium channel blocker (verapamil or diltiazem).*

*5) ACE inhibitor or ARB*

- *Often not given during first pass of management due to concern for relative or absolute hypotension, or acute kidney injury, but reduced mortality in several trials.*^5^
- *Usually captopril TID or lisinopril daily up titrated to goal BP (often SBP < 110)*
- *Recommended to start within 24h per UpToDate; consistent w/ ACC/AHA STEMI guidelines*^1^

Ms. Smith is now on telemetry with continuous O2 monitoring. Repeat vital signs are T98.6, HR 98, BP 134/78, RR 20, O2 Saturation 94% RA. The IV nurse has placed 2 additional 20-gauge peripheral IV’s and drawn STAT labs. Ms. Smith receives 325mg dose of ASA, 80mg atorvastatin, metoprolol 6.25 q6H, and is started on the nurse-driven heparin protocol with goal PTT 50-70.

Your STAT labs result and are notable for troponin-T 60. After speaking to the cardiology fellow and attending, you and your resident start clopidogrel (loading dose of 300mg per attending followed by 75mg daily), make her NPO after midnight, and order cardiac catherization for the morning.

**Ms. Smith continues to have chest pain. How will you treat her pain?**

*Because anginal chest pain represents active cardiac ischemia, it is important to treat these patients until they are chest pain-free*.

- *SL nitroglycerin 0.4mg tab x 1, q5min up to three times; do not use if PDE inhibitors, RV infarct, severe AS (Can give range from 0.15 – 0.6 mg depending on clinical context, but start with 0.4, and smaller doses can be logistically challenging depending on what’s available on the floor)*
- *If partial pain relief and blood pressure stable, can consider nitroglycerin gtt starting at 5-10 mcg/min with titration to goal chest pain free as BPs tolerate (Again, avoid if: PDE inhibitors, RV infarct, severe AS)*
- *Morphine 1-4mg IV if persistent angina despite therapies above: caution as may simply mask ongoing ischemia and trials associating morphine use with worse outcomes.*^6^ *Morphine is also associated with delayed activity of oral antiplatelet agents.*^7^

*FYI, to take a step back & think about nitro dosing via SL vs gtt:*

- *When we give sublingual nitroglycerin, the doses are much greater over a shorter period, and so BP swings (e.g. resultant hypotension) can be more dramatic.*
- *For example, with a drip at 10 mcg/min, we give 600 mcg/hour, or 0.6 mg over one hour – compared to the 0.4 mg SL that we may repeat three times in 15 minutes!*

After 2 doses of SL nitroglycerin, chest pain is decreased to 3/10 in severity but persistent with BP’s stable at 127/60, HR 70s. You decide to place her on a nitroglycerin drip and repeat an ECG after which she remains chest pain free and her inferior ST depressions resolve. You write a Significant Event note in the chart.

**If Ms. Smith continued to have ongoing or worsening chest pain despite anti-anginal therapy, what would be the next best step?**

*Indications for immediate cardiac catheterization in non-STEMI ACS*

- *Refractory chest pain*
- *Cardiogenic shock/hemodynamic instability*
- *New heart failure or severe LV dysfunction*
- *New or worsening MR, or new VSD*
- *Sustained ventricular arrhythmias*

*If the patient develops any of these signs or symptoms, you should call the cath lab.*

**What will you monitor overnight?**

- *Symptoms: Increase nitroglycerin gtt as BP allows for chest pain. Monitor for symptoms of heart failure.*
- *Vital signs: Up titrate metoprolol with goal HR 55-60 as BP tolerates. Watch for signs of heart failure or shock.*
- *Telemetry and EKGs: Watch for sustained arrhythmias and repeat serial EKGs if any changes in symptoms or monitoring parameters.*
- *Labs: Reorder biomarkers for 3 hours after initial labs drawn (should automatically populate when you order troponin)*
- **If the patient has any of the above signs or symptoms you should call the cath lab urgently overnight**

**References**

1. O’Driscoll BR, Howard LS, Earis J, Mak V. British Thoracic Society Guideline for oxygen use in adults in healthcare and emergency settings. *BMJ Open Respir Res*. 2017;4(1):e000170. doi:10.1136/bmjresp-2016-000170

2. Cohen M, Demers C, Gurfinkel EP, et al. A comparison of low-molecular-weight heparin with unfractionated heparin for unstable coronary artery disease. Efficacy and Safety of Subcutaneous Enoxaparin in Non-Q-Wave Coronary Events Study Group. *N Engl J Med*. 1997;337(7):447-452. doi:10.1056/NEJM199708143370702

3. Antman EM. TIMI 11B. Enoxaparin versus unfractionated heparin for unstable angina or non-Q-wave myocardial infarction: a double-blind, placebo-controlled, parallel-group, multicenter trial. Rationale, study design, and methods. Thrombolysis in Myocardial Infarction (TIMI) 11B Trial Investigators. *Am Heart J*. 1998;135(6 Pt 3 Su):S353-360. doi:10.1016/s0002-8703(98)70265-0

4. Borja J, García O, Donado E, Izquierdo I. Clopidogrel and metoprolol in myocardial infarction. *Lancet Lond Engl*. 2006;367(9513):811-812. doi:10.1016/S0140-6736(06)68327-X

5. Levine GN, Bates ER, Bittl JA, et al. 2016 ACC/AHA Guideline Focused Update on Duration of Dual Antiplatelet Therapy in Patients With Coronary Artery Disease: A Report of the American College of Cardiology/American Heart Association Task Force on Clinical Practice Guidelines: An Update of the 2011 ACCF/AHA/SCAI Guideline for Percutaneous Coronary Intervention, 2011 ACCF/AHA Guideline for Coronary Artery Bypass Graft Surgery, 2012 ACC/AHA/ACP/AATS/PCNA/SCAI/STS Guideline for the Diagnosis and Management of Patients With Stable Ischemic Heart Disease, 2013 ACCF/AHA Guideline for the Management of ST-Elevation Myocardial Infarction, 2014 AHA/ACC Guideline for the Management of Patients With Non-ST-Elevation Acute Coronary Syndromes, and 2014 ACC/AHA Guideline on Perioperative Cardiovascular Evaluation and Management of Patients Undergoing Noncardiac Surgery. *Circulation*. 2016;134(10):e123-155. doi:10.1161/CIR.0000000000000404

6. Meine TJ, Roe MT, Chen AY, et al. Association of intravenous morphine use and outcomes in acute coronary syndromes: Results from the CRUSADE Quality Improvement Initiative. *Am Heart J*. 2005;149(6):1043-1049. doi:10.1016/j.ahj.2005.02.010

7. Parodi Guido, Bellandi Benedetta, Xanthopoulou Ioanna, et al. Morphine Is Associated With a Delayed Activity of Oral Antiplatelet Agents in Patients With ST-Elevation Acute Myocardial Infarction Undergoing Primary Percutaneous Coronary Intervention. *Circ Cardiovasc Interv*. 2015;8(1):e001593. doi:10.1161/CIRCINTERVENTIONS.114.001593
