# Supplementary material for: Getting up to Speed: A Resident-Led Inpatient Curriculum for New Internal Medicine Interns
Source: MedEdPORTAL. 2019 Dec 27;15:10866. doi: 10.15766/mep_2374-8265.10866 (PMC7012307; doi:10.15766/mep_2374-8265.10866)
Supplement: Supplementary file 1 — A. Intern Survey.docx B. Resident Survey.docx C. Acid-Base Disturbances.docx D. Antibiotics.docx E. Chest Pain.docx F. Safe Discharges.docx G. Gastrointestinal Bleeding and Pancreatitis.docx H. Inpatient Diabetes Management.docx I. Pain Management and Palliative Care.docx J. Shock and Vasopressors.docx [file mep-15-10866-s001.zip › F. Safe Discharges.docx]

**Safe Discharges**

Intern Guide

Objectives

At the conclusion of this activity, participants will be able to:

1. Identify the important tasks for discharge and idea timing of these tasks
2. Describe the set of services provided by visiting nurses
3. Discuss specifics of discharge medication reconciliation
4. Describe best practices for communicating with outpatient providers about a patient’s hospitalization

**Case 1**

You are starting a new rotation on the heart failure service tomorrow. Going over your list the night before, you note Mr. Smith is a possible discharge this week. Deciding that this time you are going to be more on top of your discharges than ever before you open the chart…

Mr. Smith is a 69-year-old-man with PMHx of HFpEF, HTN, HLD, T2DM, former smoker, who presented to the hospital with a fall following a syncopal event and was found to have a sub-massive PE, currently undergoing anticoagulation with heparin gtt.

**What things are you thinking about when you read this one-liner? What more do you want to know?**

You find out that Mr. Smith lives at home alone, though his sister lives in an apartment next door and checks on him intermittently. He lives in a second-floor apartment, which he admits has gotten increasingly tough for him to navigate in the recent months.

**Where do you think Mr. Smith might be best served in terms of disposition (what factors would lead to different dispositions)?**

**What if family is adamant that he should go home?**

PT recommends Mr. Smith for short term rehab, so your care coordinator gets to work trying to find him a placement.

**You notice that Mr. Smith is still on a heparin drip “for monitoring”. Does this concern you?**

Your team decides to start Apixaban – a direct oral anticoagulant. You feel that this may be the most convenient option for anticoagulation for Mr. Smith.

**What is your next step?**

You find out that Mr. Smith’s insurance has rejected your request for prior authorization. As an alternative, the team decides to start warfarin. His heparin is continued as a bridge until his INR becomes therapeutic.

**What supports will he require upon discharge given the new warfarin prescription?**

As you present Mr. Smith the following morning your resident asks you if you want to continue Mr. Smith on the 60 of IV Furosemide that he has been on while hospitalized or convert him to his home dose of 20 PO Furosemide daily.

**What more do you want to know?**

You decide to convert him to his home dose of 20mg PO Furosemide, since he is at his estimated dry weight and his exam shows that he is euvolemic.

**How do you go about medication reconciliation? What difficulties have you had in the past?**

It is finally the Day of Discharge. Mr. Smith is so thankful to you for helping him and his family navigate this difficult hospitalization.

**How can you communicate with his outpatient care providers to ensure a smooth transition? What pieces of data will be the most important to include?**

**OPTIONAL ACTIVITY**

Discharging a patient who was admitted for a heart failure exacerbation: :

Ms. Jones is an 88 year old woman with HTN. NIDDM, HFpEF, and atrial fibrillation who was admitted with a heart failure exacerbation that occurred after she went on a family vacation and was eating a higher salt diet than usual. She was diuresed to her dry weight and is not stabilized back on an oral diuretic regimen. You are discharging her home with VNA today.

Turn to your neighbor and together draft the following:

1. Write out your discharge instructions for her VNA
2. List the pieces of information from her hospitalization that you would want to communicate to her outpatient providers. Are there any specific “To-Do” items that you would want to pass along?

**What other things have come up during the discharge process? Can we answer any additional questions?**

Appendix:

| ***Disposition*** | ***Overview*** | ***Requirements*** |
| --- | --- | --- |
| LTAC (Long term acute care facility) | Hospital level care with telemetry, ventilator units, chemo.  Approx. 1 month stay. | -Requires Medicare and 3 nights in ICU  -Medically unstable pts w/ continued needs |
| Acute Rehab | Daily MD monitoring. Should be off 1:1 sitter for 24h prior to transfer. Must do 2.5h PT per day. Approx. 10-14 day stay | -PT/OT eval required  -Complex patients: new stroke, burns, neurologic deficits (rarely medicine pts) |
| SNF (Skilled Nursing Care) | Nursing homes with short term rehab units. Weekly/monthly MD visits. Patients should be off 1:1 sitter 24h prior to transfer. | -PT eval required  -Requires 3-day hospital stay for Medicare patients  -With private insurance can go after 1d |
| Hospice   1. Home hospice 2. SNF with hospice option 3. Hospice residential 4. Inpatient | Care for patients with less than 6 months of  life expectancy. Patients do NOT have to be DNR/DNI. | -6mo life expectancy required |

**VNA/Home with services:**

**Requirements:** “Medical necessity”, *discuss referral with case manager*

**When can they come:** VNA companies assess and decide, services rarely come 7d per week, but can come any day including Sunday, VNA determines length of need from 3 visits to months

**What can they do:** Skilled nursing, wound care, vital signs, lab draws, medication education and monitoring, IV antibiotics, daily weights, CHF programs/monitoring, PT/OT/SLP services, social work, even home health aides to help with physical care

**Case managers can also make referrals to elder services for additional resources**

**Common medications requiring Prior Authorization:**

DOAC, Immunosuppression, tbo-filgrastim, colchicine, Anti-fungals

Any other novel or expensive medication should be checked!

**Referral to Anticoagulation Clinic** *and determine when they will need their next INR drawn, who will draw it, and who will receive the result, and how the patient should expect to receive communication about it.*

**Rehabs and Meds**

- Ensure your patient is discharged with a clear discharge medication list and documentation of when each medication was last administered
- *Do not provide scripts except for controlled substances*

**Caregiver communication**

1. Ensure your patient has all the necessary follow up appointments either scheduled or in process
2. Create brief but informative hospital course in the discharge summary (imagine the data points you might want to know if you were the PCP or the admitting team
3. Clearly communicate important follow up items
4. **For complex patients, consider a warm pass off to PCP via phone or email**

**Safe Discharges**

**Instructor Guide**

Objectives

At the conclusion of this activity, participants will be able to:

1. Identify the important tasks for discharge and idea timing of these tasks
2. Describe the set of services provided by visiting nurses
3. Discuss specifics of discharge medication reconciliation
4. Describe best practices for communicating with outpatient providers about a patient’s hospitalization

**Case 1**

You are starting a new rotation on the heart failure service tomorrow. Going over your list the night before, you note Mr. Smith is a possible discharge this week. Deciding that this time you are going to be more on top of your discharges than ever before you open the chart…

Mr. Smith is a 69-year-old man with PMHx of HFpEF, HTN, HLD, T2DM, former smoker, who presented to the hospital with a fall following a syncopal event and was found to have a sub-massive PE, currently undergoing anticoagulation with heparin gtt.

**What things are you thinking about when you read this one-liner? What more do you want to know?**

- *What is his functional status?*
  - *Does he need a PT Evaluation?*
- *What is his living situation?*
  - *Lives alone or with family?*
  - *How many flights of stairs?*
- *What is the long-term anticoagulation plan?*
  - *Warfarin vs. DOAC?*
- *What medications were started on this admission?*
  - *E.g. heparin drip*
- *What medications were changed on this admission?*
- *What medications need to be switched to home regimen?*
  - *E.g. fractionated vs. long acting, IV vs. PO*
- *What additional resources might this patient require at home?*

You find out that Mr. Smith lives at home alone, though his sister lives in an apartment next door and checks on him intermittently. He lives in a second-floor apartment, which he admits has gotten increasingly tough for him to navigate in the recent months.

**Where do you think Mr. Smith might be best served in terms of disposition (what factors would lead to different dispositions)?**

| ***Disposition*** | ***Overview*** | ***Requirements*** |
| --- | --- | --- |
| *LTAC (Long term acute care facility)* | *Hospital level care with telemetry, ventilator units, chemo.  Approx. 1 month stay.* | *-Requires Medicare and 3 nights in ICU*  *-Medically unstable pts w/ continued needs* |
| *Acute Rehab* | *Daily MD monitoring. Approx. 10-14 day stay*  *Complex patients: new stroke, burns, neurologic deficits (rarely medicine pts)* | *-PT/OT eval required*  *Should be off 1:1 sitter for 24h prior to transfer. Must do 2.5h PT per day.* |
| *SNF (Skilled Nursing Care)* | *Nursing homes with short term rehab units. Weekly/monthly MD visits.* | *-PT eval required*  *-Requires 3-day hospital stay for Medicare patients*  *-With private insurance can go after 1d*  *-Patients should be off 1:1 sitter 24h prior to transfer.* |
| *Hospice*   1. *Home hospice* 2. *SNF with hospice option* 3. *Hospice residential* 4. *Inpatient* | *Care for patients with less than 6 months of*  *life expectancy. Patients do NOT have to be DNR/DNI.* | *-6mo life expectancy required* |

**What if family is adamant that he should go home?**

*VNA/Home with services:*

***Requirements:*** *“Medical necessity”,* *discuss referral with case manager*

***When can they come:*** *VNA companies assess and decide, services rarely come 7d per week, but can come any day including Sunday, VNA determines length of need from 3 visits to months*

***What can they do:*** *Skilled nursing, wound care, vital signs, lab draws, medication education and monitoring, IV antibiotics, daily weights, CHF programs/monitoring, PT/OT/SLP services, social work, even home health aides to help with physical care*

***Case managers can also make referrals to elder services for additional resources***

*Of note, VNA will usually come once per day and not every day. For example, patients must learn/have capacity to administer their own IV antibiotics (patients will be taught and VNA can be present on first administration for guidance/teaching)*

***FYI Patients who are bed-bound can get long-term home services under Medicare***

PT recommends Mr. Smith for short term rehab, so your case manager gets to work trying to find him a placement.

**You notice that Mr. Smith is still on a heparin drip “for monitoring”. Does this concern you?**

*Yes! He can’t go home on a heparin drip.*

Your team decides to start apixaban – a direct oral anticoagulant. You feel that this may be the most convenient option for anticoagulation for Mr. Smith.

**What is your next step?**

*Common medications requiring Prior Authorization:*

*DOAC, sacubitril-valsartan, Immunosuppressive agents, tbo-filgrastim, colchicine, Anti-fungal medications, enoxaparin*

*Any other novel or expensive medication should be checked!*

*Prior Authorization Instructions*

1. *Send script to pharmacy through discharge med rec (EARLY IN ADMISSION!)*
2. *Call pharmacy to find out if medication is covered or requires prior authorization*
3. *If the medication requires a prior authorization, ASK THE PHARMACIST! Often, they can give you a number to call that will expedite the whole process.*
4. *The next step can be extremely variable and depends on patient’s individual insurance and medication! We are working on creating an algorithm with our pharmacists currently.*
5. *Contact the insurance company directly or the number that you receive from the pharmacy for further instructions! Consider asking your pharmacist or case manager if you need help.*

***FYI for some insurances, insulin pens require prior authorization***

***Get this process started early if they may benefit from the pen needle!***

You find out that Mr. Smith’s insurance has rejected your request for prior authorization. As an alternative, the team decides to start warfarin. His heparin will be continued as a bridge until his INR becomes therapeutic.

**What supports will he require upon discharge given the new warfarin prescription?**

*Referral to Anticoagulation Clinic* *and determine when they will need their next INR drawn, who will draw it, and who will receive the result, and how the patient should expect to receive communication about it.*

*****Check with this provider to ensure that they are willing to follow the patient after discharge*****

As you present Mr. Smith the following morning your resident asks you if you want to continue Mr. Smith on the 60 of IV Furosemide that he has been on while hospitalized or convert him to his home dose of 20 PO Furosemide daily.

**What more do you want to know?**

- *Patient’s dry weight (does he have a scale at home?)*
- *Patient’s admission weight*
- *Recent ins and outs*
- *Recent exam findings*
- *Has he been taking his home Furosemide?*
- *Does he have a cardiologist?*

You decide to convert him to his home dose of 20mg PO Furosemide, since he is at his estimated dry weight and his exam shows that he is euvolemic.

**How do you go about medication reconciliation? What difficulties have you had in the past?**

- *Have an accurate preadmission medication list*
  - *Delete any meds patients are NOT taking*
- *Have an accurate list of medications taken at time of discharge*
  - *Ask your resident! Go over discharge med rec together!*
- *Have knowledge of what medication changes were made during hospitalization and the reasons for the changes. As examples:*
  - *Was a proton-pump inhibitor (PPI) initiated for stress ulcer prophylaxis and therefore no longer required, or is ongoing PPI therapy necessary for treatment of an ulcer?*
  - *If a different agent from the same class as one taken prior to admission was substituted during the hospitalization, was there a clinical indication for making that change or was this a therapeutic substitution made based on the hospital’s formulary preference?*
- *Ask if your patient need refills on any meds*
- *Ask if the pharmacy listed in the EMR*
- *Consider if any new medications require prior authorizations*
  - *DOACs, colchicine, BMT meds, antifungals, leucovorin, some antibiotics*
- *Don’t forget that controlled substances require paper scripts*
  - *Opioids, benzodiazepines, dextroamphetamine and amphetamine, other controlled substances*
- ***ALWAYS CHECK state-specific prescription monitoring program for all controlled substances prior to discharge***
- ***Send any patients going home on opioids with script for NASAL NARCAN and associated instructions for use***

*Did you know you can send meds to pharmacy before Date of Discharge?*

*Did you know that scripts are different if patients are going to rehab?*

***Rehab specific Issues:***

- Ensure your patient is discharged with a clear discharge medication list and documentation of when each medication was last administered
- *Do not provide scripts except for controlled substances*
- *Check if your patient needs a MOLST form filled out*

It is finally the Day of Discharge. Mr. Smith is so thankful to you for helping him and his family navigate this difficult hospitalization!

**How can you communicate with his outpatient care providers to ensure a smooth transition? What pieces of data will be the most important to include?**

***Caregiver communication***

1. *Ensure your patient has all the necessary follow up appointments either scheduled or in process*
2. *Create brief but informative hospital course in the discharge summary (imagine the data points you might want to know if you were the PCP or the admitting team*
3. *Clearly communicate important follow up items*
4. ***For complex patients, consider a warm pass off to PCP via phone or email***

**OPTIONAL ACTIVITY**

Discharging a patient who was admitted for a heart failure exacerbation: :

Ms. Jones is an 88 year old woman with HTN. NIDDM, HFpEF, and atrial fibrillation who was admitted with a heart failure exacerbation that occurred after she went on a family vacation and was eating a higher salt diet than usual. She was diuresed to her dry weight and is not stabilized back on an oral diuretic regimen. You are discharging her home with VNA today.

Turn to your neighbor and together draft the following:

1. Write out your discharge instructions for her VNA
2. List the pieces of information from her hospitalization that you would want to communicate to her outpatient providers. Are there any specific “To-Do” items that you would want to pass along?

*Sample Instructions for VNA:*

- *Please obtain daily weight – discharge weight ____*
- *Please educate patient regarding low-salt and fluid-restricted diet*
- *Please help patient with medication management*
- *Discharge medications and doses are as follows: ____*

*Sample list of Information for outpatient providers:*

- *Results from imaging: ****
- *Results from echocardiogram: LVEF = ***%, valve abnormalities = ****
- *Admission BNP = *** and discharge BNP = ****
- *Admission weight = *** and discharge weight = *** (EDW = ***)*
- *Inpatient diuretic regimen, K+ and Mg2+ requirements: ****
- *Discharge diuretic regimen, K+ and Mg2+ requirements: ****
- *Diuretic titration plan (consider home VNA for daily weights): ****
- *Discharge non-diuretic medication regimen: ****
- *Recommended follow-up lab testing (e.g. electrolytes): ****

**What other things have come up during the discharge process? Can we answer any additional questions?**
