# Supplementary material for: Getting up to Speed: A Resident-Led Inpatient Curriculum for New Internal Medicine Interns
Source: MedEdPORTAL. 2019 Dec 27;15:10866. doi: 10.15766/mep_2374-8265.10866 (PMC7012307; doi:10.15766/mep_2374-8265.10866)
Supplement: Supplementary file 1 — A. Intern Survey.docx B. Resident Survey.docx C. Acid-Base Disturbances.docx D. Antibiotics.docx E. Chest Pain.docx F. Safe Discharges.docx G. Gastrointestinal Bleeding and Pancreatitis.docx H. Inpatient Diabetes Management.docx I. Pain Management and Palliative Care.docx J. Shock and Vasopressors.docx [file mep-15-10866-s001.zip › G. Gastrointestinal Bleeding and Pancreatitis.docx]

**Gastrointestinal Bleeding and Pancreatitis**

Intern Guide

Objectives

At the conclusion of this activity, participants will be able to:

1. List the differential diagnosis for epigastric pain
2. Perform risk assessment for a patient with pancreatitis
3. Summarize the management of pancreatitis
4. List the differential diagnosis for upper gastrointestinal bleeding
5. Summarize the management of gastrointestinal bleeding

**Case 1**

You are the admitting intern overnight on ITU. After a somewhat quiet evening, your pager goes off. “ED ADMIT ROSE 1234567 42F w/ epigastric pain”

You begin your chart biopsy only to see that there are no records for this patient. The labs from the ED are also still pending.

You decide to go down to the ED to meet the patient and perform a history and physical.

1. **As you make your way to Ms. Rose’s room, you begin building a differential in your mind. What is your differential for epigastric pain?**
2. **You arrive to Ms. Rose’s bedside to find an uncomfortable 42-year-old female with limited past medical history. What are questions you can ask to help narrow your differential?**

After speaking with Ms. Rose, you learn that she developed acute onset of epigastric pain which radiates to her back earlier this morning. She also tells you that in her past she has been told she may have “biliary colic” but that nothing was ever done about it. You decide to examine her.

1. **What should you pay attention to with regards to her vital signs?**

Her vital signs are **T** 99.9 **HR** 119 **BP** 130/98 **RR** 18 **O2Sat** 98%

1. **As you examine her abdomen/epigastrium, what abdominal/epigastrium exam maneuvers can you utilize to help understand her epigastric pain? What signs may be present on exam?**

Her epigastric/abdominal exam reveals positive bowel sounds, severe tenderness to palpation in the epigastrium, mild tenderness in other quadrants, no distention, no hepatosplenomegaly. The maneuvers and signs you evaluated for are absent.

You decide additional labs and studies will be useful at this time. You walk over to a computer station in the ED to see if her pending labs have come back yet.

1. **What labs would be helpful to you in this case? Would you pursue any imaging?**

Her labs show the following:

**WBC** 13 K/uL **HCT** 43 % (baseline 36 %) **PLTS** 270 K/uL

**Na** 134 mmol/L **K** 3.7 mmol/L **Cl** 90 mmol/L **Co2** 26 mmol/L **BUN** 45 mg/dL **Cr** 1.4 mg/dL (baseline 1.0 mg/dL) **Glu** 103 mg/dL

**Calcium** 8.4 mg/dL

**Lipase** 300 U/L

**ALT** 80 U/L **AST** 78 U/L **AlkP** 130 U/L **Tbili** 3.0 U/L **Dbili** 2.5 U/L

**Triglycerides** 100 mg/dL

Her RUQ shows multiple gallstones within the gallbladder. No stone in the CBD.

You see there is no amylase sent.

1. **What are the differences between checking an amylase and lipase?**
2. **Based on the lab results, you think acute pancreatitis is the highest on your differential. What do you need to make the diagnosis of acute pancreatitis?**
3. **What are the etiologies of acute pancreatitis? Why do you think Ms. Rose has pancreatitis?**
4. **What can you use to help you determine the severity of Ms. Rose’s pancreatitis?**

You decide that Ms. Rose has mild severity pancreatitis. As you continue to think of Ms. Rose’s case, you get another page. “Please enter orders for patient ROSE. Bed open on 10B. Thanks!”

1. **Discuss with a partner how you plan to initiate management with regards to the below issues:**
2. **Fluids**
3. **Pain Control**
4. **Diet**
5. **When is it safe to start feeding patients with mild pancreatitis? When do you need to think about tube feeding?**

You decide to start LR at 250cc/hour, hydromorphone IV 0.4-0.8mg q4hrs prn pain, and to make her NPO.

Your resident is impressed with your critical thinking so far and poses the last question to you.

1. **What complications can occur with acute pancreatitis?**
2. **After Ms. Rose’s acute episode of pancreatitis resolves, what would you do as her outpatient provider to prevent or reduce the likelihood of a recurrent episode?**

**Case 2:**

**Around midnight, after you have tucked the above admission, you receive the following page:**

STAT Pt Smith/10a with large bloody emesis. Please come assess.

You immediately head to assess the patient and, on your way, you review your sign-out:

SMITH 234567890 10a- 14-1 FULL//No antibiotics

60yoM w/ h/o cirrhosis and ETOH abuse and h/o withdrawal seizures who p/w ETOH withdrawal. On standing lorazepam.

You flip through your sign-out to his page and see that he was admitted earlier today, and his notable admission labs were Hct 32 %, Platelets 110 K/uL, INR 2.0,

1. **What are the first things you do as you walk into the patient’s room?**

Vitals: T 97.8 HR 115 BP 90/60 (his systolic blood pressure is usually 100s-110s) RR 16 SpO2 95% on RA. The patient is anxious but mentating well, he denies any abdominal pain. He has two 16 gauge IVs. He does not have a T&S on record.

1. **What do you think might be going on here?**

**Differential of UGIB (most common causes):**

1. **You bring a computer on wheels into the room. What type of information would you want to look up to help you manage the situation?**
2. **Which orders would you like to place at this time? Who would you call?**
3. **What if this patient had no IV access? What type of IV access do you want in a patient with a GI Bleed?**

**References:**

1. Beckingham IJ, Bornman PC. ABC of diseases of liver, pancreas, and biliary system. Acute pancreatitis. *BMJ*. 2001;322(7286):595-598. doi:10.1136/bmj.322.7286.595

2. Raith EP, Udy AA, Bailey M, et al. Prognostic Accuracy of the SOFA Score, SIRS Criteria, and qSOFA Score for In-Hospital Mortality Among Adults With Suspected Infection Admitted to the Intensive Care Unit. *JAMA*. 2017;317(3):290-300. doi:10.1001/jama.2016.20328

3. Brown A, Orav J, Banks PA. Hemoconcentration is an early marker for organ failure and necrotizing pancreatitis. *Pancreas*. 2000;20(4):367-372.

4. Wu BU, Johannes RS, Sun X, Conwell DL, Banks PA. Early changes in blood urea nitrogen predict mortality in acute pancreatitis. *Gastroenterology*. 2009;137(1):129-135. doi:10.1053/j.gastro.2009.03.056

5. Ranson JH, Rifkind KM, Roses DF, Fink SD, Eng K, Spencer FC. Prognostic signs and the role of operative management in acute pancreatitis. *Surg Gynecol Obstet*. 1974;139(1):69-81.

6. Wu BU, Johannes RS, Sun X, Tabak Y, Conwell DL, Banks PA. The early prediction of mortality in acute pancreatitis: A large population-based study. *Gut*. 2008;57(12):1698-1703. doi:10.1136/gut.2008.152702

7. Banks PA, Freeman ML, Practice Parameters Committee of the American College of Gastroenterology. Practice guidelines in acute pancreatitis. *Am J Gastroenterol*. 2006;101(10):2379-2400. doi:10.1111/j.1572-0241.2006.00856.x

8. Wu BU, Hwang JQ, Gardner TH, et al. Lactated Ringer’s solution reduces systemic inflammation compared with saline in patients with acute pancreatitis. *Clin Gastroenterol Hepatol Off Clin Pract J Am Gastroenterol Assoc*. 2011;9(8):710-717.e1. doi:10.1016/j.cgh.2011.04.026

9. Li J, Xue G-J, Liu Y-L, et al. Early oral refeeding wisdom in patients with mild acute pancreatitis. *Pancreas*. 2013;42(1):88-91. doi:10.1097/MPA.0b013e3182575fb5

10. Sachar H, Vaidya K, Laine L. Intermittent vs continuous proton pump inhibitor therapy for high-risk bleeding ulcers: A systematic review and meta-analysis. *JAMA Intern Med*. 2014;174(11):1755-1762. doi:10.1001/jamainternmed.2014.4056

11. Mandorfer M, Bota S, Schwabl P, et al. Nonselective β blockers increase risk for hepatorenal syndrome and death in patients with cirrhosis and spontaneous bacterial peritonitis. *Gastroenterology*. 2014;146(7):1680-1690.e1. doi:10.1053/j.gastro.2014.03.005

**Gastrointestinal Bleeding and Pancreatitis**

**Instructor Guide**

Objectives

At the conclusion of this activity, participants will be able to:

1. List the differential diagnosis for epigastric pain
2. Perform risk assessment for a patient with pancreatitis
3. Summarize the management of pancreatitis
4. List the differential diagnosis for upper gastrointestinal bleeding
5. Summarize the management of gastrointestinal bleeding

**Case 1**

You are the admitting intern overnight. After a somewhat quiet evening, your pager goes off. “ED ADMIT ROSE 1234567 42F w/ epigastric pain”

You begin your chart biopsy only to see that there are no records for this patient. The labs from the ED are also still pending. You decide to go down to the ED to meet the patient and perform a history and physical.

1. **As you make your way to Ms. Rose’s room, you begin building a differential in your mind. What is your differential for epigastric pain?**^20^

*Biliary colic or acute cholecystitis*

*Choledocholithiasis vs. cholangitis*

*Pancreatitis*

*Intestinal obstruction*

*Peptic ulcer disease*

*Hepatitis*

*Ischemic bowel*

*Gastritis*

*Esophagitis*

*GERD*

*IBD*

*IBS*

***Outside the GI system:***

*Renal disease*

*Pulmonary process such as pneumonia or pleurisy*

*Cardiac (ischemia, pericarditis, vasospasms)*

*Aortic Dissection*

*MSK (i.e. costochondritis)*

1. **You arrive to Ms. Rose’s bedside to find an uncomfortable 42-year-old female with limited past medical history. What are questions you can ask to help narrow your differential?**

*Questions should include basic HPI data gathering and relate to differential above but include: nausea, vomiting, relationship to meals, use of NSAIDS/aspirin, GERD symptoms, fevers/chills, h/o gallstones, BMs/flatus, h/o cardiovascular disease, prior history of abdominal surgeries, shortness of breath, pleuritic chest pain, chest discomfort, etc.*

*Red Flag Warnings: Bloody emesis/bowel movements, radiation, sudden onset*

After speaking with Ms. Rose, you learn that she developed acute onset of epigastric pain which radiates to her back earlier this morning. She also tells you that in her past she has been told she may have “biliary colic” but that nothing was ever done about it. You decide to examine her.

1. **What should you pay attention to with regards to her vital signs?**
2. *Signs of systemic illness – SIRS vs qSOFA*^21^

| *SIRS* | *qSOFA* |
| --- | --- |
| *a. Pulse > 90 beats/minute* | *a. Altered Mental Status*  *(GCS <15)* |
| *b. Respirations >20/min or PCO2 < 32 mmHg* | *b. Respiratory Rate >=22* |
| *c. Temperature < 36° C or > 38° C* | *c. SBP=<100* |
| *d. White blood count < 4,000 or >12,000 per mm3* |  |

1. *Signs of hypovolemia – tachycardia and hypotension*
2. *Ensure blood pressures equal and symmetric in bilateral arms (aortic dissection can present with similar clinical symptoms)*

Her vital signs are **T** 99.9 **HR** 119 **BP Right** 130/98 **Left** 127/95 **RR** 18 **O2Sat** 98%

1. **As you examine her abdomen/epigastric area, what exam maneuvers can you utilize to help understand her epigastric pain? What signs may be present on exam?**

***Review the following physical exam signs:***

1. ***Murphy’s sign:*** *Examiner palpates area of the gallbladder while patient deeply inspires. Test is positive if patient has increased pain or suddenly halts their inspiration due to pain. *Tests for cholecystitis*
2. ***Cullen’s sign:*** *Ecchymotic discoloration in the umbilical region * Alerts you to potential retroperitoneal bleeding in the setting of pancreatic necrosis*
3. ***Grey-Turner’s sign:*** *Ecchymotic discoloration in the flanks * Alerts you to potential retroperitoneal bleeding in the setting of pancreatic necrosis*
4. ***Carnett’s sign:*** *Ask the patient to tense their abdominal wall – can either be done by asking patient to do a sit-up or raise legs off table. If pain increases with this maneuver, suggestive of abdominal wall pain as opposed to intra-abdominal pain.*
5. ***Pericarditis:*** *Lean patient forward, pain should improve. Also, can auscultate for friction rub.*

**Note:** Cullen’s and Grey Turner’s signs are only found in about 3% of patients with acute pancreatitis.

Her epigastric/abdominal exam reveals positive bowel sounds, severe tenderness to palpation in the epigastrium, mild tenderness in other quadrants, no distention, no hepatosplenomegaly. The maneuvers and signs you evaluated for are absent.

You decide additional labs and studies will be useful at this time. You walk over to a computer station in the ED to see if her pending labs have come back yet.

1. **What labs would be helpful to you in this case? Would you pursue any imaging?**

***Helpful labs include:*** *CBC, CMP, Lipase, Triglycerides, Calcium*

*If concerned for gallstones as etiology, RUQ ultrasound is useful.*

*CT not necessary for diagnosis as lower sensitivity for cholecystitis and choledocholithiasis but is helpful if cause of abdominal pain is not clear.*

Her labs show the following:

**WBC** 13 K/uL **HCT** 43 % (baseline 36 %) **PLTS** 270 K/uL

**Na** 134 mmol/L **K** 3.7 mmol/L **Cl** 90 mmol/L **Co2** 26 mmol/L **BUN** 45 mg/dL **Cr** 1.4 mg/dL (baseline 1.0 mg/dL) **Glu** 103 mg/dL

**Calcium** 8.4 mg/dL

**Lipase** 300 U/L

**ALT** 80 U/L **AST** 78 U/L **AlkP** 130 U/L **Tbili** 3.0 mg/dL **Dbili** 2.5 mg/dL

**Triglycerides** 100 mg/dL

Her RUQ ultrasound shows multiple gallstones within the gallbladder. No stone in the CBD.

You see there is no amylase sent.

1. **What are the differences between checking an amylase and lipase?**

***Serum amylase levels*** *are less specific and have a short half-life (approximately 10 hours) and normalize within 3-5 days. High amylase levels can also be seen in renal failure, acidosis, cirrhosis, and eating disorders.*

***Lipase*** *is more sensitive and specific, peaks in 24 hours and does not normalize for 8-15 days.*

***Note:*** *Serum amylase levels may be falsely low in alcoholic pancreatitis due to inability of parenchyma to produce amylase and in 50% of pts with hypertriglyceridemia-associated pancreatitis as high TG’s interfere with amylase assay.*

1. **Based on the lab results, you think acute pancreatitis is the highest on your differential. What do you need to make the diagnosis of acute pancreatitis?**

***Need 2 of the 3 below to make a diagnosis:***

1. *Characteristic abdominal pain (epigastric pain, radiating to back, acute)*
2. *Elevation in lipase or amylase > 3 times the upper limit of normal*
3. *Characteristic findings on CT imaging*

***Note:*** *In patients with classic clinical presentation and elevated amylase/lipase, imaging not typically necessary for confirmation*

1. **What are the etiologies of acute pancreatitis? Why do you think Ms. Rose has pancreatitis?**
2. ***Gallstones***
3. ***Alcohol***

*** ***numbers one and two are most common***

*3) Hypertriglyceridemia*

*4) Trauma (Post- ERCP, blunt abdominal injury)*

*5) Hypercalcemia*

*6) Drugs (thiazides, AZT, valproic acid, calcium, estrogen, metronidazole. L-asparaginase, THC)*

*7) Infections (viruses (mumps, coxsackie, HBV, CMV, HSV)*

*8) Vascular (ischemia/vasculitis)*

*9) Genetic (alpha 1 AT deficiency)*

*10) Idiopathic*

1. **What can you use to help you determine the severity of Ms. Rose’s pancreatitis?**

***Certain individual lab factors are helpful:***

1. *HCT “Hemoconcentration with an admission hematocrit > or = 44% and/or failure of admission hematocrit to decrease at approximately 24 hours was associated with the development of necrotizing pancreatitis and organ failure*.”^22^
2. *BUN: “BUN levels were persistently higher among non-survivors than survivors during the first 48 hours of hospitalization”*^23^

*Many scoring tools are available to use. None are perfect, but they are best aid to help the clinician.*

1. *SIRS vs qSOFA – discussed above*
2. *Ranson’s – Increased points, increased mortality. Disadvantage: Need to wait 48 hours.*^24^
3. *BISAP - Increased points, increased mortality (****most commonly used given its ease of use and practicality)***^25^

*BUN > 25 (1 point)*

*Abnormal mental status with a Glasgow coma score <15 (1 point)*

*Evidence of SIRS (1 point)*

*Age > 60 (1 point)*

*Imaging study reveals pleural effusion (1 point)*

1. *APACHE II*^26^

*MAP, HR, RR, A-a gradient or PaO2, pH or HCO3, Na, K, Creatinine, HCT, WBC, Glasgow, Age, Chronic dx*

You decide that Ms. Rose has mild severity pancreatitis. As you continue to think of Ms. Rose’s case, you get another page. “Please enter orders for patient ROSE. Bed open on 10B. Thanks!”

1. **Discuss with a partner how you plan to initiate management with regards to the below issues:**
2. **Fluids**

*Aggressive hydration at 5-10mL/kg/hr with isotonic fluid (NS, LR, can follow BUN, Hct, and UOP to assess adequate response.*

*Evidence shows decreased SIRS response in patients with LR. Do not use LR in hypercalcemia as contains 3mEq/L of calcium!*^27^

1. **Pain Control**

*Patients usually require IV analgesics, especially in light of sensitive GI tract.*

*IV hydromorphone is preferred agent. There is at least a theoretical risk of sphincter of Oddi dysfunction with morphine so can consider it 2^nd^ line.*

1. **Diet**

*NPO for bowel rest.*

1. **When is it safe to start feeding patients with mild pancreatitis? When do you need to think about tube feeding?**

*Oral feeding for mild pancreatitis is safe to start when patients are symptom free. The active desire to eat from patients is also a good indicator. In general, early feeding is preferred and nasojejunal is better than TPN if possible.*^28^

*Enteral feeding is considered in sick patients requiring ICU stay and usually in more severe pancreatitis where feeding cannot be achieved within 5-7 days.* *Enteral nutrition through a nasojejunal tube placed endoscopically or radiologically rather than initiating parenteral nutrition is preferred.*

You decide to start LR at 250cc/hour, hydromorphone IV 0.4-0.8mg q4hrs prn pain, and to make her NPO.

Your resident is impressed with your critical thinking so far and poses the last question to you.

1. **What complications can occur with acute pancreatitis?**

*Local: acute peripancreatic fluid collection, pseudocyst, necrosis (most concerning as has potential to become infected)*

*Also: Splanchnic venous thrombosis, pseudoaneurysm, compartment syndrome*

***Key point: if patients are not improving, need to start investigating for these potential complications.*

1. **After Ms. Rose’s acute episode of pancreatitis resolves, what would you do as her outpatient provider to prevent or reduce the likelihood of a recurrent episode?**

*Cholecystectomy should be performed after recovery in all patients with gallstone pancreatitis. In patients who have had mild pancreatitis, cholecystectomy can usually be performed safely within seven days after recovery and in the same index hospitalization. In cases of severe necrotizing pancreatitis, cholecystectomy should be delayed until active inflammation subsides, and fluid collections resolve or stabilize.*

*Failure to perform a cholecystectomy is associated with a 25 to 30 percent risk of recurrent acute pancreatitis, cholecystitis, or cholangitis within 6 to 18 weeks.*

**Case 2:**

**Around midnight, after you have completed the above admission, you receive the following page:**

STAT Pt Smith with large bloody emesis. Please come assess.

You immediately head to assess the patient and, on your way, you review your sign-out:

**SMITH 234567890 10a- 14-1 FULL//No antibiotics**

60yoM w/ h/o cirrhosis and ETOH abuse and h/o withdrawal seizures who p/w ETOH withdrawal. On standing lorazepam.

You flip through your sign-out to his page and see that he was admitted earlier today, and his notable admission labs were Hct 32 %, Platelets 110 K/uL, INR 2.0 ,

1. **What are the first things you do as you walk into the patient’s room?**

- *Get a fresh set of vitals, examine patient’s abdomen, assess mental status*
- *Have your resident paged*
- *Check to see his IV access*
- *See if he has an active type & screen*

Vitals: T 97.8 HR 115 BP 90/60 (his systolic blood pressure is usually 100s-110s) RR 16 SpO2 95% on RA. The patient is anxious but mentating well, he denies any abdominal pain. He has two 16 gauge IVs. He does not have a T&S on record.

1. **What do you think might be going on here?**

*Most likely- Variceal bleed given h/o cirrhosis but also high on the differential are peptic ulcer and esophageal (Mallory-Weiss) tear.*

**Differential of UGIB (most common causes):**

*-Gastric and/or duodenal ulcers*

*-Esophagitis (infectious vs pill-induced)*

*-Esophagogastric varices with or w/o portal hypertensive gastropathy*

*-Erosive gastritis/duodenitis*

*-Dieulafoy’s lesion*

*-Mallory-Weiss tear*

*-Mass lesions (polyps/cancers)*

*-Angiodysplasia*

1. **You bring a computer on wheels into the room. What type of information would you want to look up to help you manage the situation?**

**-***Prior history of UGIB*

*-Check if known varices/last EGD*

*-Check to see if on SBP or esophageal variceal prophylaxis with nadolol*

1. **Which orders would you like to place at this time?**

***Priorities:***

***1. Hemodynamic resuscitation***

*-Ensure adequate access (at least 2 large bore 18 gauge or larger IV’s, see below)*

***-****IVF: 1L NS bolus STAT*

*-Consent for blood, STAT type and screen if patient does not already have one; consider type & cross*

***2. Airway protection***

*-Consider emergent intubation if patient unable to protect airway 2/2 high volume hematemesis or AMS*

***3. Reversal of coagulopathy***

*-Vitamin K (PO= IV, acts over 24-hour period) as well as 1-unit FFP (immediate-acting, short half-life) to correct coagulopathy*

***4. Pharmacologic Therapy***

*-Octreotide 50mcg bolus followed by 50mcg/hr gtt since you suspect variceal bleed*

*-Pantoprazole IV 80mg x1 stat then pantoprazole 8mg/hr gtt or pantoprazole 40 mg IV BID boluses*

***Note:*** *The data for use of PPI boluses/drips is in* ***peptic ulcer disease*** *not variceal bleed however when etiology of UGIB not know, a high dose bolus should be given. Recent data to suggest no difference between IV PPI boluses BID vs. pantoprazole drip.*^29^

*-Consider erythromycin 3mg/kg IV 30 to 90mins before endoscopy to help clear stomach of blood and improve gastric visualization*

*-Antibiotic- ceftriaxone or quinolone given potential variceal bleed in a cirrhotic puts pt at risk for SBP*

*-Discontinue nadolol (if on it for variceal prophylaxis) not only due to risk of hypotension in setting of Acute GIB but given increased risk of hepatorenal syndrome and death in patients with cirrhosis and SBP.*^30^

*-STAT page GI to consult for emergent upper endoscopy as most variceal bleeds require endoscopic therapy with ligation/banding and/or sclerotherapy*

1. **What if this patient had no IV access? What type of IV access do you want in a patient with a GI Bleed?**

*Page IV nurses to get peripheral IVs. You want at least 18G IVs if not larger. If you are unable to establish peripheral IVs, a cordis would be the next best step.*

*Talk about different kinds of access and flow rates*

**Flow rates of various vascular catheters**

- 20-gauge (.8 mm) x 30 mm angiocath max = 60 cc/ minute
- 18-gauge (1 mm) x 30 mm angiocath max = 105 cc / minute
- 16-gauge (1.3 mm) x 30 mm angiocath max = 220 cc/min
- 14-gauge (2.1mmx83 mm) max = 249 cc/min
- **Triple Lumen Central Line:**
  - medial (blue) & proximal (white) lumen of triple lumen catheter:18-gauge x 190 / 180 mm  max = 26 cc/min
  - distal (brown) lumen of triple lumen catheter:16-gauge x 200 mm max = 52 cc/min
- cordis / introducer: 8.5 French (2.8 mm) x 100 mm max = 126 cc / minute max flow rate with pressure bag @ 300 mmHg: 333 cc / minute
- intraosseous lines: max = 125 cc/minute
- procedural IV:18-gauge x 64 mm angiocath max = 85 cc/min
- PICC 4F single lumen 55cm, 5cc/sec for power injection, 1272cc/hr or 21cc/min
- PICC 6F double lumen 55cm, 5cc/sec for power injection, 753cc/hr or 12cc/min

**References:**

1. Beckingham IJ, Bornman PC. ABC of diseases of liver, pancreas, and biliary system. Acute pancreatitis. *BMJ*. 2001;322(7286):595-598. doi:10.1136/bmj.322.7286.595

2. Raith EP, Udy AA, Bailey M, et al. Prognostic Accuracy of the SOFA Score, SIRS Criteria, and qSOFA Score for In-Hospital Mortality Among Adults With Suspected Infection Admitted to the Intensive Care Unit. *JAMA*. 2017;317(3):290-300. doi:10.1001/jama.2016.20328

3. Brown A, Orav J, Banks PA. Hemoconcentration is an early marker for organ failure and necrotizing pancreatitis. *Pancreas*. 2000;20(4):367-372.

4. Wu BU, Johannes RS, Sun X, Conwell DL, Banks PA. Early changes in blood urea nitrogen predict mortality in acute pancreatitis. *Gastroenterology*. 2009;137(1):129-135. doi:10.1053/j.gastro.2009.03.056

5. Ranson JH, Rifkind KM, Roses DF, Fink SD, Eng K, Spencer FC. Prognostic signs and the role of operative management in acute pancreatitis. *Surg Gynecol Obstet*. 1974;139(1):69-81.

6. Wu BU, Johannes RS, Sun X, Tabak Y, Conwell DL, Banks PA. The early prediction of mortality in acute pancreatitis: A large population-based study. *Gut*. 2008;57(12):1698-1703. doi:10.1136/gut.2008.152702

7. Banks PA, Freeman ML, Practice Parameters Committee of the American College of Gastroenterology. Practice guidelines in acute pancreatitis. *Am J Gastroenterol*. 2006;101(10):2379-2400. doi:10.1111/j.1572-0241.2006.00856.x

8. Wu BU, Hwang JQ, Gardner TH, et al. Lactated Ringer’s solution reduces systemic inflammation compared with saline in patients with acute pancreatitis. *Clin Gastroenterol Hepatol Off Clin Pract J Am Gastroenterol Assoc*. 2011;9(8):710-717.e1. doi:10.1016/j.cgh.2011.04.026

9. Li J, Xue G-J, Liu Y-L, et al. Early oral refeeding wisdom in patients with mild acute pancreatitis. *Pancreas*. 2013;42(1):88-91. doi:10.1097/MPA.0b013e3182575fb5

10. Sachar H, Vaidya K, Laine L. Intermittent vs continuous proton pump inhibitor therapy for high-risk bleeding ulcers: A systematic review and meta-analysis. *JAMA Intern Med*. 2014;174(11):1755-1762. doi:10.1001/jamainternmed.2014.4056

11. Mandorfer M, Bota S, Schwabl P, et al. Nonselective β blockers increase risk for hepatorenal syndrome and death in patients with cirrhosis and spontaneous bacterial peritonitis. *Gastroenterology*. 2014;146(7):1680-1690.e1. doi:10.1053/j.gastro.2014.03.005
