# Supplementary material for: Getting up to Speed: A Resident-Led Inpatient Curriculum for New Internal Medicine Interns
Source: MedEdPORTAL. 2019 Dec 27;15:10866. doi: 10.15766/mep_2374-8265.10866 (PMC7012307; doi:10.15766/mep_2374-8265.10866)
Supplement: Supplementary file 1 — A. Intern Survey.docx B. Resident Survey.docx C. Acid-Base Disturbances.docx D. Antibiotics.docx E. Chest Pain.docx F. Safe Discharges.docx G. Gastrointestinal Bleeding and Pancreatitis.docx H. Inpatient Diabetes Management.docx I. Pain Management and Palliative Care.docx J. Shock and Vasopressors.docx [file mep-15-10866-s001.zip › H. Inpatient Diabetes Management.docx]

Inpatient Diabetes Management

Intern Guide

Objectives

At the conclusion of this activity, participants will be able to:

1. Discuss why oral diabetes medications are often held while patients are hospitalized
2. Describe the pharmacokinetics of the major types of insulin
3. Construct a weight-based insulin regimen for a diabetic patient
4. Propose changes to a hospitalized patient’s insulin regimen based on data from the previous day

**Case 1**

RN is a 50 y/o F with PMH of obesity (Wt.: 80kg), HTN, GERD, and T2DM. Her home diabetes regimen consists of metformin 1000mg BID and glyburide 5mg daily. She is admitted to the hospital with substernal chest pain associated with eating which you suspect is likely secondary to GERD. However, given her CAD risk factors, you make her NPO after midnight for an exercise stress test in the morning.

Given your concerns about using oral agents in this patient’s circumstance, you decide to hold her metformin and glyburide and order an insulin regimen.

**Why do we often hold oral antihyperglycemics like metformin and sulfonylureas and substitute with insulin when patients are admitted to the hospital? Are there other non-insulin agents that we continue in the hospital***?*

**What data to you need to construct an initial insulin regimen for a given patient?**

**What would be a reasonable starting regimen for this patient?**

**What do you do to her regimen given that she will be NPO after midnight?**

She has never used insulin before and has you paged to discuss the order before allowing the floor nurse to give her the insulin. She says “I was told I have mild diabetes. I am afraid that you will drop my sugars by giving me insulin. I think my pancreas is still secreting a lot of insulin on its own.”

**What can you tell her to address her concerns?**

**CASE 2:**

RA is a 64 y/o F with COPD, T2DM admitted with CAP and a COPD flare. At home, she’s on Glargine 70 units nightly and metformin 1,000U BID. Her weight is 88 kg. Her A1C at her last PCP visit was 6.5%. Given her excellent outpatient control, you decide to hold her metformin, continue her home Glargine 70 units nightly plus an aspart SS. You also start her on azithromycin and a 5-day burst of prednisone 60mg QAM.

Her sugars on day 2 are as follows:

7am 48 mg/dL Given orange juice

12noon 442 mg/dL 8 units ss

6pm 442 mg/dL 8 units ss

10pm 204 mg/dL 70 units Glargine

3:45am 45 mg/dL 1-amp D50

What is the problem?

Why is her HbA1c seemingly so well controlled?

How would you adjust her insulin regimen?

It is hospital day 4 and RA is ready for discharge. Her sugars are now well-controlled on Glargine 44U nightly and aspart 14U AC. You are planning to discharge her on a 5-day burst of 40mg of prednisone. She wants to know whether you plan to restart her metformin and for how long she must take the extra insulin with meals.

When should you restart her metformin? When should she stop taking the aspart AC?

CASE 3:

Mr. D is an 82 y/o M with HTN, T2DM, and chronic renal insufficiency who is admitted to your service with a cerebrovascular accident. He was taking metformin and glipizide at the nursing home. He weighs 60kg and has a baseline creatinine of 2.0. During his admission he has a PEG tube placed for enteral access. Nutrition has recommended continuous tube feeds at 60cc/hr. You decide to give Mr. D his home dose of Glargine 30U daily figuring that since he will have continuous feeds, all his insulin can come in a long-acting form.

What is wrong with this logic?

Sure enough, on hospital day 3, the nurse pages you to tell you that Mr. D has a high residual of 400cc, and the RN decides to hold the tube feeds and check the residual q2 hours, with plans to restart when the residual is less than 60cc. (Note, there is no data to support stopping tube feeds for high residuals). The nurse pages you in 2 hours to tell you that the residual is still 200cc but Mr. D’s FSG is now 46. He is still a strict NPO.

How should you alter Mr. D’s insulin regimen to be more tube-feeding friendly?

You now have Mr. D on Glargine 12U + regular insulin 3U q6h + a low-dose regular insulin sliding scale q6h. The nurse pages you again with a residual of 400cc.

What should you do now?

Mr. D is ready to go back to the nursing home, but they request that you change him over to a 12-hour tube feeding cycle, as they do not have the nighttime staff to do tube feeds.

How should you alter Mr. D’s regimen?

Appendix 1: Algorithm for starting insulin in an insulin naive patient

Step One: Identify the daily insulin requirement

Based on the patient’s weight: Daily insulin requirement = 0.4-0.7 U/kg/day

- Reasons for **lower end of the range**: type 1 diabetes, renal insufficiency, small size, recent hypoglycemia, decreasing doses of steroids, older age, insulin-naïve patient
- Reasons for **higher end of the range**: obese, initiation or increasing doses of steroids, marked hyperglycemia

Example for case one: 80kg insulin naive patient x 0.5U/kg/day = 40U/day

Step Two: Divide the daily insulin requirement into basal and nutritional needs

- Basal = 50% of the total daily requirement
- Nutritional requirement = 50% of the total daily requirement (if they are eating)

Example for case one: 40U/day would be 20U basal and 20U nutritional per day

Step Three: Calculate the individual basal doses for either once-daily glargine or twice-daily NPH insulin; calculate the individual prandial doses of aspart

Basal Glargine once daily = same as daily basal dose

Basal NPH twice daily = each dose is 50% of daily basal dose

Prandial Aspart or Lispro dosing = daily nutritional requirement divided by # meals

Example for case one: 20U/day of basal would be 20U glargine QHS or 10U NPH QAM/QHS

20U prandial would be 6-7U aspart AC before each of three meals

Step Four: Choose a supplemental correction scale based on the total amount of scheduled insulin for the day. Same type as nutritional insulin (or regular insulin if NPO or on tube feeds)

# Example for case one: would choose lowest scale (<40units per day scheduled insulin) to start, using aspart QAC (do not use sliding scales at night unless extreme hyperglycemia)

| Example Supplemental/Correction Insulin Scales | | | | |
| --- | --- | --- | --- | --- |
| Blood Glucose | < 40 units/day scheduled insulin | 40-80 units/day scheduled insulin | >80 units/day scheduled insulin | Individualized |
| 150-199 | 1 unit | 1 unit | 2 units | ____units |
| 200-249 | 2 units | 3 units | 4 units | ____units |
| 250-299 | 3 units | 5 units | 7 units | ____units |
| 300-349 | 4 units | 7 units | 10 units | ____units |
| >349 | 5 units + call ho | 8 units + call ho | 12 units + call ho | ___units + call ho |

# Management of DKA and HHS

Objectives

At the conclusion of this activity, participants will be able to:

1. Identify DKA and HHS and distinguish between these two entities
2. Describe the pathophysiology of DKA and HHS
3. **Manage a patient with DKA or HHS**

**Introduction**

This session covers the diagnosis and management of patients with hyperglycemic hyperosmolar state (HHS) and diabetic ketoacidosis (DKA) in the inpatient setting. Patients presenting with these conditions can have severe metabolic derangements and evidence of organ hypoperfusion, with mortality rates ranging from 4-10% and 10-50%, respectively. Early diagnosis and aggressive electrolyte and fluid management are critical.

**Defining DKA and HHS**

States of severe diabetic decompensation in which the absence of insulin (absolute or relative) and the dysregulation of counter-regulatory hormones result in metabolic derangements and severe dehydration.

|  | **DKA** | **HHS** |
| --- | --- | --- |
| **Populations most at risk** | *Type 1 diabetics* | *Type 2 diabetics, especially older individuals with poor renal function* |
| **Rapidity of onset** | *Typically 1-2 days* | *Days to weeks* |
| **Blood glucose** | *< 800 mg/dL (usually 300-500 mg/dL)* | *> 1000 mg/dL (always > 500 mg/dL)* |
| **Arterial pH** | *<7.30* | *>7.30* |
| **Serum HCO3** | *<15 mmol/L (mild cases can be 15-18 mmol/L)* | *>15 mmol/L* |
| **Effective serum osmolarity** | *<320 mosm/L* | *>320 mosm/L* |
| **Serum ketones** | *+++* | *Absent to trace* |
| **Urine ketones** | *+++* | *Absent to trace* |

Effective osmolality = 2[Na] + (BG/18)

(For this calculation use Na from chemistry, not the corrected Na for glucose)

**Pathophysiology Review**

All the clinical and laboratory findings of DKA and HHS can be associated with the absence of insulin and subsequent upregulation of glucagon, catecholamines, and cortisol.

In **DKA**, the absolute absence of insulin results in unopposed activity of glucagon, cortisol, and catecholamines, causing the following:

- Hyperglycemia:
- Increased production: unregulated hepatic gluconeogenesis and glycogenolysis
- Reduced uptake: decreased peripheral glucose uptake
- Ketosis / Acidosis: Increased TG breakdown and transformation into circulating ketones (B-hydroxybutyrate and acetoacetic acid)
- Osmotic diuresis:
- Hyperglycemia => glucosuria and significant dehydration (free water loss), volume contraction, and other electrolyte abnormalities

In contrast, in **HHS**, low insulin levels (relative to counter-regulatory hormones) result in:

- Hyperglycemia:
- Unregulated hepatic gluconeogenesis and glycogenolysis => hyperglycemia (due to glucagon, cortisol, and catecholamines)
- As HHS typically develops over the course of days to weeks, levels of hyperglycemia can reach > 1000
- Osmotic diuresis:
- Significant dehydration due to loss of free water in setting of hyperglycemia
- Electrolyte wasting with significant free water losses

Notably ketosis and significant acidosis are ABSENT in HHS, although the pathophysiology is unclear.

**Question: Given these derangements, what are the key issues to be addressed while managing a patient presenting with either DKA or HHS?**

**CASE 1**

KA is 39M with PMH of type 1 DM. His home diabetes regimen consists of Glargine 20 units at bedtime and aspart insulin 7 units at meal times. He presents to the ED with 2 days of cough, chills, and fatigue.

Triage vital signs are T 101 F, P 98, RR 22, BP 108/60, O2 saturation 93% on RA. Weight is 70kg.

Triage fingerstick glucose is 470mg/dL.

**You are concerned about DKA – what initial tests do you want to order?**

You immediately administer 10 units of aspart insulin and draw stat labs. You also examine him, and you find that he seems very uncomfortable, coughing frequently, breathing rapidly and deeply. He is not using accessory muscles to breathe. Cardiac exam revealed a regular but tachycardic rate; lung exam revealed decreased breath sounds at the right lower lobe with dullness to percussion. Neurologically, the patient was drowsy but arousable, and oriented x3. The results of the lab tests obtained are as follows:

Na 132 mmol/L / K 5 mmol/L / Cl 100 mmol/L / HCO3 12 mmol/L / BUN 36 mg/dL / Cr 1.3 mg/dL (baseline 0.6 mg/dL) / Glu 480 mg/dL

Mg 1.7 mg/dL / PO4 2.4 mg/dL / Ca 8.6 mg/dL

WBC 13 K/uL / Hct 38 mg/dL / Plt 250 K/uL. 85% neutrophils, 1% bands, 13% lymphocytes, 1% monocytes.

EKG: Sinus tachycardia at 102 bpm. Normal axis, no ischemic changes. No peaked T waves.

CXR: Dense RLL consolidation

UA: 2+ ketones, 4+ glucose

Beta-hydroxybutyrate: Pending

ABG: pH 7.18 / pCo2 27 mmHg / paO2 96 mmHg

**What is your interpretation of the data you have collected so far?**

***Diagnostic Criteria for DKA***

*While all categories of DKA have plasma glucose > 250 mg/dL, and urine and serum ketones, several distinctions can be made in the severity of the presentation.*

|  | ***Mild DKA*** | ***Moderate DKA*** | ***Severe DKA*** |
| --- | --- | --- | --- |
| ***Arterial pH*** | *7.25 – 7.30* | *7.00 – 7.24* | *<7.00* |
| ***Serum bicarbonate*** | *15 – 18 mmol/L* | *10 to <15 mmol/L* | *<10 mmol/L* |
| ***Anion gap*** | *>10 mmol/L* | *>12 mmol/L* | *>12 mmol/L* |
| ***Mental status*** | *Alert* | *Alert/drowsy* | *Stupor/coma* |

**How would you manage this patient? What parameters do you need to monitor as you treat the patient?**

**The patient has been initially stabilized; he is on an insulin gtt at 7u / h and is getting IVF. How do you want to monitor this patient?**

**What are the endpoints necessary to determine it is safe to transition the patient from continuous insulin infusion to SQ insulin regimen?**

**How do you transition from IV insulin to SQ insulin?**

**Inpatient Diabetes Management**

**Instructor Guide**

Objectives

At the conclusion of this activity, participants will be able to:

1. Discuss why oral diabetes medications are often held while patients are hospitalized
2. Describe the pharmacokinetics of the major types of insulin
3. Construct a weight-based insulin regimen for a diabetic patient
4. Propose changes to a hospitalized patient’s insulin regimen based on data from the previous day

#### **CASE 1**

RN is a 50 y/o F with PMH of obesity (Wt.: 80kg), HTN, GERD, and T2DM. Her home diabetes regimen consists of metformin 1000mg BID and glyburide 5mg daily. She is admitted to the hospital with substernal chest pain associated with eating which you suspect is likely secondary to GERD. However, given her CAD risk factors, you make her NPO after midnight for an exercise stress test in the morning.

Given your concerns about using oral agents in this patient’s circumstance, you decide to hold her metformin and glyburide and order an insulin regimen.

**Why do we often hold oral antihyperglycemics like metformin and sulfonylureas and substitute with insulin when patients are admitted to the hospital? Are there other non-insulin agents that we continue in the hospital***?*

- *Daily and/or BID dosing of oral medications make them less titratable in inpatients who may have rapidly evolving insulin needs/resistance (infection, eating vs NPO, steroids). The major concern is for prolonged hypoglycemia.*
- *Recommend always stopping sulfonylureas given risk of hypoglycemia. Changes in hemodynamics can also change metabolism of hepatically cleared agents including most sulfonylureas.*

*Metformin should be stopped on admission and evaluated on case by case basis as to when to restart (can consider restarting in someone who is stable, waiting for d/c, no further imaging)*

- *Fluctuating renal function, end-organ perfusion (i.e. sepsis or changes in fluid status secondary to diuresis) and hemodynamics all can change the metabolism of metformin and potentially precipitate lactic acidosis. Additionally, IV contrast is a strict contraindication to continuing metformin as this can also precipitate lactic acidosis.*
- *TZDs (the glitazones) should usually be discontinued in the hospital. This is particularly important in patients with heart failure (can cause peripheral edema which can cloud clinical picture). Long acting meds (take weeks to wear off) so holding them causes little change.*
- *GLP-1 agonists (e.g. liraglutide) and DPP-IV inhibitors (the gliptans) are usually held as they raise risk of post-prandial hypoglycemia which is problematic for patients who may be made NPO or have irregular nutrition in the hospital. These can be easily restarted at discharge.*

**What data to you need to construct an initial insulin regimen for a given patient?**

- *Patient’s weight (kg)*
- *Renal function (insulin is renally cleared)*
- *Influential factors affecting insulin dosing: new medication (i.e. steroids), dietary status (NPO, tube feeds), acute illness (i.e. infection)*
- *Useful starting guidelines for starting insulin:*
  - *Insulin naive patients:* ***0.5 units/kg/day (total daily dose)***
  - *Insulin resistant patients:* ***0.7 units/kg/day (total daily dose)***
- *T2DM and obese patients typically need higher doses given high insulin resistance compared to thin or T1DM patients*

**What would be a reasonable starting regimen for this patient?**

1. *Identify daily insulin requirement* (80kg patient at 0.5U/kg/day= 40 units/day)
2. *Divide the daily insulin requirement into basal and nutritional needs* (20 units basal + 20 units nutritional)
3. *Calculate individual basal doses for either once daily glargine or twice daily NPH* (20 units glargine or 10 units NPH BID, with 7 units aspart with meals)
   1. *When starting basal insulin, glargine or NPH is appropriate, but NPH’s BID dosing makes it more rapidly titratable though it has a different peak which needs to be kept in mind.*
4. *Choose a supplemental correctional scale of short acting insulin (aspart QAC if eating –with QHS if significant insulin resistance -- or regular insulin Q6h if NPO or on tube feeds. Regular insulin has less peak than aspart and is therefore the insulin of choice in patients with continuous tube feeds patients that are NPO). Choose your sliding scale based on the total daily dose (TDD):*

*If TDD < 40 units/day- low dose correction scale*

*If TDD 41-80/day- use moderate*

- 1. *If > 81 should be calculating custom scale*

**What do you do to her regimen given that she will be NPO after midnight?**

*Stop meal time, continue sliding scale and basal. In general, basal insulin should not change depending on patient’s nutritional status. NPH has a small peak effect and limited post-prandial coverage, as a result, can at times be reduced in times of fasting.*

***Guidelines:***

1. *If a patient is on glargine, make no changes if a patient is NPO. You can do a weight-based double check before ordering (in general the TDD should often not exceed 1 unit/ kg unless known insulin resistance).*
2. *If a patient is on NPH QAM/QHS, make no changes to the QHS NPH (after all, they’re fasting each night)*
3. *If the AM NPH dose is higher than the HS NPH dose, then at least cut the AM dose to be the same as the QHS dose*
4. *For the AM NPH, if a patient’s fasting sugars are high that morning (e.g., > 180 mg/dL), make no additional changes to the NPH dose. If the sugars are normal, consider cutting that morning’s NPH by 25-50%.*

***Suggested adjustment in this case:***

1. *Give 20 units of glargine or if on NPH, give 10 units NPH QPM the night before*
2. *Check fasting FS In AM, if >180mg/dl, give usual 10U NPH QAM, if <180mg/dL, give 5 units instead*

She has never used insulin before and has you paged to discuss the order before allowing the floor nurse to give her the insulin. She says “I was told I have mild diabetes. I am afraid that you will drop my sugars by giving me insulin. I think my pancreas is still secreting a lot of insulin on its own.”

**What can you tell her to address her concerns?**

- *She is correct that as a person living with type 2 diabetes, her problem is more one of insulin resistance and less about insulin secretion. However, you can reassure her that native hormonal regulatory mechanisms will cause her own pancreas to reduce its insulin production if she were to approach hypoglycemia.*
- *Additionally, you can reassure her that her you will be starting with a low dose of insulin (less than her own body would make), that she will have her finger stick glucose frequently monitored, and that you will promptly adjust the insulin regimen as needed.*

*Lastly, just because she is getting insulin in the hospital does not mean (and probably won’t mean) that she will go home on insulin*. *It is often helpful to remind patients that you are using the insulin to replace their outpatient medications (so for this patient, you could consider telling them you’re using basal bolus to replace the metformin and sulfonylurea).*

**CASE 2:**

RA is a 64 y/o F with COPD, T2DM admitted with CAP and a COPD flare. At home, she’s on Glargine 70 units nightly and metformin 1,000 mg BID. Her weight is 88 kg. Her A1C at her last PCP visit was 6.5%. Given her excellent outpatient control, you decide to hold her metformin, continue her home Glargine 70 units nightly plus an aspart SS. You also start her on azithromycin and a 5-day burst of prednisone 60mg QAM.

Her sugars on day 2 are as follows:

7am 48 mg/dL Given orange juice

12noon 442 mg/dL 8 units ss

6pm 442 mg/dL 8 units ss

10pm 204 mg/dL 70 units Glargine

3:45am 45 mg/dL 1-amp D50

What is the problem?

- The primary problem is that her regimen only consists of basal insulin (receiving almost all her total daily dose (TDD) as basal insulin only (70 units glargine, weights 88kg)
- Her basal Glargine dosing is too high as evidenced by hypoglycemia throughout the night and early morning
- Steroids increase prandial insulin requirements >> basal requirements
- Anticipate for steroids to cause more pronounced post-prandial hyperglycemia, which worsens throughout the day. We often use NPH or longer acting agents to combat this rise.

Why is her HbA1c seemingly so well controlled?

- Her HbA1c reflects average normoglycemia despite episodes of hypo- and hyperglycemia. Other possibilities for a false negative HgbA1c include conditions that lead to high RBC turnover, such as hemolytic anemia or treatment for iron, B12, or folate deficiencies.

How would you adjust her insulin regimen?

Suggested Approach in Patients Received Steroids:

1. Increase prandial/basal insulin ratio
2. Administer NPH insulin in conjunction with the prednisone administration so the steroid and insulin peak will better correspond to one another (the peak in NPH matches the anticipated glucose rise from prednisone).

Suggest Approach to Case:

1. Start with her home dose = total daily dose = 70 units/day = 35 units NPH BID, 12 U aspart qAC
2. Increase prandial/nutritional requirement by 20% to 14U qAC since holding her oral anti-hyperglycemics and administering steroids
3. Decrease her NPH by ~20% to account for her hypoglycemic episodes in the AM. This can then be increased based on subsequent fasting sugars

It is hospital day 4 and RA is ready for discharge. Her sugars are now well-controlled on Glargine 44U nightly and aspart 14U AC. You are planning to discharge her on a 5-day burst of 40mg of prednisone. She wants to know whether you plan to restart her metformin and for how long she must take the extra insulin with meals.

When should you restart her metformin? When should she stop taking the aspart AC?

- Restart metformin on discharge after you evaluated the eGFR, and stop aspart AC (both standing and sliding scale). Metformin should not be prescribed in patients with GFR < 30 cc/hr.
- Discharge patient home on lower dose of glargine, perhaps 60 units to prevent hypoglycemia (especially if adding back metformin)
- Avoid prescribing insulin taper as steroids are discontinued as too complicated and potentially dangerous
- Closely monitor FS post-discharge (VNA, close follow up, self-monitoring FS qAC if patient able

CASE 3:

Mr. D is an 82 y/o M with HTN, T2DM, and chronic renal insufficiency who is admitted to your service with a cerebrovascular accident. He was taking metformin and glipizide prior to admission. He weighs 60kg and has a baseline creatinine of 2.0. During his admission he has a PEG tube placed for enteral access. Nutrition has recommended continuous tube feeds at 60cc/hr. You decide to give Mr. D his home dose of Glargine 30U daily figuring that since he will have continuous feeds, all his insulin can come in a long-acting form.

What is wrong with this logic?

- A total daily dose of 0.5U/kg/day may be too much in this thin, insulin-naive patient who also has compromised renal function
- If the tube feeds are held or decreased for any reason, then he may have too much insulin on board as basal (equivalent to not holding the AC insulin for a patient who is NPO).

Sure enough, on hospital day 3, the nurse pages you to tell you that Mr. D has a high residual of 400cc, and the RN decides to hold the tube feeds and check the residual q2 hours, with plans to restart when the residual is less than 60cc. (Note, there is no data to support stopping tube feeds for high residuals). The nurse pages you in 2 hours to tell you that the residual is still 200cc but DM’s FSG is now 46. He is still a strict NPO.

How should you alter Mr. D’s insulin regimen to be more tube-feeding friendly?

Suggested Insulin Regimen for Continuous Tube Feeds:

1. Calculate total daily dose (lower to 0.4units/kg/day in this case (40 units/day) given CKD, low weight, age)
2. Divide into basal and prandial (12 units of glargine QD or NPH 6 U BID + regular insulin q6h for continuous tube feeds (in this case 3U q6h)
3. Regular insulin has less peak than aspart and is therefore the insulin of choice in patients with continuous tube feeds and patients that are NPO

You now have Mr. D on Glargine 12U + regular insulin 3U q6h + a low-dose regular insulin sliding scale q6h. The nurse pages you again with a residual of 400cc.

What should you do now?

- Hold regular insulin and keep the basal insulin and sliding scale
- Check FS q1-2hr
- If patient is hypoglycemic or regular insulin recently administered, can consider D10 at TF rate until last dose wears off or residuals decrease, and TF are resumed

Mr. D is ready to go back to the nursing home, but they request that you change him over to a 12-hour tube feeding cycle, as they do not have the nighttime staff to do tube feeds.

How should you alter Mr. D’s regimen?

***Suggested Approach to the Case:***

- *In cycled tube feeds, nutritional insulin should be given during the period tube feeds are running. No changes made to basal insulin; continue 12 units glargine daily*
- *The 12 units of nutritional insulin (regular) should be given while the tube feeds are running only*
- *Since TF are only running for 12 hours and regular insulin is administered 6 hours apart, must divide total prandial dose into two (12/2=6): Can give 6 units of regular insulin at the start of tube feeds (e.g. 0600) and 6 again in 6 hours (e.g. 1200) for a total of 12 units*

Appendix 1: Algorithm for starting insulin in an insulin naive patient

Step One: Identify the daily insulin requirement

Based on the patient’s weight: Daily insulin requirement = 0.4-0.7 U/kg/day

- Reasons for **lower end of the range**: type 1 diabetes, renal insufficiency, small size, recent hypoglycemia, decreasing doses of steroids, older age, insulin-naïve patient
- Reasons for **higher end of the range**: obese, initiation or increasing doses of steroids, marked hyperglycemia

Example for case one: 80kg insulin naive patient x 0.5U/kg/day = 40U/day

Step Two: Divide the daily insulin requirement into basal and nutritional needs

- Basal = 50% of the total daily requirement
- Nutritional requirement = 50% of the total daily requirement (if they are eating)

Example for case one: 40U/day would be 20U basal and 20U nutritional per day

Step Three: Calculate the individual basal doses for either once-daily glargine or twice-daily NPH insulin; calculate the individual prandial doses of aspart

Basal Glargine once daily = same as daily basal dose

Basal NPH twice daily = each dose is 50% of daily basal dose

Prandial aspart or dosing = daily nutritional requirement divided by # meals

Example for case one: 20U/day of basal would be 20U glargine QHS or 10U NPH QAM/QHS

20U prandial would be 6-7U aspart AC before each of three meals

Step Four: Choose a supplemental correction scale based on the total amount of scheduled insulin for the day. Same type as nutritional insulin (or regular insulin if NPO or on tube feeds)

# Example for case one: would choose lowest scale (<40units per day scheduled insulin) to start, using aspart QAC (do not use sliding scales at night unless extreme hyperglycemia)

| Example Supplemental/Correction Insulin Scales | | | | |
| --- | --- | --- | --- | --- |
| Blood Glucose | < 40 units day scheduled insulin | 40-80 units day scheduled insulin | >80 units day scheduled insulin | Individualized |
| 150-199 | 1 unit | 1 unit | 2 units | ____units |
| 200-249 | 2 units | 3 units | 4 units | ____units |
| 250-299 | 3 units | 5 units | 7 units | ____units |
| 300-349 | 4 units | 7 units | 10 units | ____units |
| >349 | 5 units + call ho | 8 units + call ho | 12 units + call ho | ___units + call ho |

# Management of DKA and HHS

Objectives

At the conclusion of this activity, participants will be able to:

1. Identify DKA and HHS and distinguish between these two entities
2. Describe the pathophysiology of DKA and HHS
3. Manage a patient with DKA or HHS

**Introduction**

This session covers the diagnosis and management of patients with hyperglycemic hyperosmolar state (HHS) and diabetic ketoacidosis (DKA) in the inpatient setting. Patients presenting with these conditions can have severe metabolic derangements and evidence of organ hypoperfusion, with mortality rates ranging from 4-10% and 10-50%, respectively. Early diagnosis and aggressive electrolyte and fluid management are critical.

**Objectives**

- Briefly review the clinical presentation of DKA and HHS
- Review the pathophysiology and examine the implications for management in DKA and HHS
- Practice initial management of DKA
- Practice initial management of HHS

**Defining DKA and HHS**

States of severe diabetic decompensation in which the absence of insulin (absolute or relative) and the dysregulation of counter-regulatory hormones result in metabolic derangements and severe dehydration.

|  | **DKA** | **HHS** |
| --- | --- | --- |
| **Populations most at risk** | *Type 1 diabetics* | *Type 2 diabetics, especially older individuals with poor renal function* |
| **Rapidity of onset** | *Typically, 1-2 days* | *Days to weeks* |
| **Blood glucose** | *< 800 mg/dL (usually 300-500 mg/dL)* | *> 1000 mg/dL (always > 500 mg/dL)* |
| **Arterial pH** | *<7.30* | *>7.30* |
| **Serum HCO3** | *<15 mmol/L (mild cases can be 15-18 mmol/L)* | *>15 mmol/L* |
| **Effective serum osmolarity** | *<320 mosm/kg* | *>320 mosm/Kg* |
| **Serum ketones** | *+++* | *Absent to trace* |
| **Urine ketones** | *+++* | *Absent to trace* |

Effective osmolality = 2[Na] + (BG/18)

(For this calculation use Na from chemistry, not the corrected Na for glucose)

**Pathophysiology Review**

All the clinical and laboratory findings of DKA and HHS can be associated with the absence of insulin and subsequent upregulation of glucagon, catecholamines, and cortisol.

In **DKA**, the absolute absence of insulin results in unopposed activity of glucagon, cortisol, and catecholamines, causing the following:

- Hyperglycemia:
- Increased production: unregulated hepatic gluconeogenesis and glycogenolysis
- Reduced uptake: decreased peripheral glucose uptake
- Ketosis / Acidosis: Increased TG breakdown and transformation into circulating ketones (B-hydroxybutyrate and acetoacetic acid)
- Osmotic diuresis:
- Hyperglycemia => glucosuria and significant dehydration (free water loss), volume contraction, and other electrolyte abnormalities

In contrast, in **HHS**, low insulin levels (relative to counter-regulatory hormones) result in:

- Hyperglycemia:
- Unregulated hepatic gluconeogenesis and glycogenolysis => hyperglycemia (due to glucagon, cortisol, and catecholamines)
- As HHS typically develops over the course of days to weeks, levels of hyperglycemia can reach > 1000
- Osmotic diuresis:
- Significant dehydration due to loss of free water in setting of hyperglycemia
- Electrolyte wasting with significant free water losses

Notably ketosis and significant acidosis are ABSENT in HHS, although the pathophysiology is unclear.

**Question: Given these derangements, what are the key issues to be addressed while managing a patient presenting with either DKA or HHS?**

- *Identifying the trigger / precipitant.*
- *Infection (20-25%)*
- *Medication non-compliance*
- *Other causes: MI, CVA, mesenteric ischemia, pancreatitis, steroids, other medications*
- *Fluid balance and rehydration.*
- *DKA typically 5-7L of fluid down*
- *HHS typically 7-12L of fluid down*
- *Achieving rapid glucose control.*
- *Initially with IV or SQ form, then safely transitioning to SQ regimen for home*
- *Monitoring electrolytes and acid-base status closely and aggressively repleting.*
- *Sodium: levels variable, as patients may have low or normal levels, depending upon the severity preceding osmotic diuresis (loss of free water, with variable loss of Na) and ongoing hyperglycemia (will pull free water into serum, leading to hyponatremia)*
- *Potassium: Total body depletion due to osmotic diuresis, though levels may be normal due to extracellular shift in setting of hyperglycemia and acidosis.*
- *Phosphorus, calcium, and magnesium levels should be monitored.*
  - *Although hypophosphatemia is common, aggressive repletion should not be pursued unless PO4 < 1.0 mg/dL*
  - *Calcium and magnesium levels should be repleted*

**Review the DKA and HHS algorithms, pointing out that they are the keys to management**

**CASE 1**

KA is 39M with PMH of type 1 DM. His home diabetes regimen consists of Glargine 20 units at bedtime and aspart insulin 7 units at meal times. He presents to the ED with 2 days of cough, chills, and fatigue.

Triage vital signs are T 101 F, P 98, RR 22, BP 108/60, O2 saturation 93% on RA. Weight is 70kg.

Triage fingerstick glucose is 470 mg/dL.

**You are concerned about DKA – what initial tests do you want to order?**

- *Basic metabolic panel, lactate*
- *CBC with differential*
- *Beta- hydroxybutyrate*
- *CXR*
- *Plasma osmolality (if you are concerned about HHS, although less likely in this patient)*
- *Urinalysis and sediment (for ketones, e/o infection)*
- *Blood cultures and urine culture if UA c/f infection*
- *Electrocardiogram*
- *Sputum cultures*

*In addition, in women of reproductive age, it is important to check a B-hCG, as DKA or HHS in the setting of pregnancy will require careful, aggressive management.*

You immediately administer 10 units of aspart insulin and draw stat labs. You also examine him, and you find that he seems very uncomfortable, coughing frequently, breathing rapidly and deeply. He is not using accessory muscles to breathe. Cardiac exam revealed a regular but tachycardic rate; lung exam revealed decreased breath sounds at the right lower lobe with dullness to percussion. Neurologically, the patient was drowsy but arousable, and oriented x3. The results of the lab tests obtained are as follows:

Na 132 mmol/L / K 5 mmol/L / Cl 100 mmol/L / HCO3 12 mmol/L / BUN 36 mg/dL / Cr 1.3 mg/dL (baseline 0.6 mg/dL) / Glu 480 mg/dL

Mg 1.7 mg/dL / PO4 2.4 mg/dL / Ca 8.6 mg/dL

WBC 13 K/uL / Hct 38 % / Plt 250 K/uL. 85% neutrophils, 1% bands, 13% lymphocytes, 1% monocytes.

EKG: Sinus tachycardia at 102 bpm. Normal axis, no ischemic changes. No peaked T waves.

CXR: Dense RLL consolidation

UA: 2+ ketones, 4+ glucose

Beta-hydroxybutyrate: Pending

ABG: pH 7.18 / pCO2 27 mmHg / paO2 96 mmHg

**What is your interpretation of the data you have collected so far?**

*DKA: elevated anion gap (20) acidosis, hyperglycemia and ketonemia (DKA), likely secondary to RLL PNA.*

*Dehydration: elevated Cr and BUN:Cr ratio. Potassium levels are top-normal, but other electrolytes show evidence of depletion in setting of osmotic diuresis.*

*This patient has moderate DKA, precipitated by acute infection (i.e. pneumonia).*

***Diagnostic Criteria for DKA***

*While all categories of DKA have plasma glucose > 250 mg/dL, and urine and serum ketones, several distinctions can be made in the severity of the presentation.*

|  | ***Mild DKA*** | ***Moderate DKA*** | ***Severe DKA*** |
| --- | --- | --- | --- |
| ***Arterial pH*** | *7.25 – 7.30* | *7.00 – 7.24* | *<7.00* |
| ***Serum bicarbonate*** | *15 – 18 mmol/L* | *10 to <15 mmol/L* | *<10 mmol/L* |
| ***Anion gap*** | *>10 mmol/L* | *>12 mmol/L* | *>12 mmol/L* |
| ***Mental status*** | *Alert* | *Alert/drowsy* | *Stupor/coma* |

**How would you manage this patient? What parameters do you need to monitor as you treat the patient?**

*Use the ADA DKA management algorithm to run through the following:*

*Key aspects of initial DKA management include IV fluid repletion, insulin administration, and electrolyte management.*

- *Rehydration. Patients are typically 5-7L fluid deplete due to osmotic diuresis.*
  1. *Rate of repletion dependent upon hydration status and Na*
  2. *Need to correct Na for hyperglycemia*
     1. *Quick rule of thumb: Add 1.6 mmol/L to the measured Na for every 100 that glucose is ABOVE 100. So, in our patient, Na corrects to 138 mmol/L)*
  3. *Add glucose to fluids when BG < 250 mg/dL*
- *Glucose management.*
  1. *Begin with IV bolus (0.10-0.15U/kg), followed by continuous infusion at 0.10U/kg/h.*
     1. *IF serum glucose does not fall by 50-70 mg/dL if first hour, double dose*
  2. *Check q1h FSBG while on insulin gtt.*
  3. *In cases where patients are being managed on the floor with DKA, if nursing will not allow insulin gtt, can use q2h boluses of Aspart*
  4. *Again, once BG < 250 mg/dL, add glucose to fluids*
- *Potassium management.*
  1. *If K < 3.3 mmol/L, do not give insulin, as this may precipitate hypokalemia (due to intracellular shift in response to insulin). Replete K recheck level and then start insulin*
  2. *If K is between 3.3 mmol/L and 5.3 mmol/L, add K to IVF*
  3. *If K is > 5.3 mmol/L, no need for K repletion at this time*
- *Treat underlying pathophysiology. Antibiotics for CAP in this case*

**The patient has been initially stabilized; he is on an insulin gtt at 7u / h and is getting IVF. How do you want to monitor this patient?**

*Ideally will follow K, HCO3, AG, and ABG every 2-4 hours. FSBG should be checked hourly for insulin gtt titration. Ideally, patients with significant acidosis and AG should be admitted to MICU instead of floor, given the intensity of monitoring required.*

*On the floor, lab draws can be obtained q4-6h, and FSBG can be checked q4h maximum.*

**What are the endpoints necessary to determine it is safe to transition the patient from continuous insulin infusion to SQ insulin regimen?**

*Glucose <200-250 mg/dL, pH > 7.3, HCO3 > 18 mmol/L, corrected AG <12 mmol/L, ability to eat*

**How do you transition from IV insulin to SQ insulin?**

- *Overlap SQ insulin with IV for 2h*
- *For the transition from IV to SQ in hyperglycemic crisis, often need to use weight-based estimate to anticipate decreased requirements the subsequent day as their acute crisis resolves*
- *For patients newly being started on insulin: SQ insulin regimen should include basal (typically NPH initially) + aspart dosing based on patient weight + ISS*
- *For patients previously on stable home insulin regimen: Can restart home regimen with ISS coverage.*

*Calculations for Case 1 patient:

Weight: 70kg TDD: 0.4-0.6U/kg

*Use lower end of scale for Type 1 Diabetics due to increased sensitivity.

TDD = 70 x 0.4 = 28

50% Basal = 14U 🡪 NPH 7U QAM/ 7U QPM

50% meal time: Aspart 4-5U QAC

Low dose Aspart sliding scale
