# Supplementary material for: Getting up to Speed: A Resident-Led Inpatient Curriculum for New Internal Medicine Interns
Source: MedEdPORTAL. 2019 Dec 27;15:10866. doi: 10.15766/mep_2374-8265.10866 (PMC7012307; doi:10.15766/mep_2374-8265.10866)
Supplement: Supplementary file 1 — A. Intern Survey.docx B. Resident Survey.docx C. Acid-Base Disturbances.docx D. Antibiotics.docx E. Chest Pain.docx F. Safe Discharges.docx G. Gastrointestinal Bleeding and Pancreatitis.docx H. Inpatient Diabetes Management.docx I. Pain Management and Palliative Care.docx J. Shock and Vasopressors.docx [file mep-15-10866-s001.zip › I. Pain Management and Palliative Care.docx]

# Pain Management and Palliative Care

Intern Guide

Objectives

At the conclusion of this activity, participants will be able to:

1. Describe multiple approaches to discussing code status at the time of hospital admission
2. Demonstrate an approach to initiating and up-titrating pain and nausea medications in the inpatient setting
3. Practice conversations about serious illness and end of life issues
4. Identify and treat common symptoms at the end of life

**Part 1: Code status**

You are on your first night of oncology night float, and you get your first admission. Mr. L is a 75M with metastatic prostate CA who is complaining of severe back pain. He has known spinal metastases. His physical exam and MRI rule out cord compression but do show new compression fractures at L4/L5. You finish your H&P and are about to head upstairs when you get the following page.

PAGE: “Don’t forget to confirm code status!” -NF resident

**How do you approach code status in someone you just met?**

1. Normalize:

2. Explain why:

3. Ask/assess*:*

4. Reflect/Summarize:

5. Document the order.

**PART 2: PAIN MANAGEMENT**

When you get back to the workroom, you get another page…

PAGE: “Please call: Mr. L/703 back pain. Needs hydromorphone.” - RN

When we think about pain control, we often talk about oral & topical therapies; when/how to use these is dictated by the type of pain & its location.

**What are the differences between neuropathic & nociceptive pain, and what types of therapies do we use for each?**

**What type of pain does Mr. L have?**

**If his pain is mild, which PRN agents could we use?**

**What topical or anti-spasmodic adjuvants could we use to supplement?**

**If he has moderate pain unresponsive to PRN medications alone, what might we use for pain control, with both standing & PRN components?**

| **Opiates in renal failure: Stoplight version** | | |
| --- | --- | --- |
| RED  (don’t use) | Morphine, codeine | Renally excreted & hepatically metabolized; codeine is metabolized into morphine 🡪 metabolites accumulate |
| RED-YELLOW  (non-preferred, use with caution) | Oxycodone | Renally excreted & hepatically metabolized 🡪 metabolites accumulate 🡪  lower dose and increase dosing interval |
| YELLOW-GREEN  (OK for use with caution) | Hydromorphone | Renally excreted & hepatically metabolized 🡪 metabolites accumulate, though less so than with above medications 🡪  lower dose and increase dosing interval |
| GREEN  (OK to use) | Fentanyl, methadone | Minimal renal excretion; watch QTc with methadone! |

| **Opiates in liver failure: Stoplight version** | | |
| --- | --- | --- |
| RED  (don’t use) | Tramadol, codeine | Hepatically metabolized, unpredictable |
| YELLOW (use with caution) | Morphine, oxycodone | Hepatically metabolized to active metabolites 🡪  lower dose & increase interval |
| YELLOW-GREEN  (OK for use with caution) | Hydromorphone | Hepatically metabolized to likely inactive metabolites 🡪 lower dose & increase interval |
|  | Fentanyl, methadone | Hepatically metabolized to inactive metabolites 🡪  lower dose & increase interval |

You go see Mr. L, and he complains of severe back pain, which he says is ~5/10. You decide to start him on standing acetaminophen, lidocaine patches, & oxycodone 2.5 – 5 mg Q4H PRN.

**What opioid side effects will you be looking out for, and which hold parameters will you include?**

| **Common Opioid Side Effects** | |
| --- | --- |
| **Side Effect** | **What to Do** |
|  |  |
|  |  |
|  |  |
|  |  |
|  |  |

**After admitting up a storm, you go home to get some sleep. The next night, just when you are ready to go grab some snacks, you get another page.**

PAGE: “Please call: Mr. L/703. In pain. Please assess.” - RN

When you look in the MAR, you see that he ultimately got oxycodone 5 mg PO x 8 yesterday and 0.4 mg IV hydromorphone x 4. When you examine the patient, he mentions that he has responded well to long-acting morphine in the past. He is feeling 8/10 pain currently.

**Since Mr. L’s pain is now severe, and is not controlled with the above medications, what could we use next for more effective long-acting pain control?**

**But first, given his increased pain, what do you also want to assess for/rule out (hint: something we rule out in any oncology patient with back pain & spinal metastases)?**

Once you’ve reassured yourself that he has no exam findings concerning for evolution of cord compression, you decide to write Mr. L for a PCA.

**Why might we put him on a PCA?**

**PART 3: NAUSEA, INSOMNIA, & ANXIETY MANAGEMENT**

**When you walk by later that evening, Mr. L’s pain is well-controlled; however, he comments that he’s had nausea since starting his chemotherapy.**

**What are the different types of anti-emetics, and what are benefits/side effects?**

| **Antiemetics** | | | |
| --- | --- | --- | --- |
| **Class** | **Drug**  **(Brand Name)** | **Specific Uses/Benefits** | **Side Effects** |
| 5-HT3 receptor antagonists |  |  |  |
| 5-HT2 receptor (& D2 receptor) antagonists |  |  |  |
| Dopamine receptor antagonists |  |  |  |
| Benzodiazepines |  |  |  |
| Anticholinergics (less commonly used) |  |  |  |

**You decide to treat Mr. L with ondansetron IV/PO panel. You check an EKG with a reassuring QTc of 420, and see that he’s not on other QTc-prolonging meds. You note in “Overnight Events” that you recommend a daily EKG if he receives frequent ondansetron.**

**When you check back in on him a few hours later, his nausea is resolved, but he can’t sleep. What are your first- & second-line choices for insomnia medications?**

First line insomnia meds:

Second line/less optimal choices for inpatients:

Meds that can help with both sleep & anxiety:

**You start Mr. L on standing nightly melatonin and add PRN 25-50 mg trazodone QHS. You have a quiet rest of the night, and the next morning, sign out & head off to your well-deserved vacation block.**

**PART 4: END OF LIFE**

**Several months later, you’re about to start oncology days. You notice that Mr. L is on your list again, and are excited to see him. Sadly, however, you see that he has declined clinically, & he had a PET-CT performed today, which shows significant progression of disease. You will need to discuss these results with the family tomorrow.**

**What mnemonic have you heard of to guide this (and other) serious discussions?**

**That afternoon you sit down with the patient and his family to discuss the results of his scan.**

****OPTIONAL****

**ROLE PLAY: Turn to your neighbor and discuss how you would go about conducting this family meeting, going through each step of the SPIKES mnemonic. Then, each person should take a turn role playing the intern, while the other person role plays the patient to practice having this conversation.**

**Following your conversation with Mr. L & his family, he decides to pursue CMO. Your team believes that his current symptoms can be managed in a hospice setting, and begin evaluation for inpatient versus outpatient hospice.**

**What are the requirements for hospice care?**

**What symptoms might patients experience toward the end of life, and how do we aim to treat these symptomatically?**

| **Common End of Life Symptoms** | |
| --- | --- |
| **Symptoms** | **What We Use** |
|  |  |
|  |  |
|  |  |
|  |  |
|  |  |

**When we talk about pain & palliative care, and end of life, it is inevitable that we touch upon debriefing & processing the death and illness that befalls many of our patients in the hospital. We are not going to actively discuss it today, but below, have provided some tip and resources; please know that there is a wealth of supports in place for processing, not the least of which is your fellow co-interns and your residents.**

When processing, we recommend:

- Talking to your co-interns and co-residents; we are some of each other’s best supports.
- Talking to nurses and other staff. Many nurses are very experienced with coping with loss & are often very close with patients’ and families. Some of us have found the best solace in reflecting on a patient’s life and death with a nurse who also cared for the patient.
- Talking to your friends & loved ones outside of medicine; while you can’t disclose HIPAA-related details, it is important to clue in your loved ones about what you are going through; otherwise, it can sometimes feel hard to connect with them when they don’t understand what you are going through
- Reaching out to someone for help. If you are having persistent thoughts about a patient’s death or other traumatic experience, reach out to one of the Chief Residents or members of the residency program’s leadership. Resources including everything from a good hug to therapy are available.
- Remember and honor your patients. Consider journaling.

**References:**

1. Baile WF, Buckman R, Lenzi R, Glober G, Beale EA, Kudelka AP. SPIKES-A six-step protocol for delivering bad news: Application to the patient with cancer. *The Oncologist*. 2000;5(4):302-311. doi:10.1634/theoncologist.5-4-302

**Pain Management and Palliative Care**

**Instructor Guide**

Objectives

At the conclusion of this activity, participants will be able to:

1. Describe multiple approaches to discussing code status at the time of hospital admission
2. Demonstrate an approach to initiating and up-titrating pain and nausea medications in the inpatient setting
3. Practice conversations about serious illness and end of life issues
4. Identify and treat common symptoms at the end of life

**Part 1: Code status**

You are on your first night of oncology night float, and you get your first admission. Mr. L is a 75M with metastatic prostate CA who is complaining of severe back pain. He has known spinal metastases. His physical exam and MRI rule out cord compression but do show new compression fractures at L4/L5. You finish your H&P and are about to head upstairs when you get the following page.

PAGE: “Don’t forget to confirm code status!” -Nightlfloat resident

**How do you approach code status in someone you just met?**

*Code Status is not the same as broader goals of care, but it can flow naturally from a discussion about the patient’s overall goals.  There are many ways to approach this; here is one framework.*

1. Normalize: *e.g., I have this conversation with every patient I meet in the hospital.*

*The way you normalize should consider disease acuity. If you are worried death could be imminent, honesty is important. You should page your resident if a fuller GOC conversation is emergently needed.*

2. Explain why: *e.g., I ask everyone this, to make sure that if you have wishes or concerns about your care, we are aware of them & respect them.*

3. Ask/assess*: e.g., Have you ever talked with your family or doctor about what you would you want done if you were to get very sick/much sicker? Some people tell me that they have thought about this and have expressed that they do or do not want certain lifesaving measures– other people have not thought about it before.*

*Note: Often, it is helpful to start broad, & then ask more specific questions – if your heart were to stop, would you want us to try to restart it with CPR or chest compressions? If you were unable to breathe on your own, would you want us to put in a breathing tube and use a machine to try to keep you alive?*

*If the patient has not thought about this previously, or cannot reach a decision, do not push them – your goal now is to determine whether they have previously thought about it, & if not, whether they have clear wishes/thoughts in this moment. If they are uncertain and there is no documentation to the contrary (e.g. a MOLST), the default should be full code, and you should circle back to have a longer discussion after the patient is admitted. Similarly, if you think a patient’s code status is not consistent with likelihood of reversibility of an intervention (e.g. unlikely to ever be extubated), this also should be re-addressed in a longer conversation soon.*

4. Reflect/Summarize: *e.g., “Based on what you’re saying, it sounds like you….”*

5. Document the order.

**PART 2: PAIN MANAGEMENT**

When you get back to the workroom, you get another page…

PAGE: “Please call: Mr. L/703 back pain. Needs hydromorphone.” - RN

When we think about pain control, we often talk about oral & topical therapies; when/how to use these is dictated by the type of pain & its location.

**What are the differences between neuropathic & nociceptive pain, and what types of therapies do we use for each?**

*1) Nociceptive pain: from stimuli that cause or threaten tissue damage, e.g. MSK, mechanical, compression, inflammation*

- *Medications of choice: non-opioid (acetaminophen, NSAIDs), opioid oral agents*
- *Specific diseases may respond especially well to specific therapies (e.g. colchicine & prednisone for gout)*

*2) Neuropathic pain: from central or peripheral nervous system disorder, e.g. DM, stroke, post-herpetic neuralgia*

- *Medications of choice: tricyclic antidepressants, SNRIs, gabapentin/pregabalin*

**What type of pain does Mr. L have?**

*Most likely nociceptive due to his spinal metastases, but it could also be neuropathic, for example Herpes Zoster.*

**If his pain is mild, which PRN agents could we use?**

- *Non-opioid analgesic: PRN acetaminophen or NSAID unless contraindication*
  - *Avoid NSAIDS in patients with AKI/CKD, post-MI, CHF, & bleeding diathesis*
  - *For appropriate patients, can trial IV acetaminophen if PO insufficient – takes effect in 5 – 10 minutes & peaks in about 15, which is faster than oral (~10 to >60 minutes)*

**What topical or anti-spasmodic adjuvants could we use to supplement?**

- *Topicals: lidocaine patch or ointment, capsaicin cream, topical NSAIDs (e.g. diclofenac)*
- *Heat therapy – OK for everyone!*
- *IF muscle spasm component, can also add low-dose muscle relaxants/antispasmodics: baclofen, cyclobenzaprine, tizanidine (wide range of side effects – up titrate with caution)*

**If he has moderate pain unresponsive to PRN medications alone, what might we use for pain control, with both standing & PRN components?**

- *Standing Acetaminophen (often written as Q8H or Q6H).*
  - *No renal/kidney dysfunction: Max 4g daily*
  - *Renal failure: Dosing intervals extended depending on GFR (e.g. no more often than Q8H if GFR <10 – see UpToDate for more detailed recommendations)*
  - *Cirrhosis or advanced liver disease: Max 2g daily*
- *NSAIDS – consider standing or PRN, IV (ketorolac) or PO*
  - *Dosing: Max 3.2g ibuprofen daily (800 ibuprofen Q6H)*
  - *Pro tip: IV ketorolac can provide tremendous relief & can be opioid-sparing*
  - *Not ideal as long-term therapy in patients with risk factors for AKI, as many of our patients have, but can use in the short term if safe in that patient*
- *Topical adjuvants*
- *Opioids: short-acting low-dose PRN, PO or IV depending on severity*

*Note: we do not recommend using opioid-acetaminophen combination products in the hospital (e.g., Percocet (oxycodone-acetaminophen) or Vicodin (hydrocodone-acetaminophen)) even if they’re home medications, given 1) risk of acetaminophen overdose if patients get acetaminophen + combo medication, and 2) ease of titrating individual components if written separately*

| **Opiates in renal failure: Stoplight version** | | |
| --- | --- | --- |
| RED  (don’t use) | Morphine, codeine | Renally excreted & hepatically metabolized; codeine is metabolized into morphine 🡪 metabolites accumulate |
| RED-YELLOW  (non-preferred, use with caution) | Oxycodone | Renally excreted & hepatically metabolized 🡪 metabolites accumulate 🡪  lower dose and increase dosing interval |
| YELLOW-GREEN  (OK for use with caution) | Hydromorphone | Renally excreted & hepatically metabolized 🡪 metabolites accumulate, though less so than with above medications 🡪  lower dose and increase dosing interval |
| GREEN  (OK to use) | Fentanyl, methadone | Minimal renal excretion; watch QTc with methadone! |

| **Opiates in liver failure: Stoplight version** | | |
| --- | --- | --- |
| RED  (don’t use) | Tramadol, codeine | Hepatically metabolized, unpredictable |
| YELLOW (use with caution) | Morphine, oxycodone | Hepatically metabolized to active metabolites 🡪  lower dose & increase interval |
| YELLOW-GREEN  (OK for use with caution) | Hydromorphone | Hepatically metabolized to likely inactive metabolites 🡪 lower dose & increase interval |
|  | Fentanyl, methadone | Hepatically metabolized to inactive metabolites 🡪  lower dose & increase interval |

You go see Mr. L, and he complains of severe back pain, which he says is ~5/10. You decide to start him on standing acetaminophen, lidocaine patches, & oxycodone 2.5 – 5 mg Q4H PRN.

**What opioid side effects will you be looking out for, and which hold parameters will you include?**

| **Common Opioid Side Effects** | |
| --- | --- |
| **Side Effect** | **What to Do** |
| *Somnolence, depressed RR* | *-Hold Parameters: hold for SBP<100, RR<12, AMS/somnolence, and page RC*  *-Narcan is your friend if any concern for opioid-induced respiratory depression!* |
| *Constipation (reduced peristalsis, longer GI transit time)* | *-Start a bowel regimen with opioids*  *-Standing Senna +/- polyethylene glycol with hold for loose stools is reasonable option* |
| *Delirium* | *-Can be precipitated by opiates as well as by pain*  *-Use your judgment in terms of which of these is driving delirium, & how to mitigate* |
| *Nausea/vomiting* | *-Trial changing opiates*  *-Antiemetics* |
| *Also: myoclonus, HPA axis depression (2’ adrenal insufficiency), pruritus, urinary retention … the list goes on* |  |

**After admitting up a storm, you go home to get some sleep. The next night, just when you are ready to go grab some snacks, you get another page.**

PAGE: “Please call: Mr. L. In pain. Please assess.” - RN

When you look in the MAR, you see that he ultimately got oxycodone 5 mg PO x 8 yesterday and 0.4 mg IV hydromorphone x 4. He is feeling 8/10 pain currently.

**Since Mr. L’s pain is now severe, and is not controlled with the above medications, what could we use next for more effective long-acting pain control?**

- *Starting a PCA – which residents can now order/write*

**But first, given his increased pain, what do you also want to assess for/rule out (hint: something we rule out in any oncology patient with back pain & spinal metastases)?**

*Make sure no new exam findings concerning for cord compression.*

Once you’ve reassured yourself that he has no exam findings concerning for evolution of cord compression, you decide to write Mr. L for a PCA.

**Why might we put him on a PCA?**

- *PCA allows for easy calculation of daily opioid requirements, avoids the peaks/valleys seen with PRN opioid IV boluses if patients are requiring frequent PRNs, and can give patients more control over pain management*
  - *Opiates: morphine, hydromorphone, fentanyl*

.

*FYI: for bony pain/cancer pain, steroids are often a useful adjunct, as well. We won’t discuss this right now, but something to keep in your back pocket when you are on oncology.*

**PART 3: NAUSEA, INSOMNIA, & ANXIETY MANAGEMENT**

**When you walk by later that evening, Mr. L’s pain is well-controlled; however, he comments that he’s had nausea since starting his chemotherapy.**

**What are the different types of anti-emetics, and what are particular benefits/side effects?**

| **Antiemetics** | | | |
| --- | --- | --- | --- |
| **Class** | **Drug**  **(Brand Name)** | **Specific Uses/Benefits** | **Side Effects** |
| 5-HT3 receptor antagonists | *Ondansetron* | *-First line for most pts*  *-Work well for chemo-induced nausea*  *-Oral has similar efficacy to IV* | *-HA, dizziness, constipation*  *-QTc prolongation* |
| 5-HT2 receptor (& D2 receptor) antagonists | *Olanzapine* | *-Can be given as sublingual*  *-Helps with anxiety/delirium* | *-Orthostatic hypotension, HA, drowsiness, extrapyramidal effects*  *-QTc prolongation (though less than other antipsychotics)* |
| Dopamine receptor antagonists | *-Prochlorperazine*  *-Chlorpromazine*  *-Metoclopramide*  *-Haloperidol* | *-Metoclopramide is prokinetic; used if gastroparesis*  *-Haloperidol is antipsychotic* | *-Anxiety/restlessness, dystonic reactions*  *-QTc prolongation* |
| Benzodiazepines | *Lorazepam* | *-NON-QTc-prolonging!* | *-Paradoxical agitation, delirium*  *-Somnolence* |
| Anticholinergics (less commonly used) | *Scopolamine patch* | *-Controls secretions* | *-Anticholinergic; can cause delirium* |

*FYI:* *Steroids work well for chemo-induced nausea, may be used/discussed on oncology*

**You decide to treat Mr. L with ondansetron IV/PO panel. You check an EKG with a reassuring QTc of 420 and see that he’s not on other QTc-prolonging meds. You note in “Overnight Events” that you recommend a daily EKG if he receives frequent ondansetron.**

**When you check back in on him a few hours later, his nausea is resolved, but he can’t sleep. What are your first- & second-line choices for insomnia medications?**

First line insomnia meds:

- *Standing QHS melatonin (no side effects!)*
  - *Consider ordering this PRN for all patients*
  - *Standing QHS for all elderly patients who are more susceptible to sleep-wake cycle dysregulation in the hospital*
- *PRN trazodone (anticholinergic & weak antidepressant with few side effects)*

Second line/less optimal choices for inpatients:

- *Diphenhydramine – sometimes used for younger patients with insomnia unresponsive to above agents, avoid in sick/elderly due to anticholinergic effects*
- *Zolpidem– we typically avoid in the hospital as can be very sedating, has associated fall risk*

Meds that can help with both sleep & anxiety:

- *Antipsychotics: quetiapine & olanzapine can both be used for anxiety/agitation, and may also provide a sedating effect*
- *Benzodiazepines treat anxiety & can promote sleep but need to use your clinical judgment given risk for paradoxical agitation, over sedation, respiratory depression*

**You start Mr. L on standing nightly melatonin and add PRN 25-50 mg trazodone QHS. You have a quiet rest of the night, and the next morning, sign out & head off to your well-deserved vacation block.**

**PART 4: END OF LIFE**

**Several months later, you’re about to start oncology days. You notice that Mr. L is on your list again and are excited to see him. Sadly, however, you see that he has declined clinically, & he had a PET-CT performed today, which shows significant progression of disease. You will need to discuss these results with the family tomorrow.**

**What mnemonic have you heard of to guide this (and other) serious discussions?**

*There are many. One that we find helpful is the SPIKES mnemonic, below.*^31^

*S: Setting up*

- *Where: make sure you have a good space for the discussion*
- *What: make sure you know the pertinent information (if there’s anything you don’t know during the meeting, tell the family you’ll look it up & let them know later – don’t guess!)*
- *Who: yourself, patient, think about also including: family, chaplain, SW, RN, etc.*

*P: Perception*

- *Assess how much the patient & family know; ask open-ended questions & let the patient tell you what he/she knows (e.g., “what is your understanding of your prostate cancer & your treatment for it?” “what have you been told so far about why we did the PET scan yesterday?”)*

*I: Invitation*

- *Asking patient’s permission to share the news. E.g., “I have some updates I’d like to share with you. Would that be OK?”*

*K: Knowledge*

- *Sharing information with the patient in plain language*
- *A brief warning shot can be useful (e.g., “I’m afraid I have some bad news to share,”) but do not belabor this*
- *Give lots of space for questions/clarification*
- *Repeat the key points & keep them to a minimum – if bad news, patients are unlikely to recall too many different pieces of information*

*E: Emotions*

- *Acknowledging & addressing the patient’s emotions (e.g., “I know this must be very difficult to hear”)*

*S: Strategy/Summary*

- *Summarize key points*
- *Set some concrete next steps – what are we doing, & when will we talk again*

**That afternoon you sit down with the patient and his family to discuss the results of his scan.**

****OPTIONAL****

**ROLE PLAY: Turn to your neighbor and discuss how you would go about conducting this family meeting, going through each step of the SPIKES mnemonic. Then, each person should take a turn role playing the intern, while the other person role plays the patient to practice having this conversation.**

**Following your conversation with Mr. L & his family, he decides to pursue CMO. Your team believes that his current symptoms can be managed in a hospice setting and begin evaluation for inpatient versus outpatient hospice.**

**What are the requirements for hospice care?**

*Six months of less life expectancy; note, patient does NOT have to be DNR/DNI or CMO to participate in hospice, & hospice does not entail cessation of life-sustaining therapies (e.g. antibiotics, fluids, can all still be given in hospice, though these may eventually be discontinued depending on further GOC conversations).*

**What symptoms might patients experience toward the end of life, and how do we aim to treat these symptomatically?**

| **Common End of Life Symptoms** | |
| --- | --- |
| **Symptoms** | **What We Use** |
| *Pain* | *Morphine, fentanyl, or hydromorphone gtt* |
| *Air hunger* | *-Opiates*  *-Fan blowing toward patient’s face*  *-Supplemental O2 (NC can be drying; some patients prefer humidified shovel masks, though this may feel claustrophobic for others)* |
| *Anxiety* | *Benzodiazepines* |
| *Delirium* | *Haloperidol, quetiapine, olanzapine* |
| *Secretions* | *Glycopyrrolate, scopolamine patch** |

**Secretions can be very troubling for families; remember that the patient is not clearing the secretions is because they’re not feeling them. Though the secretions make us feel uneasy, the patient is not uncomfortable.*

**When we talk about pain & palliative care, and end of life, it is inevitable that we touch upon debriefing & processing the death and illness that befalls many of our patients in the hospital. We are not going to actively discuss it today, but below, have provided some tip and resources; please know that there is a wealth of supports in place for processing, not the least of which is your fellow co-interns and your residents.**

When processing, we recommend:

- Talking to your co-interns and co-residents; we are some of each other’s best supports.
- Talking to nurses and other staff. Many nurses are very experienced with coping with loss & are often very close with patients’ and families. Some of us have found the best solace in reflecting on a patient’s life and death with a nurse who also cared for the patient.
- Talking to your friends & loved ones outside of medicine; while you can’t disclose HIPAA-related details, it is important to clue in your loved ones about what you are going through; otherwise, it can sometimes feel hard to connect with them when they don’t understand what you are going through
- Reaching out to someone for help. If you are having persistent thoughts about a patient’s death or other traumatic experience, reach out to one of the Chief Residents or a member of the residency program’s leadership.. Resources including everything from a good hug to therapy are available.

Remember and honor your patients. Consider journaling.

**References:**

1. Baile WF, Buckman R, Lenzi R, Glober G, Beale EA, Kudelka AP. SPIKES-A six-step protocol for delivering bad news: Application to the patient with cancer. *The Oncologist*. 2000;5(4):302-311. doi:10.1634/theoncologist.5-4-302
