# Supplementary material for: Getting up to Speed: A Resident-Led Inpatient Curriculum for New Internal Medicine Interns
Source: MedEdPORTAL. 2019 Dec 27;15:10866. doi: 10.15766/mep_2374-8265.10866 (PMC7012307; doi:10.15766/mep_2374-8265.10866)
Supplement: Supplementary file 1 — A. Intern Survey.docx B. Resident Survey.docx C. Acid-Base Disturbances.docx D. Antibiotics.docx E. Chest Pain.docx F. Safe Discharges.docx G. Gastrointestinal Bleeding and Pancreatitis.docx H. Inpatient Diabetes Management.docx I. Pain Management and Palliative Care.docx J. Shock and Vasopressors.docx [file mep-15-10866-s001.zip › J. Shock and Vasopressors.docx]

**Shock and Vasopressors**

Intern Guide

Objectives

At the conclusion of this activity, participants will be able to:

1. Differentiate among the types of shock
2. Identify appropriate vasopressor and inotrope use

Let’s start with the basics:

**What is shock?**

**What do you look for on physical exam?**

**And labs?**

**How do we classify shock?**

| ***Type*** | ***Exam*** | ***CVP/PCWP*** | ***CO/ScVO2*** | ***SVR*** |
| --- | --- | --- | --- | --- |
|  |  |  |  |  |
|  |  |  |  |  |
|  |  |  |  |  |
|  |  |  |  |  |

**What are our typical goals when treating someone in shock?**

**What are the medications we use?**

**Case 1**

Mr. L is a 64-year-old man with a PMH of bladder cancer s/p cystectomy, urostomy, and bilateral PCN placement presents from home with fever and altered mental status. His wife notes that over the past two days, he has been sleepier than usual and has had increasing confusion. She also noted that his urine was cloudy and malodorous. The morning of admission, she took his temperature at home and found it to be 101.7, so she brought him to the ED. There, vitals were T 101, HR 110, BP 104/51, RR 18, O2 96% RA. Blood and urine cultures were drawn and he was started on empiric antibiotics and admitted to Oncology.

Shortly following his transfer to the floor, you are paged that Mr. L’s BP is 80/40.

**What do you do next?**

Vitals are now T 101.8, HR 123, BP 80/40, RR 28, O2 94% RA. On exam, the patient is somnolent but arousable to loud voice. He is oriented only to himself. His skin is warm and flushed and he has normal cap refill. Cardiac auscultation reveals tachycardia but no murmurs. Pulmonary exam reveals short shallow respirations but no focal adventitious sounds. PCN and urostomy sites are draining cloudy dark yellow urine.

**What is the most likely etiology of his hypotension?**

**What data do you have to support this?**

**What treatment will you start with?**

You ask for 1 L bolus of LR and after confirming his most recent EF was 55%, you run it wide open. Unfortunately, his repeat BP after the bolus is 70/30. You hang another liter, call the ICU, and prepare to transfer to the MICU.

**The patient’s blood pressure does not improve with continued fluid resuscitation. What pressor will you choose to start?**

You have titrated the first-line pressor up to 30 mcg/min and the patient’s MAP is hovering around 55.

**What therapy will you start next?**

**What other adjunctive therapies do we consider in distributive shock?**

Urine cultures grow E coli, Klebsiella, and Enterococcus. He was transitioned from ceftriaxone to ampicillin-sulbactam and his percutaneous nephrostomy tubes were exchanged. He was able to wean off pressors and was eventually discharged on a course of amoxicillin-clavulanate.

**Case 2**

Mr. M is a 56-year-old man with PMH HTN and DM who initially presented to an OSH with chest pain and was found to have NSTEMI; LHC showed multivessel disease. While he was awaiting potential CABG, he developed sudden onset chest pain and shortness of breath. He was intubated and transferred to the CCU.

**How do we approach shock in the CCU?**

**What is tailored therapy?**

**What are our typical goals in tailored therapy?**

Upon transfer, the patient has a PA catheter in place. His MAP is 63, PCWP is 27, CI 1.7, and SVR 1800.

**How will you treat him?**

**How would you treat him differently if his MAP were <60?**

**What if the patient had a TTE which showed acute right heart failure?**

**Following your initial intervention, labs reveal lactate which is now 3.7 from 2.0 on transfer and CI is now 1.3.**

Following the next intervention, the patient’s repeat hemodynamics improve to show CI 2.3 and lactate 1.8.

A couple of days pass and with continued diuresis the patient’s CI is now consistently above 2.2 and his lactate has remained flat. His most recent PAC data is PCWP 18, CI 2.4, SVR 1600.

**How will you now optimize his hemodynamics?**

**References:**

1. Singer M, Deutschman CS, Seymour CW, et al. The Third International Consensus Definitions for Sepsis and Septic Shock (Sepsis-3). *JAMA*. 2016;315(8):801-810. doi:10.1001/jama.2016.0287

2. Semler MW, Self WH, Wanderer JP, et al. Balanced Crystalloids versus Saline in Critically Ill Adults. *N Engl J Med*. 2018;378(9):829-839. doi:10.1056/NEJMoa1711584

3. Rhodes A, Evans LE, Alhazzani W, et al. Surviving Sepsis Campaign: International Guidelines for Management of Sepsis and Septic Shock: 2016. *Intensive Care Med*. 2017;43(3):304-377. doi:10.1007/s00134-017-4683-6

4. Russell JA, Walley KR, Singer J, et al. Vasopressin versus norepinephrine infusion in patients with septic shock. *N Engl J Med*. 2008;358(9):877-887. doi:10.1056/NEJMoa067373

5. Annane D, Sébille V, Charpentier C, et al. Effect of treatment with low doses of hydrocortisone and fludrocortisone on mortality in patients with septic shock. *JAMA*. 2002;288(7):862-871. doi:10.1001/jama.288.7.862

6. Annane D, Renault A, Brun-Buisson C, et al. Hydrocortisone plus Fludrocortisone for Adults with Septic Shock. *N Engl J Med*. 2018;378(9):809-818. doi:10.1056/NEJMoa1705716

7. Sprung CL, Annane D, Keh D, et al. Hydrocortisone therapy for patients with septic shock. *N Engl J Med*. 2008;358(2):111-124. doi:10.1056/NEJMoa071366

8. Keh D, Trips E, Marx G, et al. Effect of Hydrocortisone on Development of Shock Among Patients With Severe Sepsis: The HYPRESS Randomized Clinical Trial. *JAMA*. 2016;316(17):1775-1785. doi:10.1001/jama.2016.14799

9. Venkatesh B, Finfer S, Cohen J, et al. Adjunctive Glucocorticoid Therapy in Patients with Septic Shock. *N Engl J Med*. 2018;378(9):797-808. doi:10.1056/NEJMoa1705835

**Shock and Vasopressors**

**Instructor Guide**

Objectives

At the conclusion of this activity, participants will be able to:

1. Differentiate among the types of shock
2. Identify appropriate vasopressor and inotrope use

Let’s start with the basics:

**What is shock?**

*A state of decreased tissue perfusion resulting in decreased tissue oxygen delivery and inadequate oxygen utilization resulting in end-organ damage. It is initially reversible but can quickly progress to irreversible damage.*

**What do you look for on physical exam?**

*Hypotension (SBP<90 or drop by >40 mmHg)*

*Increased pulse pressure vs decreased pulse pressure*

*Tachycardia*

*Tachypnea*

*Whether skin is warm and flushed vs cool and clammy*

*Oliguria (UOP < 0.5 cc/kg/hr.)*

*Altered mental status*

*We talk about the patient’s MAP, which is defined as 2/3 DBP + 1/3 SBP. We are typically concerned about a patient whose MAP is less than 65.*

**And labs?**

*Lactate*

*BUN/Cr*

*LFTs*

*Troponin and BNP*

*CBC w/diff*

*Coags*

*ABG*

**How do we classify shock?**

*Distributive (Sepsis, neurogenic, anaphylaxis, adrenal crisis)*

*Hypovolemic (GI losses, renal losses, bleeding)*

*Cardiogenic (MI, heart failure)*

*Obstructive (tamponade, PE, tension pneumothorax)*

| ***Type*** | ***Exam*** | ***CVP/PCWP*** | ***CO/ScVO2*** | ***SVR*** |
| --- | --- | --- | --- | --- |
| *Distributive* | *Warm and dry* | *↓* | *↑ or normal* | *↓↓* |
| *Hypovolemic* | *Cold and dry* | *↓* | *↑↑* | *↑* |
| *Cardiogenic* | *Cold and wet* | *↑* | *↑↑* | *↑* |
| *Obstructive* | *Cold and dry* | *↑* | *↑↑* | *↑* |

**What are our typical goals when treating someone in shock?**

*MAP > 65*

*UOP > 0.5 cc/kg/hr.*

*SvO2 >70*

*Normalization of lactate*

**What are the medications we use?**

**Case 1**

Mr. L is a 64-year-old man with a PMH of bladder cancer s/p cystectomy, urostomy, and bilateral PCN placement presents from home with fever and altered mental status. His wife notes that over the past two days, he has been sleepier than usual and has had increasing confusion. She also noted that his urine was cloudy and malodorous. The morning of admission, she took his temperature at home and found it to be 101.7, so she brought him to the ED. There, vitals were T 101, HR 110, BP 104/51, RR 18, O2 96% RA. Blood and urine cultures were drawn and he was started on empiric antibiotics and admitted to Oncology.

Shortly following his transfer to the floor, you are paged that Mr. L’s BP is 80/40.

**What do you do next?**

*Go assess the patient immediately. Review recent medications and orders on the way to see the patient as well as pertinent information like EF (if known) and ask for a full set of vitals as soon as you get there. When you get in the room, confirm IV access and examine the patient with emphasis on the findings delineated above.*

Vitals are now T 101.8, HR 123, BP 80/40, RR 28, O2 94% RA. On exam, the patient is somnolent but arousable to loud voice. He is oriented only to himself. His skin is warm and flushed and he has normal cap refill. Cardiac auscultation reveals tachycardia but no murmurs. Pulmonary exam reveals short shallow respirations but no focal adventitious sounds. PCN and urostomy sites are draining cloudy dark yellow urine.

**What is the most likely etiology of his hypotension?**

*Most likely from sepsis, which is a form of distributive shock.*

**What data do you have to support this?**

*This is a good opportunity to discuss the evolving ways in which sepsis has been defined. We used to think of sepsis as two or more SIRS criteria (1. Temperature > 38.3 C or < 36.0 C; 2. Heart rate > 90 beats per minute; 3. Respiration > 20 per minute; 4. White blood cell count > 12 or < 4 x 10^3^/µL or > 10% bands) plus infection, but this was not sensitive enough. We now think of infection plus organ dysfunction (most recently defined in 2016 as “a life-threatening organ dysfunction due to a dysregulate host response to infection”*^1^*), which has been quantified in the SOFA score. The SOFA score describes dysfunction in CNS, cardiovascular, respiratory, renal, and coagulation systems. The quick SOFA, or qSOFA, may be a point of confusion in your groups. The qSOFA looks at altered mental status (GCS<15), respiratory rate > 22, and systolic blood pressure <* *100. The qSOFA is used for prognosis, NOT diagnosis. A score of 2 or higher near the onset of infection was associated with greater risk of death or prolonged ICU stay.*^1^

**What treatment will you start with?**

*Fluids. Although we often reflexively ask for normal saline in medicine, a large randomized trial from Feb 2018 (the SMART trial) demonstrated that the use of balanced crystalloids (like LR) for IVF resuscitation resulted in a lower rate of the composite outcome of death, new RRT, or persistent renal dysfunction when compared with NS.*^2^

*Data have shown that resuscitating with 30cc/kg over the first 3 hours is appropriate for patients with distributive shock, like this patient has.*^3^ *Assuming the patient is 75 kg, he should receive at least 2.25 L of fluid upfront for resuscitation (including what was administered in the ED).*

You ask for 1 L bolus of LR and after confirming his most recent EF was 55%, you run it wide open. Unfortunately, his repeat BP after the bolus is 70/30. You hang another liter, call the ICU, and prepare to transfer to the MICU.

**The patient’s blood pressure does not improve with continued fluid resuscitation. What pressor will you choose to start?**

*Norepinephrine is considered first line in septic shock. It has a rapid onset of action, high potency, and short half-life.*

You have titrated the first-line pressor up to 30 mcg/min and the patient’s MAP is hovering around 55.

**What therapy will you start next?**

*There are several second line agents for septic shock. Vasopressin is most often added as a second agent at a fixed dose of 0.04 units/min. The thought is that some patients with hyperkinetic distributive shock may develop a vasopressin deficiency. The VASST trial in 2008 showed that the addition of vasopressin was safe but did not show any mortality benefit.*^4^ *Epinephrine is also considered a second line agent but can be associated with increased risk of arrhythmias.*^3^

**What other adjunctive therapies do we consider in distributive shock?**

*There has been controversy about the addition of corticosteroids. One trial in 2002 (“Annane” trial) found a mortality benefit of IV hydrocortisone and fludrocortisone for patients with evidence of adrenal insufficiency on ACTH stimulation testing; a second trial by this group in 2018 again reported lower 90-day mortality in patients treated with steroids.*^5,6^ *Subsequent trials (CORTICUS, HYPRESS, and ADRENAL) showed faster time to reversal of shock but no mortality benefit.*^7–9^ *Because data are mixed, steroids are often added when fluids and pressors do not restore hemodynamic stability (i.e., as a last resort).*

Urine cultures grow E coli, Klebsiella, and Enterococcus. He was transitioned from ceftriaxone to ampicillin-sulbactam and his percutaneous nephrostomy tubes were exchanged. He was able to wean off pressors and was eventually discharged on a course of amoxicillin-clavulanate.

**Case 2**

Mr. M is a 56-year-old man with PMH HTN and DM who initially presented to an OSH with chest pain and was found to have NSTEMI; LHC showed multivessel disease. While he was awaiting potential CABG, he developed sudden onset chest pain and shortness of breath. He was intubated and transferred to the CCU.

**How do we approach shock in the CCU?**

*In the CCU, patients often demonstrate primarily cardiogenic shock or a mixed shock picture with both distributive and cardiogenic components. For this reason, we often make use of a pulmonary artery catheter to pursue “tailored therapy.”*

**What is tailored therapy?**

*Tailored therapy refers to the use of a pulmonary artery catheter to measure individual components of a patient’s hemodynamics and pursue specific goals. We typically measure MAP, PCWP, RAP, CI, and SVR and select vasopressors and inotropes accordingly.*

**What are our typical goals in tailored therapy?**

*MAP 60-65 mmHg*

*PCWP 10-14 mmHg in decompensated HF, 14-18 mmHg in acute MI*

*RAP < 8 mmHg*

*CI > 2.2 L/min/m2*

*SVR 1000-1200 dynes/sec/cm-5*

Upon transfer, the patient has a PA catheter in place. His MAP is 63, PCWP is 27, CI 1.7, and SVR 1800.

**How will you treat him?**

*Since his MAP is preserved, we do not have to start with a vasopressor. In this patient, we could start with an inotrope like dobutamine. Given that his wedge his high, we will also diurese him concurrently.*

**How would you treat him differently if his MAP were <60?**

*In this case, we would likely start with a vasopressor like norepinephrine to “defend the MAP.” Only when the MAP is >60 would we then add an inotrope and work on weaning the vasopressor.*

**What if the patient had a TTE which showed acute right heart failure?**

*Right heart failure is challenging to treat. Patients are often in a tenuous balance of optimal preload – they are “preload dependent,” but hypervolemia may decrease cardiac output. First choice pressors are typically phenylephrine or vasopressin because PVR is less affected than SVR. For contractility, milrinone is often the first choice given that it vasodilates both systemic and pulmonary arterial circulation.*

**Following your initial intervention, labs reveal lactate which is now 3.7 from 2.0 on transfer and CI is now 1.3.**

*At this point we would likely add another inotrope like milrinone to try to augment cardiac output.*

Following the next intervention, the patient’s repeat hemodynamics improve to show CI 2.3 and lactate 1.8.

A couple of days pass and with continued diuresis the patient’s CI is now consistently above 2.2 and his lactate has remained flat. His most recent PAC data is PCWP 18, CI 2.4, SVR 1600.

**How will you now optimize his hemodynamics?**

*At this point, we would likely work on weaning the inotropes and adding vasodilators such as nitroglycerin, and then PO afterload reduction such as hydralazine or captopril.*

**References:**

1. Singer M, Deutschman CS, Seymour CW, et al. The Third International Consensus Definitions for Sepsis and Septic Shock (Sepsis-3). *JAMA*. 2016;315(8):801-810. doi:10.1001/jama.2016.0287

2. Semler MW, Self WH, Wanderer JP, et al. Balanced Crystalloids versus Saline in Critically Ill Adults. *N Engl J Med*. 2018;378(9):829-839. doi:10.1056/NEJMoa1711584

3. Rhodes A, Evans LE, Alhazzani W, et al. Surviving Sepsis Campaign: International Guidelines for Management of Sepsis and Septic Shock: 2016. *Intensive Care Med*. 2017;43(3):304-377. doi:10.1007/s00134-017-4683-6

4. Russell JA, Walley KR, Singer J, et al. Vasopressin versus norepinephrine infusion in patients with septic shock. *N Engl J Med*. 2008;358(9):877-887. doi:10.1056/NEJMoa067373

5. Annane D, Sébille V, Charpentier C, et al. Effect of treatment with low doses of hydrocortisone and fludrocortisone on mortality in patients with septic shock. *JAMA*. 2002;288(7):862-871. doi:10.1001/jama.288.7.862

6. Annane D, Renault A, Brun-Buisson C, et al. Hydrocortisone plus Fludrocortisone for Adults with Septic Shock. *N Engl J Med*. 2018;378(9):809-818. doi:10.1056/NEJMoa1705716

7. Sprung CL, Annane D, Keh D, et al. Hydrocortisone therapy for patients with septic shock. *N Engl J Med*. 2008;358(2):111-124. doi:10.1056/NEJMoa071366

8. Keh D, Trips E, Marx G, et al. Effect of Hydrocortisone on Development of Shock Among Patients With Severe Sepsis: The HYPRESS Randomized Clinical Trial. *JAMA*. 2016;316(17):1775-1785. doi:10.1001/jama.2016.14799

9. Venkatesh B, Finfer S, Cohen J, et al. Adjunctive Glucocorticoid Therapy in Patients with Septic Shock. *N Engl J Med*. 2018;378(9):797-808. doi:10.1056/NEJMoa1705835
